# Targeting Telomere Shelterin Protein TPP1 with Elbasvir: Induction of Autophagy and Suppression of Esophageal Cancer Tumorigenesis

**DOI:** 10.2174/0118715206393619250911115554

**Published:** 2025-10-09

**Authors:** Maoju Tang, Miyuan Yang, Jiling Wen, Xiao Liu, Lei Xu, Qiang Ma, Xiaowu Zhong, Xiaolan Guo

**Affiliations:** 1 Department of Clinical Laboratory, Affiliated Hospital of North Sichuan Medical College, Nanchong 637000, P.R. China;; 2 School of Laboratory Medicine, North Sichuan Medical College, Nanchong 637000, P.R. China;; 3 Department of Clinical Laboratory, Beijing Anzhen Nanchong Hospital of Capital Medical University & Nanchong Central Hospital, Nanchong 637000, P.R. China;; 4 Translational Medicine Research Center, North Sichuan Medical College, Nanchong 637000, P.R. China

**Keywords:** Esophageal cancer, telomere shelterin protein, elbasvir, AKT-mTOR, autophagy, immunohistochemistry

## Abstract

**Introduction:**

Esophageal cancer often develops insidiously, with most cases diagnosed at an advanced stage. Currently, the pathogenesis of esophageal cancer remains unclear, treatment outcomes are poor, and the five-year survival rate is low. To tackle the significant clinical challenges of difficult diagnosis and unfavorable prognosis, it is crucial to actively investigate the disease's pathogenesis. This study explored the involvement of telomere shelterin protein TPP1 in the pathogenesis of esophageal cancer and identified potential therapeutic agents for its treatment.

**Methods:**

The expression level of TPP1 protein in 54 pairs of esophageal cancer tissues and paired adjacent tissues was detected * via * immunohistochemistry. The impact of TPP1 silencing and Elbasvir administration on the growth of KYSE150 and TE1 esophageal cancer cell lines was assessed utilizing Cell Counting Kit-8 and colony formation assays. Cell migration was assessed through Transwell and scratch assays. Fluorescence microscopy was employed to observe autophagosome formation, while flow cytometry measured the fluorescence intensity of autophagy markers LC3 and P62 in TPP1-silenced KYSE150 and TE1 cells. Western blotting was utilized to examine the alterations in TPP1, the AKT-mTOR signaling pathway, autophagy-related proteins, and other associated proteins.

**Results:**

TPP1 levels were notably elevated in esophageal squamous cell carcinoma tissues relative to adjacent normal tissues. Suppression of TPP1 substantially reduced the growth and movement of esophageal cancer cells *in vitro*, while triggering autophagy *via* the AKT-mTOR signaling pathway, highlighting TPP1’s cancer-promoting function in esophageal cancer.

**Discussion:**

Elbasvir effectively suppressed the growth and spread of KYSE150 and TE1 cell lines *in vitro*, downregulating TPP1 protein expression in relation to time and dosage. Additional investigations revealed that Elbasvir also inhibited the AKT-mTOR signaling axis and induced autophagy by targeting TPP1. Notably, rescue experiments demonstrated that 3-MA could reverse the inhibitory effects on proliferation, migration, and autophagy induced by TPP1 silencing or Elbasvir treatment in KYSE150 and TE1 cells.

**Conclusion:**

TPP1 emerges as a compelling diagnostic indicator and a potential treatment focus in esophageal cancer, with Elbasvir offering promise as a novel therapeutic agent.

## 1 INTRODUCTION

Esophageal Cancer (ESCA), an exceedingly invasive malignancy with a grim prognosis, remains one of the most under-researched cancers globally despite its high mortality [1-3]. According to GLOBOCAN 2020, it ranks seventh in occurrence and sixth in mortality globally [4]. The predominant histological subtypes are squamous cell carcinoma and adenocarcinoma, with esophageal squamous cell carcinoma accounting for approximately 90% of instances in China [5-7]. The emergence of esophageal squamous cell carcinoma is strongly linked to various risk factors, including gender, ethnicity, smoking, alcohol consumption, diet, nutritional status, and genetic predisposition [8-11]. Although multiple treatment modalities exist-such as surgery, radiotherapy, chemotherapy, neoadjuvant therapy, immunotherapy, and biologically targeted therapy-targeted therapies are relatively uncommon and generally show limited efficacy [12-17]. Consequently, the outlook for ESCA continues to be unfavorable, with a 5-year survival rate of only 15-20% [18, 19], largely due to its complex pathogenesis.

The shelterin complex protein TPP1 is a crucial element of the telomere-protective protein complex, which collaborates with five other core proteins (TRF1, TRF2, RAP1, TIN2, and POT1) to preserve telomere stability and integrity [20-22]. Emerging research has highlighted TPP1's significant role in tumorigenesis, progression, and treatment response [23-25]. Elevated TPP1 expression facilitates the uncontrolled proliferation and immortality of tumor cells by enhancing telomerase recruitment and preventing telomere shortening [26-28]. TPP1 engages with human Telomerase (TERT) through the TEL-patch domain, suggesting that ligands targeting this region could disrupt the TPP1-telomerase association and trigger senescence in cancer cells [29-32]. Furthermore, reduced TPP1 expression has been observed in patients undergoing immunosuppressive therapy, which may potentially increase cellular immortalization capacity [33, 34]. Nevertheless, the function of TPP1 in ESCA treatment remains largely unexplored. This investigation aims to clarify TPP1’s influence on ESCA progression and spread, thereby providing a theoretical foundation for understanding its pathogenesis and identifying small-molecule drugs targeting TPP1. In this investigation, a collection of medications sanctioned by the Food and Drug Administration (FDA) underwent screening, leading to the identification of Elbasvir as a novel anticancer agent targeting TPP1. Elbasvir, a complex organic heterotetracyclic compound, is commonly used in combination with Grazoprevir to inhibit Hepatitis C Virus (HCV) Non-Structural Protein 5A (NS5A), treating chronic hepatitis C genotype 1 or 4 infections in adults [35]. Grazoprevir/elbasvir (Zepatir) is the latest Direct-acting Antiviral Agent (DAA) combination tablet approved by the U.S. FDA for HCV management [36]. Elbasvir exhibits potent NS5A inhibitory activity with picomolar efficacy against most HCV genotypes [37, 38], offering antiviral and hepatoprotective benefits, along with high efficacy, safety, and reliability [39, 40]. To date, the anticancer potential of Elbasvir in ESCA and the mechanisms involved remain unclear.

This study seeks to explore TPP1 expression in ESCA and its cancer-promoting function, as well as investigate Elbasvir’s regulatory effects on TPP1 expression in ESCA cells, unveiling the underlying molecular pathways. These findings aim to establish a new theoretical foundation for the potential use of Elbasvir as an anti-ESCA therapeutic.

## 2 MATERIALS AND METHODS

### 2.1 Immunohistochemical Staining

Paraffin-embedded tissue samples from 54 ESCA cases and their corresponding adjacent non-cancerous tissues were obtained from the Affiliated Hospital of North Sichuan Medical College (Nanchong, China) and preserved at -4°C. The Ethics Committee of the Affiliated Hospital of North Sichuan Medical College granted sanction for the study (No. 2022ER461-1). To prepare the sections for analysis, paraffin was removed using xylene, followed by rehydration through a sequence of alcohol solutions with varying concentrations. The retrieval of antigens was achieved by heating the sections under high pressure in citrate buffer for 3 minutes. After sealing the edges with a histochemical pen, the specimens underwent a 10-minute dark incubation in 3% H_2_O_2_, followed by a 30-minute blocking step using 5% BSA at 37°C. Overnight incubation at 4°C in darkness was conducted with a mouse anti-TPP1 antibody (1:100; Invitrogen, USA). The next day, sections were treated with HRP-labeled anti-rabbit IgG (Boster, China) at 37°C for 1 hour. Visualization was achieved through the application of a 2-minute DAB reagent (1:20; Boster, China), which was halted with water, and then counterstained with hematoxylin for 30 seconds. Differentiation was achieved using 1% hydrochloric acid in alcohol for 2 seconds. Dehydration was executed *via* a graded series of alcohols, and sections were cleared in xylene for 10 minutes each before being mounted with neutral gum. The slides were then examined under a microscope and photographed.

### 2.2 Cell Culture

Human esophageal squamous carcinoma cell lines KYSE150 and TE1 were acquired from the Translational Medicine Research Center of North Sichuan Medical College and kept in DMEM medium supplemented with 10% fetal bovine serum at 37°C in a 5% CO_2_ atmosphere. DMEM and FBS were sourced from Vivacell (Shanghai, China), while Elbasvir was purchased from TargetMol (China). The 3-MA reagent was obtained from MedChemExpress (MCE, China).

### 2.3 Lentiviral Infection and Transfection

Plasmids containing shTPP1 (#1, #2, and #3) and shNC were procured from Genechem (Shanghai, China), and the construction of these plasmids was completed by a previous research team. To confirm knockdown efficiency, qRT-PCR was executed to validate TPP1 mRNA levels in KYSE150 and TE1 cells with TPP1 knockdown, using primers synthesized by Chengdu Bioengineering (Supplementary Table **S1**). Western Blot (WB) confirmed the protein level of TPP1 knockdown (1:1000, Cell Signaling Technology, USA). Total RNA was procured utilizing Vazyme Biotech reagents and reverse transcribed with HiScript Ⅲ SuperMix for qPCR (Vazyme Biotech, China). PCR amplification was performed employing Taq Pro Universal SYBR qPCR Master Mix (Vazyme Biotech, China) under the following conditions: initial denaturation at 95°C for 30 seconds, annealing at 62°C for 5 seconds, and extension at 72°C for 30 seconds, over a total of 40 cycles. The results were quantified using the 2^-ΔΔCT^ method.

### 2.4 Western Blot

Cells were lysed in RIPA buffer comprising a protease inhibitor at a 1:100 ratio to prepare a protein sample of 40 μg/20 μL. The protein components were fractionated *via* SDS-PAGE and subsequently transferred to Polyvinylidene Fluoride (PVDF) membranes under chilled conditions. The membranes underwent blocking with 5% milk powder for 2 hours at ambient temperature and were then exposed to the primary antibody overnight at 4°C. Following this step, the membranes were treated with the secondary antibody for 1 hour at room temperature. Protein bands were detected utilizing an enhanced chemiluminescence imaging system (Vilber Fusion FX7, France).

### 2.5 Screening of TPP1 Candidate Inhibitors

TPP1 protein crystals exhibiting high integrity, resolution, and ligand-binding specificity were chosen from the Protein Data Bank (https://www.rcsb.org)[41]. After a thorough comparison, the structure 2I46 [42] was chosen for its suitability with FDA-approved drug ligands. The docking sites were determined based on the ligand positions, and a virtual screening of small molecules from the FDA database was conducted using DOCK 6.9 software [43]. Screening parameters included a cavity span of 10 Å, box dimensions of 6 Å at the edges, a box center located at (102.758, 16.157, 12.187) Å, and a box size of (38.873, 33.203, 39.23) Å.

### 2.6 Molecule Docking

The crystal structure of the TPP1 receptor was retrieved from the Protein Data Bank and underwent optimization at the bond level, including hydrogenation, disulfide bond assignment, hydrogen bond assignment, and energy minimization, using the QuickPrep module in the MOE software [44]. The molecular structures of the compounds were constructed using MOE’s build tool, and their 3D conformations were generated *via* energy minimization. Multiple conformations of the compounds were generated through a conformational search. The processed TPP1 structural models were then docked with Elbasvir in various conformations using the DOCK module of MOE software, with the top 100 docking results being stored for further analysis [45].

### 2.7 Drug Affinity Responsive Target Stability (DARTS)

First, 1 × 10^6^ TE1 and KYSE150 cells were digested and lysed by adding RIPA buffer on ice for 10 minutes, followed by centrifugation at 12,000 grams at 4°C for 10 minutes to determine protein concentration. Subsequently, 400 μM Elbasvir was added to the extracted protein stock solution and incubated at ambient conditions for 2 hours. The resulting mixture then underwent enzymatic digestion with pronase for 20 minutes, followed by the addition of PMSF to halt the digestion reaction. Protein samples were prepared according to the determined concentration and system specifications [46], and TPP1 protein levels were analyzed *via* WB.

### 2.8 Cell Viability Assay

Human ESCA cell lines KYSE150 and TE1 were seeded into 96-well plates at a concentration of 3 × 10^3^ cells per well in DMEM comprising 10% fetal bovine serum. After 24 hours, the cells were treated with varying concentrations of Elbasvir, or with an equivalent volume of DMSO as a control, for 48 hours, or treated with 40 μM Elbasvir at different time points. Following treatment, 100 μL of Cell Counting Kit-8 (CCK-8) reagent (Shanghai Bitai Biotechnology Co., Ltd.) was introduced to each well, and the plates were kept at 37°C with 5% CO_2_ for 2 hours. Absorbance was then ascertained at 450 nm utilizing a Sunrise(A)™ Enzyme Labeling Instrument (TECAN, NC), with all experiments performed in triplicate.

### 2.9 Plate Colony Formation Assay

 For the colony formation assay, KYSE150 and TE1 cells were plated in 6-well plates at a concentration of 1 × 10^3^ cells per well. Following 3-5 days of incubation, the medium was exchanged, and cells were exposed to the corresponding concentration of Elbasvir or an equivalent volume of DMSO. The culture was continued until visible colonies appeared, typically within 1-2 weeks. The medium was then discarded, cells were rinsed thrice with PBS, fixed using methanol for 10 minutes, stained with crystal violet solution for 10 minutes, rinsed, and air-dried. Images were captured employing a GT-S650 scanner (Epson).

### 2.10 Transwell Assay

For the migration assay, KYSE150 and TE1 cells were seeded in 6-well plates at a concentration of 1 × 10^5^ cells per well and exposed to DMSO or 40 μM Elbasvir after 24 hours. Following 48 hours, the cells were dissociated using 0.25% trypsin, suspended in a serum-free medium, and adjusted to a concentration of 5 × 10^5^ cells/mL. A 200 μl portion of cell suspension was introduced into the upper compartment of a Transwell, while 500 μl of DMEM supplemented with 10% fetal bovine serum was placed in the lower compartment. After a 24-hour incubation period, the upper compartment was extracted, and the cells were fixed in methanol for 10 minutes, stained with crystal violet solution, rinsed, air-dried, and then imaged under a microscope.

### 2.11 Scratch Assay

Scratch assays were conducted using 12-well plates, with one scratch model per well. ESCA cells KYSE150 and TE1 were introduced into the side chambers of the scratch models at a density of 5 × 10^6^ cells/ml, with 100 μl added to each side chamber. After 24 hours, or when the cells reached approximately 90-100% confluence, the scratch models were carefully extracted, and floating cells were rinsed off using PBS. Images of the initial wound (0 hours) were captured at this stage. Subsequently, the cells underwent incubation in a serum-free DMEM medium containing 40 μM Elbasvir dissolved in DMSO for 24 hours, and wound healing was monitored and photographed.

### 2.12 GFP-LC3 Plasmid Transfection

For the TPP1 knockdown experiments, KYSE150 and TE1 cells were resuspended and plated in 6-well dishes at a concentration of 5 × 10^5^ cells per well. Following 24 hours, the cells were transfected with the GFP-LC3 plasmid (provided by the Hormel Institute, University of Minnesota, USA). To prepare the transfection mix, two sterile 5 mL EP tubes were filled with 300 μl of serum-free DMEM. Into one tube, 4 μg of the target plasmid was added, and into the other, 12 μl of lipofectamine was added. After gentle mixing, the Lipofectamine mixture was combined with the plasmid mixture and left to stand at ambient conditions for 15 minutes. The medium in the 6-well plates was substituted with serum-free medium, and the transfection mixture was introduced to the cells. After a 6-hour incubation period in DMEM containing 10% serum, the medium was replaced. After 48 hours, images were procured under a fluorescence microscope to count the number of transfected cells.

### 2.13 Cell Fluorescence

Stable TPP1-silenced KYSE150 and TE1 cell lines were digested with trypsin, resuspended in medium, rinsed twice with PBS, and the supernatant was removed. The cells were then fixed with a fixative for 15 minutes, followed by treatment with a permeabilizing agent and incubation with P62 or LC3 fluorescent antibody reagents at 37°C for 30 minutes. Fluorescence intensity was subsequently ascertained utilizing flow cytometry.

## 3 STATISTICAL ANALYSIS

All *in vitro* experiments were conducted in triplicate, with data denoted as mean ± SD. Statistical significance was determined utilizing Student’s t-tests or one-way Analysis of Variance (ANOVA) *via* Graphpad Prism 9.5 software. Differences were deemed statistically significant when the *P*-value was below 0.05.

## 4 RESULTS

### 4.1 TPP1 Exhibits Elevated Expression in ESCA and Promotes Cell Multiplication and Migration

The expression levels of TPP1 in ESCA tissues and normal tissues were analyzed through the GEPIA cancer database. It was found that the mRNA expression level of TPP1 was significantly higher in ESCA tissues compared with normal tissues (Fig. **1A**). The expression of TPP1 protein in ESCA tissues was evaluated using Immunohistochemistry (IHC), revealing that TPP1 expression was notably elevated in ESCA tissues relative to adjacent non-cancerous tissues (Fig. **1B**). Statistical analysis confirmed a significant difference in TPP1 expression between ESCA tissues and adjacent tissues (Fig. **1C**), indicating TPP1’s heightened presence in ESCA. The expression level of TPP1 protein was examined in esophageal normal cells HET1A and ESCA cell lines KYSE150, Eca109, KYSE410, and TE1. The results showed that the basal level of TPP1 was the highest in ESCA cells (KYSE150 and TE1), which were subsequently selected for the experiment (Fig. **S1**). To investigate the role of TPP1 in ESCA, we silenced TPP1 in the KYSE150 and TE1 ESCA cell lines (Fig. **1D** and **E**). The results showed that the expression of the TPP1 protein was successfully knocked down (Fig. **S2**). The findings indicated that TPP1 suppression considerably hindered these cells’ proliferation and migration *in vitro* (Fig. **1F**-**I**). Specifically, TPP1 knockdown markedly diminished the colony formation capacity and proliferation activity of KYSE150 and TE1 cells relative to the control cohort (shNC) (Fig. **1F**, **G**). Furthermore, scratch and Transwell assays demonstrated that TPP1 silencing significantly impaired the migration capacity of KYSE150 and TE1 cells (Fig. **1H**, **I**). These observations imply that TPP1 promotes ESCA cell proliferation and migration, supporting its carcinogenic role in ESCA.

### 4.2 TPP1 Suppresses Autophagy via the AKT-mTOR Signaling Pathway

Previous studies have indicated that TPP1 knockdown induces autophagy in hepatocellular carcinoma cells; however, its role in regulating autophagy in ESCA cells remains to be elucidated [47]. To investigate this possibility, we introduced GFP-LC3 plasmids into KYSE150 and TE1 cells with stable TPP1 suppression. Fluorescence microscopy revealed a marked rise in autophagosome count, evidenced by numerous green dots in cells with reduced TPP1 expression (Fig. **2A**). Flow cytometry further confirmed that the fluorescence intensity of the autophagy markers LC3 and P62 was significantly higher in TPP1-silenced KYSE150 and TE1 cells relative to the control cohort (Fig. **2B** and **C**). These observations suggest that TPP1 silencing induces autophagy in KYSE150 and TE1 cells. Moreover, WB analysis demonstrated that the phosphorylation levels of mTOR and AKT (p-mTOR and p-AKT) were considerably diminished in TPP1-silenced cells, whereas the total levels of mTOR and AKT remained unchanged. Additionally, the downstream targets of the AKT-mTOR signaling pathway, p-70S6K1, and p-4EBP1, were downregulated, while autophagy-related proteins LC3 and P62 were upregulated (Fig. **2D**). The quantitative analysis of proteins also supports this result (Fig. **S3**). These results indicate that TPP1 may inhibit autophagy in ESCA cells *via* the AKT-mTOR signaling pathway.

For subsequent experiments, shTPP1-3 was selected based on its silencing efficiency. The autophagy blocker 3-methyladenine (3-MA) was used in rescue experiments to further explore the role of autophagy. The results demonstrated that 3-MA treatment significantly downregulated LC3 expression in TPP1-silenced KYSE150 and TE1 cells (Fig. **3A**). Protein quantitative analysis also supports this finding (Fig. **S4**), indicating that 3-MA effectively inhibits autophagy induced by TPP1 silencing. Further investigation into the effects of autophagy inhibition on the proliferation and migration of TPP1-silenced ESCA cells revealed that cell viability and colony formation were diminished in the TPP1-silenced cells exposed to 3-MA compared to those without 3-MA treatment (Fig. **3B** and **C**). Additionally, cell migration distance and the quantity of cells traversing the Transwell chamber were further decreased (Fig. **3D** and **E**). These results confirm that 3-MA enhances the suppressive impact of TPP1 silencing on the proliferation and motility of KYSE150 and TE1 cells. Overall, the experiments suggest that inhibiting autophagy increases the sensitivity of TPP1-silenced ESCA cells, thus augmenting the restraining effects on their growth and migratory capacity.

### 4.3 Elbasvir Downregulates the Expression of TPP1, Mediating the Proliferation and Metastasis in ESCA

A high-throughput drug screening platform was utilized to identify 2,845 molecular inhibitors targeting the TPP1 protein (Supplementary Table **S2**). Among the top 10 candidate inhibitors, none had been previously reported to affect cancer. WB analysis was executed to assess the effects of these inhibitors on TPP1 protein expression in KYSE150 and TE1 cells. The findings suggested that 10 μM Elbasvir effectively inhibited TPP1 protein expression, while the other candidate inhibitors did not show significant effects on TPP1 expression (Fig. **4A**). Protein quantification analysis is shown in Fig. (**S5A**). This finding suggests that Elbasvir may act as a drug targeting TPP1. Subsequently, molecular docking was employed to predict the potential binding sites of Elbasvir on TPP1 (Fig. **4B**). The analysis identified Glu168, Glu169, Glu171, Lys170, Arg208, and Asp166 as the residues most likely involved in the binding of Elbasvir to TPP1 (Fig. **4C**). These interactions were further validated under cellular conditions using Drug Affinity Responsive Target Stability (DARTS) assays. Consistent with the predictions, the presence of Elbasvir increased the stability of TPP1 protein, protecting it from proteolysis by pronase in KYSE150 and TE1 cells (Fig. **4D**). Protein quantification analysis is shown in Fig. (**S5B**). These outcomes further support the notion that Elbasvir acts as a direct inhibitor of TPP1.

The impact of Elbasvir in ESCA cells was subsequently investigated. CCK-8 assays indicated that Elbasvir markedly diminished the viability of KYSE150 and TE1 cells, with the inhibitory effect increasing in a time- and dose-dependent manner (Fig. **5A**). Elbasvir significantly downregulated TPP1 protein levels in a concentration- and time-dependent manner, with the downregulation becoming more pronounced as both concentration and treatment duration increased (Fig. **5B** and **C**), The protein was quantitatively analyzed, and the results further confirmed this (Fig. **S6**). In colony formation assays, 40 μM Elbasvir significantly suppressed the proliferation of KYSE150 and TE1 cells (Fig. **5D**). Additionally, scratch and Transwell assays indicated that Elbasvir markedly impaired the migratory capacity of these cells (Fig. **5E** and **F**). Collectively, these results indicate that TPP1, as a direct target, mediates the anticancer effects of Elbasvir in ESCA.

### 4.4 Elbasvir Induces Autophagy in ESCA via the AKT-mTOR Signaling Pathway

Following 48 hours of treatment with 10 μM Elbasvir, a notable decrease in the expression of the target protein TPP1 was detected in both cell lines. Additionally, the expression levels of mTOR, p-mTOR, AKT, and p-AKT were markedly reduced relative to the control cohort, indicating suppression of the AKT-mTOR signaling axis. Correspondingly, the levels of p-70S6K and p-4EBP1 were decreased, while autophagy-associated proteins LC3 and P62 were upregulated (Fig. **6A**). The quantitative analysis of proteins also supports this result (Fig. **S7**). These results suggest that Elbasvir induces autophagy in KYSE150 and TE1 cells by inhibiting proteins involved in the AKT-mTOR signaling pathway.

WB analysis of the autophagy marker protein LC3 showed that the addition of 3-MA significantly reduced LC3 levels in both the DMSO and Elbasvir-treated cohorts, indicating that 3-MA effectively inhibits Elbasvir-induced autophagy (Fig. **6B**). Protein quantification analysis is shown in Fig. (**S8**). When Elbasvir was combined with 3-MA, a significant enhancement in the inhibition of colony formation, cell proliferation, and migration abilities of KYSE150 and TE1 cells was observed compared to treatment with Elbasvir alone (Fig. **6C**-**F**). This enhancement of the suppressive impact on cell multiplication and motility by 3-MA suggests that impeding autophagy may heighten the responsiveness of ESCA cells to Elbasvir.

## 5 DISCUSSION

In this investigation, the expression of the telomere-protective protein TPP1 in ESCA cells was analyzed through immunohistochemical staining, which confirmed its tumor-promoting function in ESCA cells. Our experiments demonstrated that silencing TPP1 inhibits the proliferation and metastasis of ESCA cells, while also inducing autophagy *via* the AKT-mTOR signaling pathway. Additionally, the autophagy inhibitor 3-MA was shown to enhance the inhibitory effects of TPP1 silencing on cell proliferation and migration, suggesting that autophagy suppression can heighten the responsiveness of ESCA cells to Elbasvir. These findings underscore the carcinogenic role of TPP1 in ESCA cells and highlight its involvement in key regulatory pathways.

Telomeres are critical regulators of cellular senescence and immortality [48, 49]. In normal cells, telomere shortening leads to cellular aging, whereas telomere elongation in malignant tumor cells contributes to unchecked cellular proliferation [48, 50]. Telomeres consist of telomerase and associated protein complexes, with the telomere-protective protein complex being composed of six core proteins, among which TPP1 serves a crucial function in preserving telomere integrity and stability [51]. TPP1 is known to be highly expressed in various tumors that exhibit unlimited proliferative potential [52, 53]. Previous studies have shown elevated TPP1 expression in human colorectal cancer, where it protects telomeres from DNA damage and confers resistance to radiation therapy [54]. Furthermore, mutations in the telomere POT1-TPP1 complex have been linked to telomere lengthening, genomic instability, and increased susceptibility to cancers, such as familial glioma, melanoma, and chronic lymphocytic leukemia [55, 56]. TPP1 mutations can disrupt interactions with TIN2, impair telomerase recruitment, and shorten telomere length, thereby increasing the risk of colorectal cancer [57]. In this cohort study, TPP1 demonstrates elevated expression levels within ESCA specimens. However, due to time limitations, we were unable to collect patient prognosis data. Notably, the main functional domain of TPP1, known as TPP1-OB, can inhibit telomerase recruitment to telomeres, leading to telomere shortening. This reduction in telomere length is associated with decreased cancer cell proliferation, G1 cell cycle arrest, and increased apoptosis. Moreover, TPP1-OB enhances the sensitivity of cancer cells to the chemotherapy drug paclitaxel, making it a promising novel therapeutic target [58]. Our findings further support the notion that TPP1 plays a significant oncogenic role in ESCA, as its high expression in ESCA tissues is linked to enhanced tumor cell proliferation and metastasis. Consequently, TPP1 represents a potential new target for the diagnosis and treatment of ESCA. 

Drug repositioning is a strategic approach that identifies new therapeutic uses for existing or investigational drugs beyond their originally approved indications [59]. This method offers significant advantages, including a reduced risk of failure, shorter timelines for drug discovery and development, lower investment requirements, and the potential to uncover new drug targets and pathways [60, 61]. Dihydroartemisinin (DHA), a derivative of artemisinin primarily used as a first-line treatment for malaria, has recently been shown to exhibit anticancer activity in esophageal and liver cancers [62]. Similarly, chloroquine, another antimalarial drug, has been shown to induce autophagy, thereby inhibiting the growth of ESCA cells *in vitro* [63, 64]. Building on this concept, a molecular docking study was conducted to screen 2,845 FDA-approved and marketed small-molecule compounds as potential TPP1 inhibitors. The top 10 candidates, ranked by Grid_Score through DOCK 6.9 software, were further evaluated using WB analysis. Among these, Elbasvir was identified as significantly downregulating TPP1 expression. Subsequent experiments confirmed that Elbasvir reduces TPP1 protein levels in a time- and concentration-dependent manner. Molecular docking and DARTS assays suggested that Elbasvir directly binds to TPP1, highlighting its potential as a TPP1 inhibitor. Elbasvir, known as an NS5A inhibitor, is commonly used in combination with Grazoprevir (an NS3/4A inhibitor) for the treatment of chronic HCV infection [65, 66]. It effectively inhibits NS5A with picomolar activity against most HCV genotypes, exhibiting an EC50 value of 2.2 pM [67]. At a concentration of 50 pM, Elbasvir strongly inhibits the assembly of new viral replicase complexes, demonstrating potent activity across multiple HCV genotypes [68]. Despite its well-established antiviral properties, prior research has not indicated substantial antineoplastic effects in malignancies [69-71]. In this study, Elbasvir was shown to significantly downregulate TPP1 protein expression and suppress the proliferation and metastasis of ESCA cells. Collectively, these results support the potential therapeutic application of Elbasvir for the treatment of individuals with ESCA.

TPP1 has been associated with the regulation of the tumor cell cycle, particularly during the G2/M phase, where its ectopic expression is controlled by cell cycle-dependent translation, thereby influencing cell growth and proliferation [72, 73]. In Mouse Embryonic Fibroblasts (MEFs), the deletion of TPP1 triggers a DNA damage response and induces G2/M cell cycle arrest [74]. Our previous research has shown that inhibiting the activity of the telomere-protective protein TPP1 reduces both proliferation and the DNA damage response in ESCA cells [75]. It has also been reported that a telomere crisis can activate autophagic cell death, with telomere dysfunction in human fibroblast cell lines rapidly inducing elevated autophagy flux [76]. Additionally, the selective PI3K inhibitor 3-MA is known to inhibit autophagy formation [77, 78]. Studies have further demonstrated that Akt may directly regulate telomere protection by promoting the homodimerization of TPP1, leading to an increased concentration of TPP1 at telomeres, a process governed by cell growth signaling pathways through Akt activation [79]. In the context of ESCA, TPP1 has been shown to influence downstream autophagy levels. Silencing TPP1 was found to mediate the AKT-mTOR signaling pathway, thereby inducing autophagy. Similarly, Elbasvir was shown to significantly inhibit the AKT-mTOR signaling pathway and induce autophagy in ESCA cells. Furthermore, 3-MA enhanced the inhibitory effects on proliferation, migration, and autophagy in ESCA cells following TPP1 silencing or Elbasvir treatment. Collectively, these results suggest that Elbasvir downregulates the oncogene TPP1, inhibits autophagy accumulation induced by AKT-mTOR signaling, and ultimately suppresses the progression of ESCA.

## CONCLUSION

This study demonstrates that TPP1 is significantly overexpressed in ESCA and plays a critical oncogenic role. Genetic knockdown of TPP1 suppressed ESCA cell proliferation and metastasis by inhibiting the AKT-mTOR signaling axis and subsequently activating autophagy. Building upon these findings, we identified Elbasvir as a novel small-molecule inhibitor targeting TPP1 through comprehensive drug screening. Our results revealed that Elbasvir effectively downregulates TPP1 protein expression in a concentration-dependent manner. Mechanistically, Elbasvir exerts its anti-tumor effects by modulating the TPP1/AKT-mTOR pathway, thereby inducing autophagic cell death and significantly impairing the proliferative and metastatic capacity of ESCA cells. The specificity of this mechanism was further validated in rescue experiments, where the autophagy inhibitor 3-MA effectively reversed both TPP1 silencing-mediated and Elbasvir-induced autophagy, confirming the pivotal role of autophagy in the observed anti-cancer effects. While these findings establish Elbasvir as a promising therapeutic candidate for ESCA, several aspects warrant further investigation. First, the precise molecular interactions between Elbasvir and TPP1 require structural characterization. Second, the potential off-target effects and *in vivo* efficacy of Elbasvir need evaluation in preclinical models. Finally, the therapeutic synergy between Elbasvir and conventional ESCA treatments merits exploration. This study not only elucidates TPP1's oncogenic function in ESCA but also provides a pharmacological strategy for targeted therapy. Future research should focus on translating these findings into clinical applications while deepening our understanding of TPP1's role in esophageal carcinogenesis.

## Figures and Tables

**Fig. (1):**
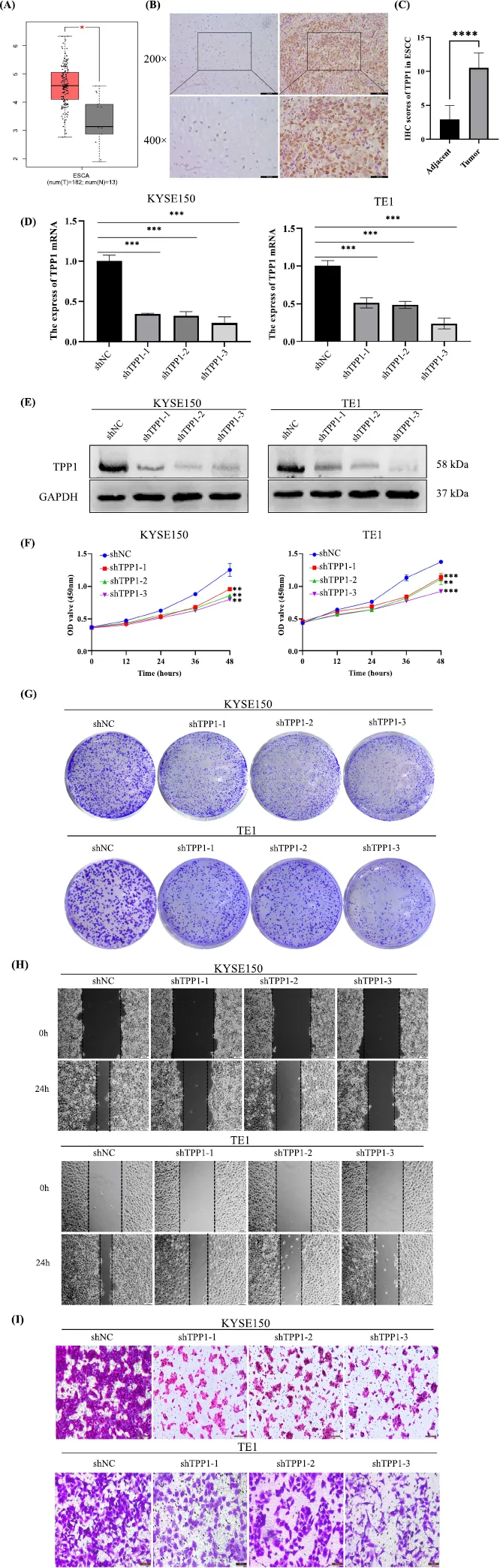
TPP1 serves as an oncogenic gene. (**A**) Expression levels of TPP1 mRNA in patients with esophageal cancer based on the TCGA database * *p* < 0.05. (**B**) The expression of TPP1 protein in cancerous and paracancer tissues of clinical patients with esophageal cancer (×200, ×400). (**C**) The staining scores of TPP1 protein in esophageal cancer and paracancer tissues were detected by immunohistochemical staining (54 paired), **** *p* < 0.0001. (**D**) Efficiency verification of mRNA levels and protein levels after knocking down the TPP1 protein *** *p* < 0.001. (**E**) Efficiency verification of protein levels after knocking down TPP1 protein. (**F**) CCK-8 detects the effect of TPP1 knockdown on KYSE150 and TE1 cell viability ** *p* < 0.01, *** *p* < 0.001, **** *p* < 0.0001. (**G**) The effect of TPP1 knockdown on the proliferative ability of KYSE150 and TE1 cells was detected by a cloning experiment. (**H**) The effect of TPP1 knockdown on the wound healing ability of KYSE150 and TE1 cells was detected by scratch assay. (**I**) Transwell assay to examine the effect of TPP1 knockdown on KYSE150 and TE1 cell migration ability.

**Fig. (2):**
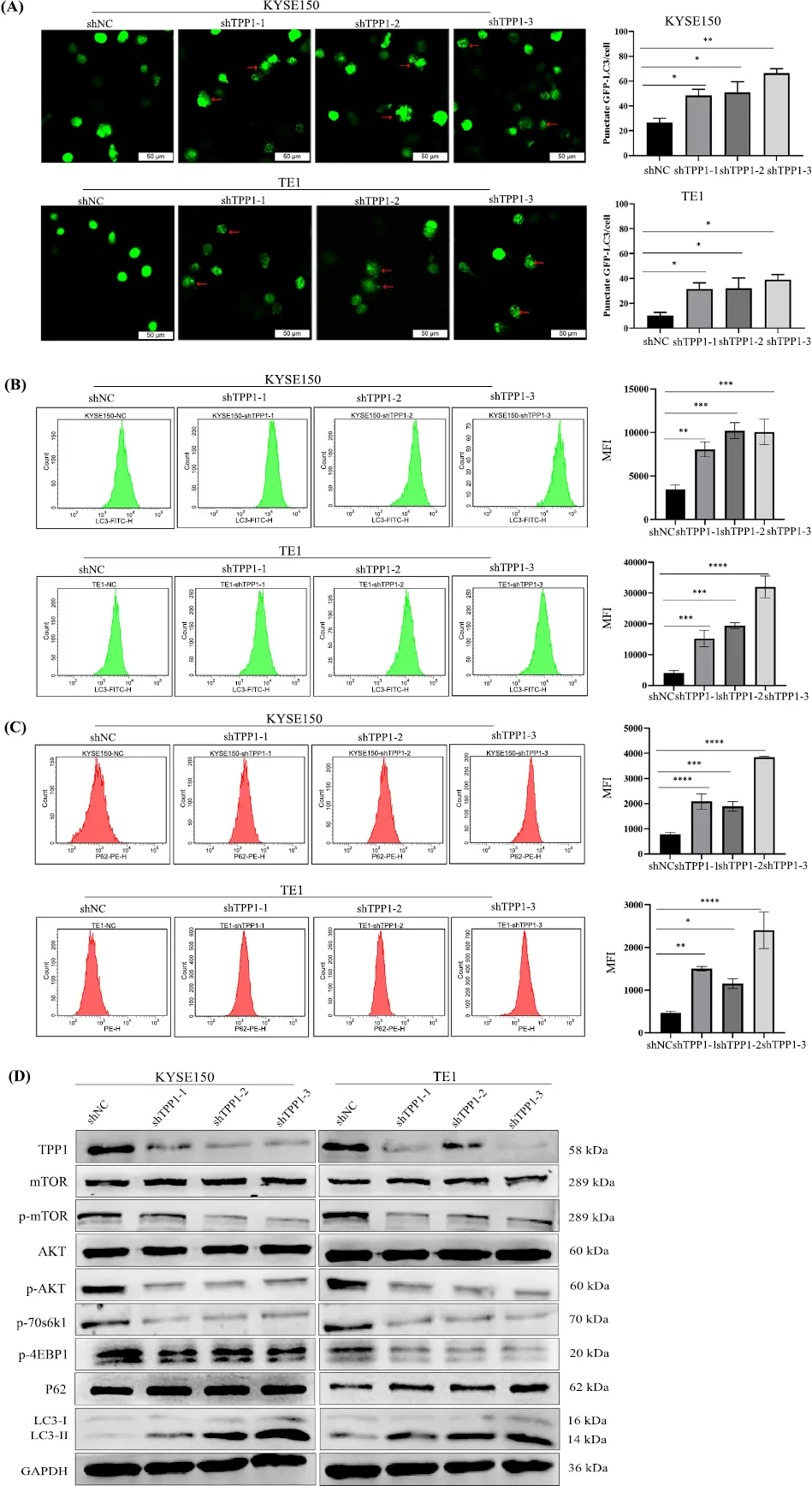
TPP1 inhibits autophagy *via* the AKT-mTOR signaling pathway. (**A**) The changes of autophagy after TPP1 knockdown were observed *via* fluorescence microscopy (the red arrows indicate autophagosomes) * *p* < 0.05, ** *p* < 0.01. (**B-C**) Flow cytometry was used to detect the levels of autophagy molecules P62 and LC3. (**D**) Western blot analysis was performed to detect the expression of AKT-mTOR signaling pathway and autophagy-related proteins downstream after TPP1 knockdown *p < 0.05, **p < 0.01, ***p < 0.001, ****p < 0.0001.

**Fig. (3):**
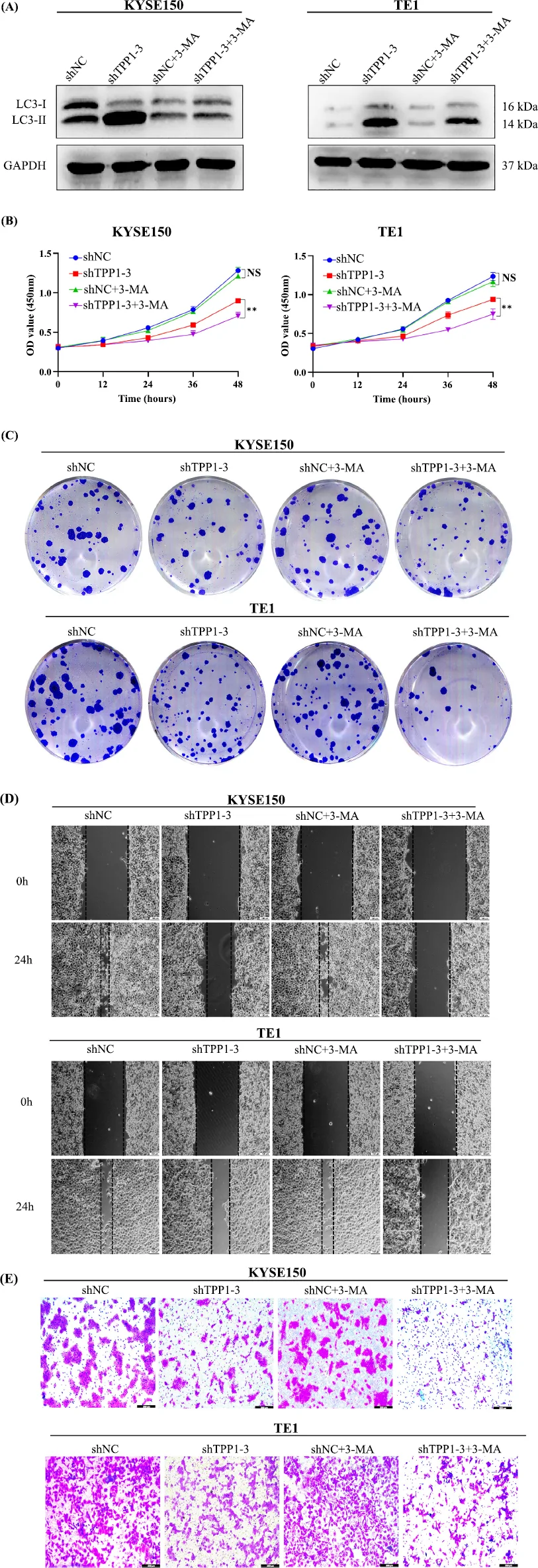
3-MA reverses autophagy induced by TPP1 knockdown. (**A**) Western blot analysis of 3-MA interference in the expression of autophagy molecule LC3 after TPP1 knockdown. (**B**) The effect of 3-MA intervention on KYSE150 and TE1 cell viability after TPP1 knockdown was detected by CCK-8, NS indicates *p* > 0.05, ** *p* < 0.01. (**C**) The effect of 3-MA intervention on the proliferative ability of KYSE150 and TE1 cells after TPP1 knockdown was detected *via* a cloning experiment. (**D**) Scratch test was performed to detect the effect of 3-MA intervention on the wound healing ability of KYSE150 and TE1 cells after TPP1 knockdown. (**E**) Transwell assay was used to examine the effect of 3-MA intervention on the migration ability of KYSE150 and TE1 cells after TPP1 knockdown.

**Fig. (4):**
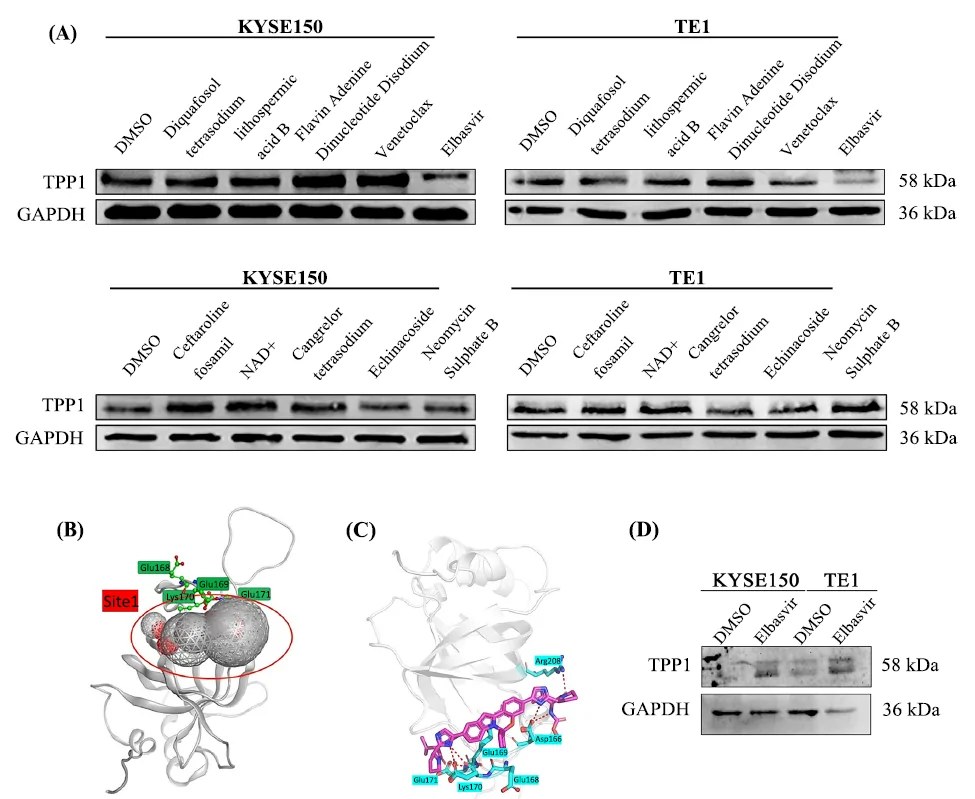
Molecular docking and screening for the targeted drug Elbasvir. (**A**) Western blot analysis of the expression of TPP1 protein after the postadministration of ten candidate target drugs. (**B**) Prediction of binding sites of the TPP1 protein. (**C**) Overall diagram of the interaction between Elbasvir and the 2I46 crystal structure of the TPP1 protein. (**D**) Target stability assay for drug affinity reaction used to detect the binding ability of Elbasvir to TPP1 protein under the action of 1:10,000 strepase protease. Note: Key amino acids in Site1 of TPP1 protein are marked in green. The amino acid residue at Site 1 in the 2I46 crystal structure of TPP1 protein is marked in blue-green. Elbasvir is marked in magenta. The red dashed line represents hydrogen Key.

**Fig. (5):**
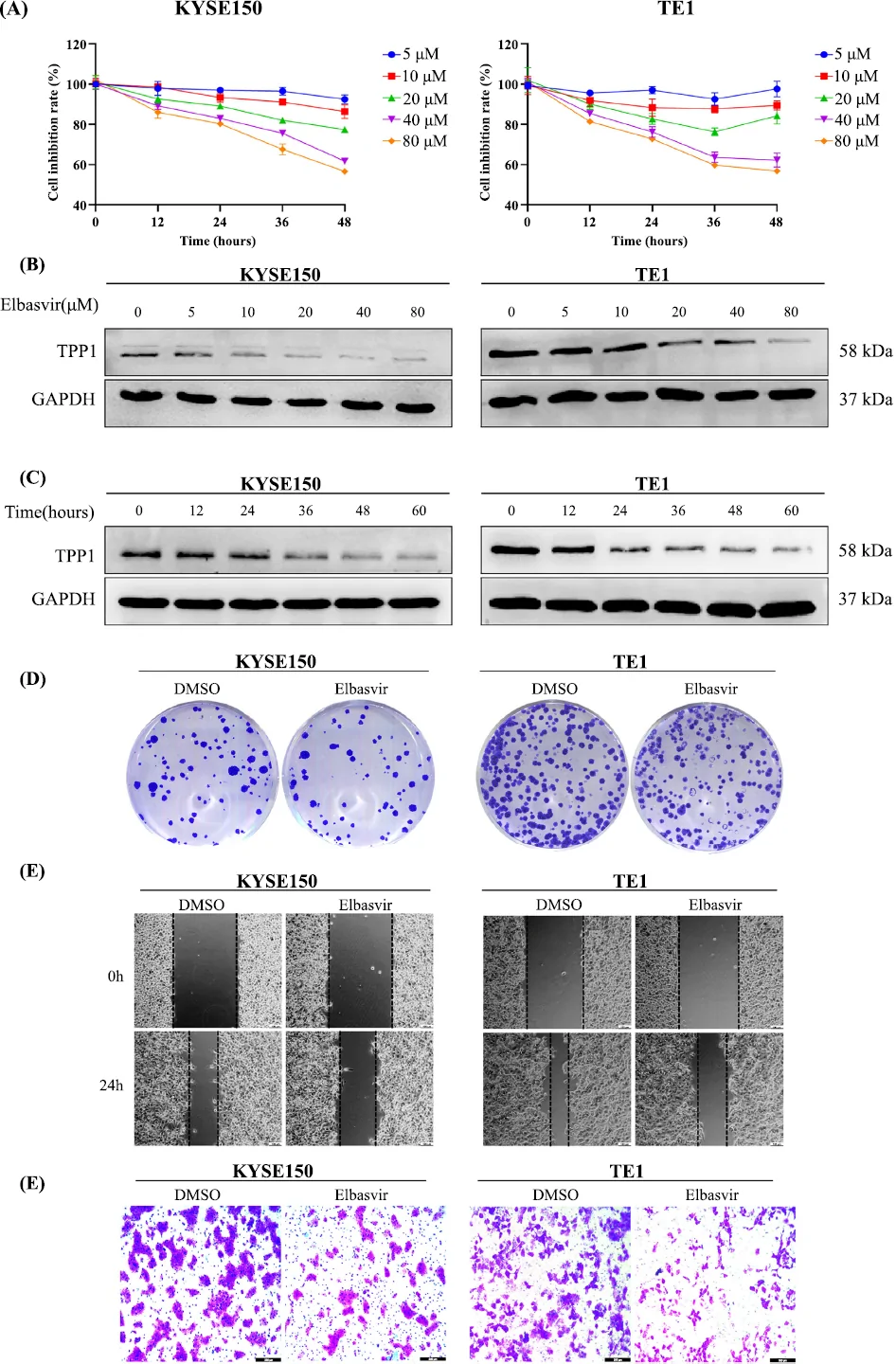
Elbasvir inhibits the growth of esophageal cancer. (**A**) Changes in cell viability after 48 hours of treatment with DMSO and Elbasvir by CCK-8. (**B**) Western blot was used to detect the expression of TPP1 protein after the treatment of esophageal cancer with different concentrations of Elbasvir. (**C**) Western blot was used to detect the expression of TPP1 protein after Elbasvir treatment of esophageal cancer at different times. (**D**) The proliferation ability of cells after DMSO and Elbasvir treatment for 48 hours was detected *via* a cloning assay. (**E**) The wound healing ability of cells treated with DMSO and Elbasvir for 48 hours was detected through the scratch assay. (**F**) Transwell assay was used to detect cell migration after 48 hours of DMSO and Elbasvir.

**Fig. (6):**
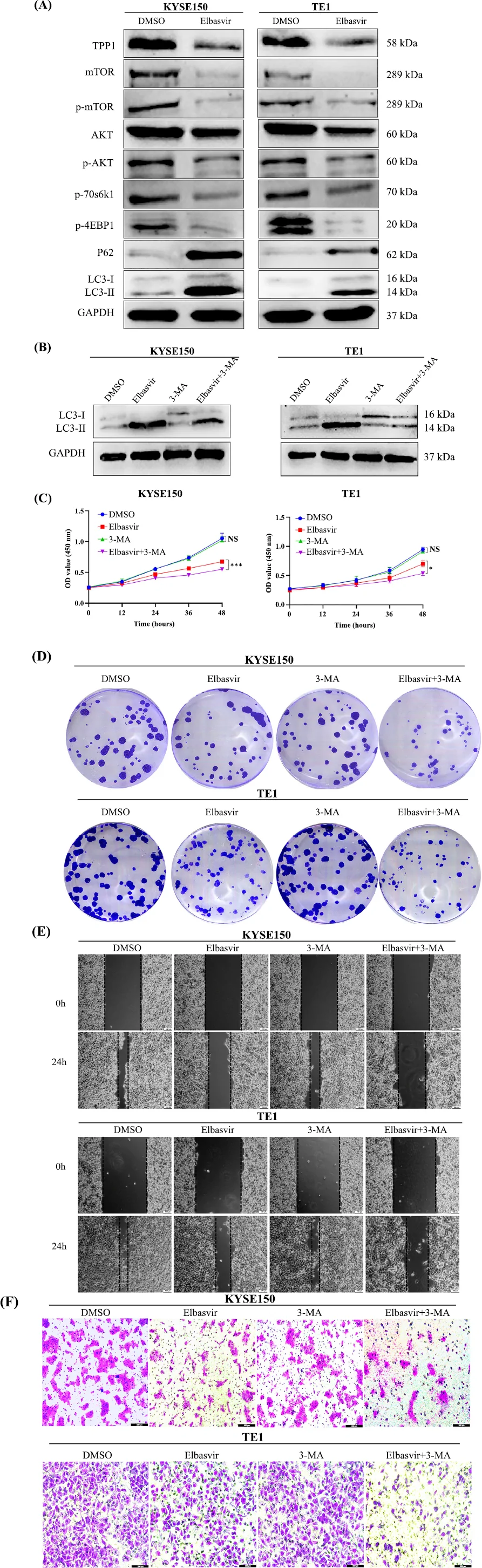
Elbasvir mediates autophagy induced by AKT-mTOR, and 3-MA partially reverses the process. (**A**) Western blot analysis was performed to detect the levels of AKT-mTOR signaling pathway and autophagy-related proteins after Elbasvir treatment for 48 hours. (**B**) Western blot assay to detect the expression level of autophagy protein LC3 after 3-MA pretreatment, treated with Elbasvir for 48 hours. (**C**) The effects of Elbasvir treatment for 48 hours on KYSE150 and TE1 cell viability were detected by CCK-8, NS indicates *p* > 0.05, *p < 0.05, ***p < 0.001. (**D**) The effect of Elbasvir treatment on the proliferative ability of KYSE150 and TE1 cells after 48 hours was detected *via* a cloning assay. (**E**) Scratch test was used to detect the effect of Elbasvir treatment on the wound healing ability of KYSE150 and TE1 cells after 48 hours. (**F**) Transwell assay was used to detect the effect of Elbasvir treatment on KYSE150 and TE1 cell migration ability after 48 hours.

## Data Availability

The authors confirm that the data supporting the findings of this study are available within the article and its supplementary materials.

## LIST OF ABBREVIATIONS

ANOVA: One-way Analysis of Variance
CCK-8: Cell Counting Kit-8
DAA: Direct-Acting Antiviral Agent
DARTS: Drug Affinity Responsive Target Stability
DHA: Dihydroartemisinin
ESCA: Esophageal Cancer
FDA: Food and Drug Administration
HCV: Hepatitis C Virus
IHC: Immunohistochemistry
MEFs: Mouse Embryonic Fibroblasts
NS5A: Non-Structural Protein 5A
PVDF: Polyvinylidene Fluoride
TERT: Human Telomerase
WB: Western Blot
