# Supplementary material for: Targeting Telomere Shelterin Protein TPP1 with Elbasvir: Induction of Autophagy and Suppression of Esophageal Cancer Tumorigenesis
Source: Anticancer Agents Med Chem. 2025 Oct 9;26(12):1154–71. doi: 10.2174/0118715206393619250911115554 (PMC13621370; doi:10.2174/0118715206393619250911115554)
Supplement: Supplementary file 1 [file ACAMC-26-12-1154_SD1.pdf]

SUPPLEMENTARY MATERIAL

Targeting Telomere Shelterin Protein TPP1 with Elbasvir: Induction of Autophagy and Suppression of Esophageal Cancer Tumorigenesis

Maoju Tang[1,3#], Miyuan Yang[2,3#], Jiling Wen[1], Xiao Liu[3], Lei Xu[4], Qiang Ma[1,3,4,*], Xiaowu Zhong[1,3,4,*] and Xiaolan Guo[1,3,4,*]

[1]Department of Clinical Laboratory, Affiliated Hospital of North Sichuan Medical College, Nanchong 637000, P.R. China; [2]Department of Clinical Laboratory, Beijing Anzhen Nanchong Hospital of Capital Medical University & Nanchong Central Hospital, Nanchong 637000, P.R. China; [3]School of Laboratory Medicine, North Sichuan Medical College, Nanchong 637000, P.R. China; [4]Translational Medicine Research Center, North Sichuan Medical College, Nanchong 637000, P.R. China

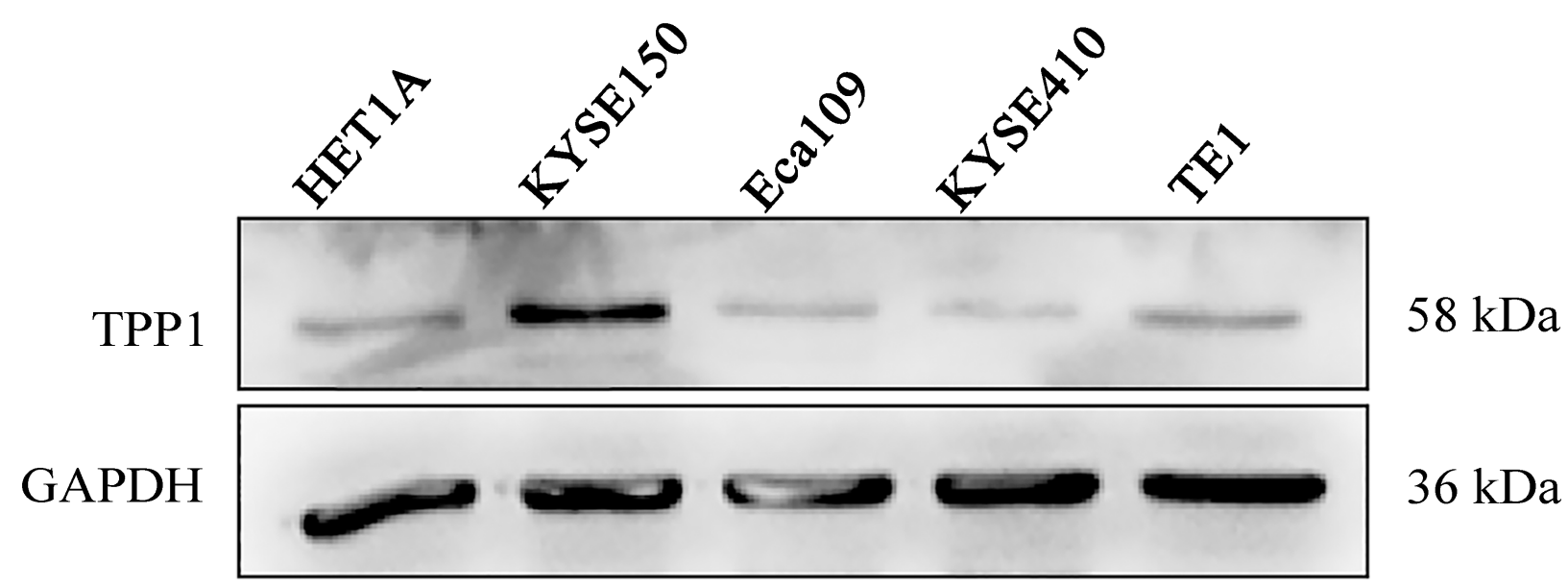

Western blot quantitative statistical analysis

Figure S1: Selection of cell lines for esophageal cancer.

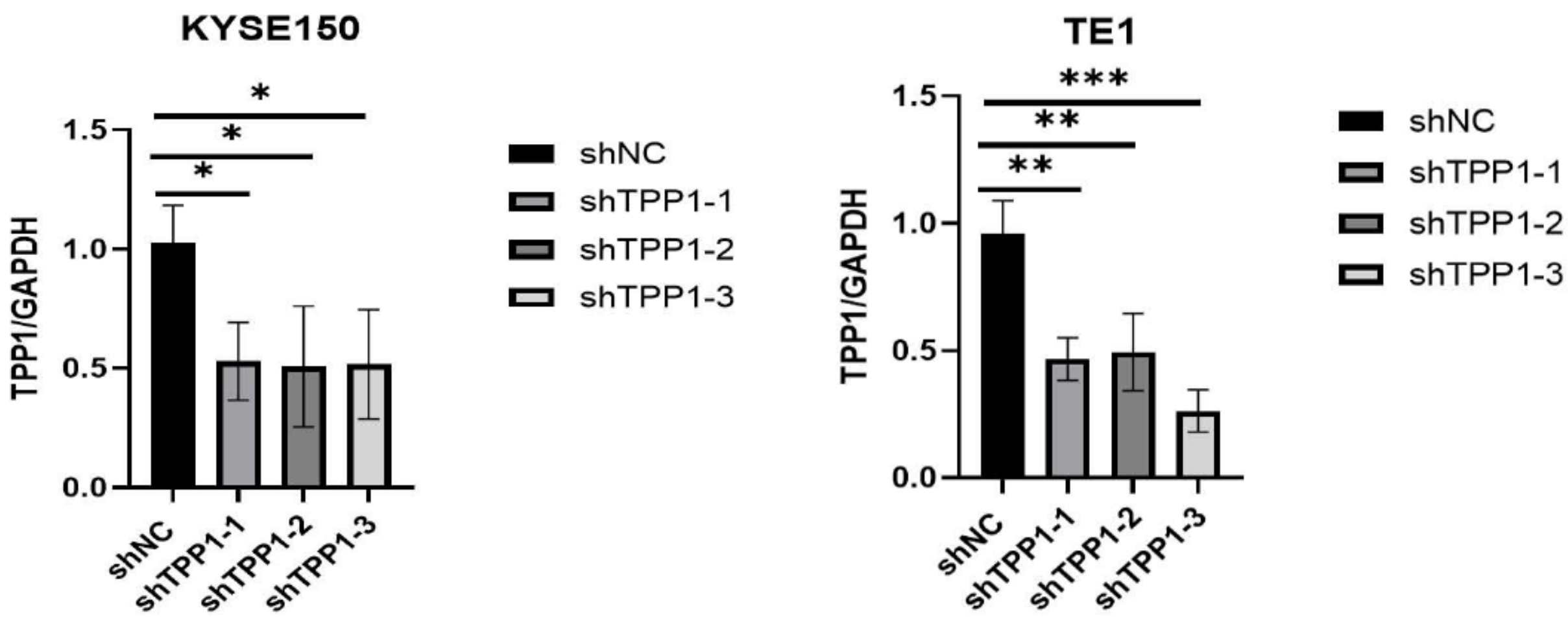

Figure S2: Quantitative analysis of TPP1 protein level after knockout of TPP1 protein (Figure 1E)

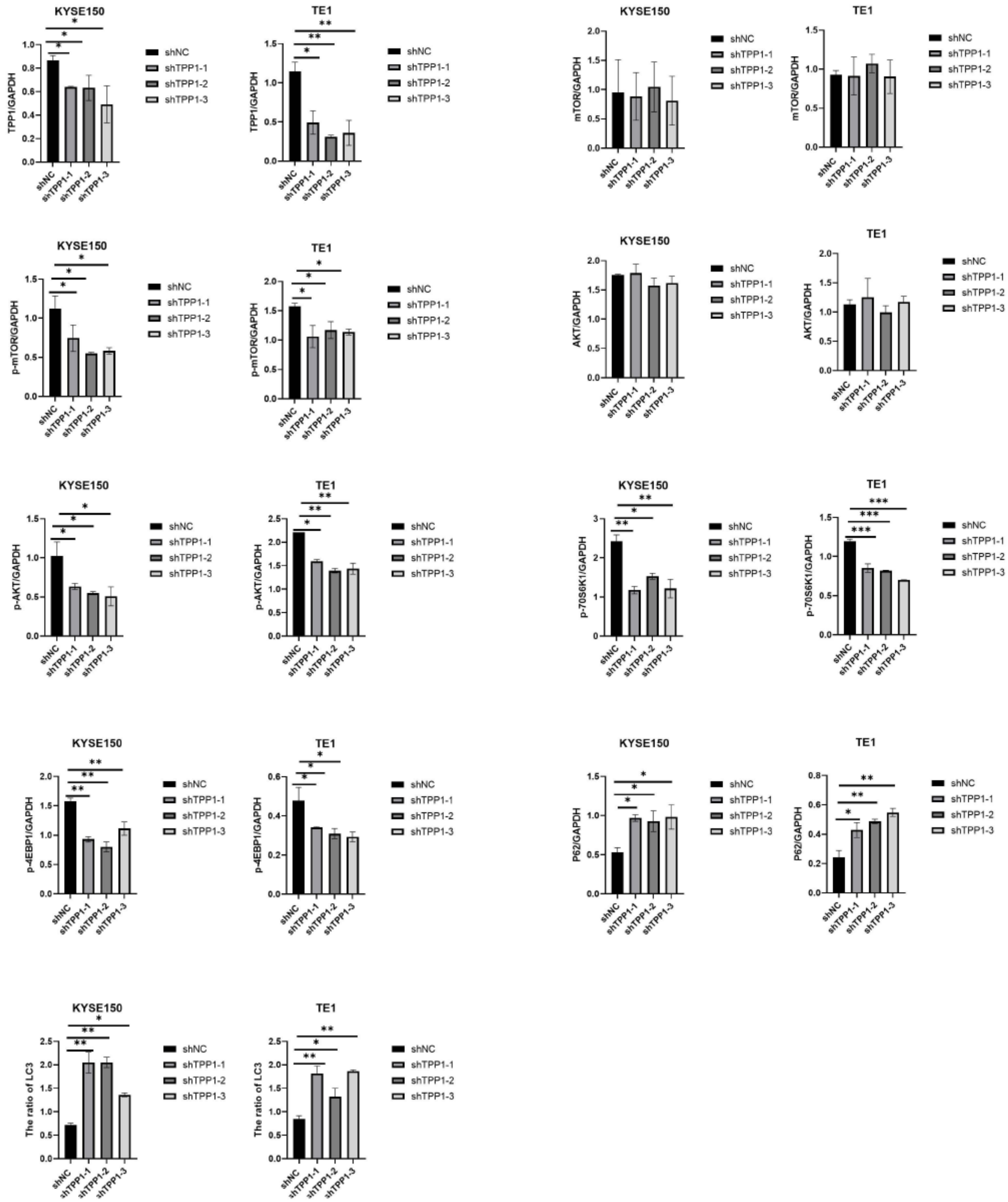

Figure S3: Quantitatively analyze the levels of AKT-mTOR signaling pathway and autophagy-related proteins after TPP1 knockdown (Figure 2D).

Note: in order from left to right and top to bottom, the proteins are: TPP1, mTOR, p-mTOR, AKT, p-AKT, p-70S6K1, p-4EBP1, P62, LC3.

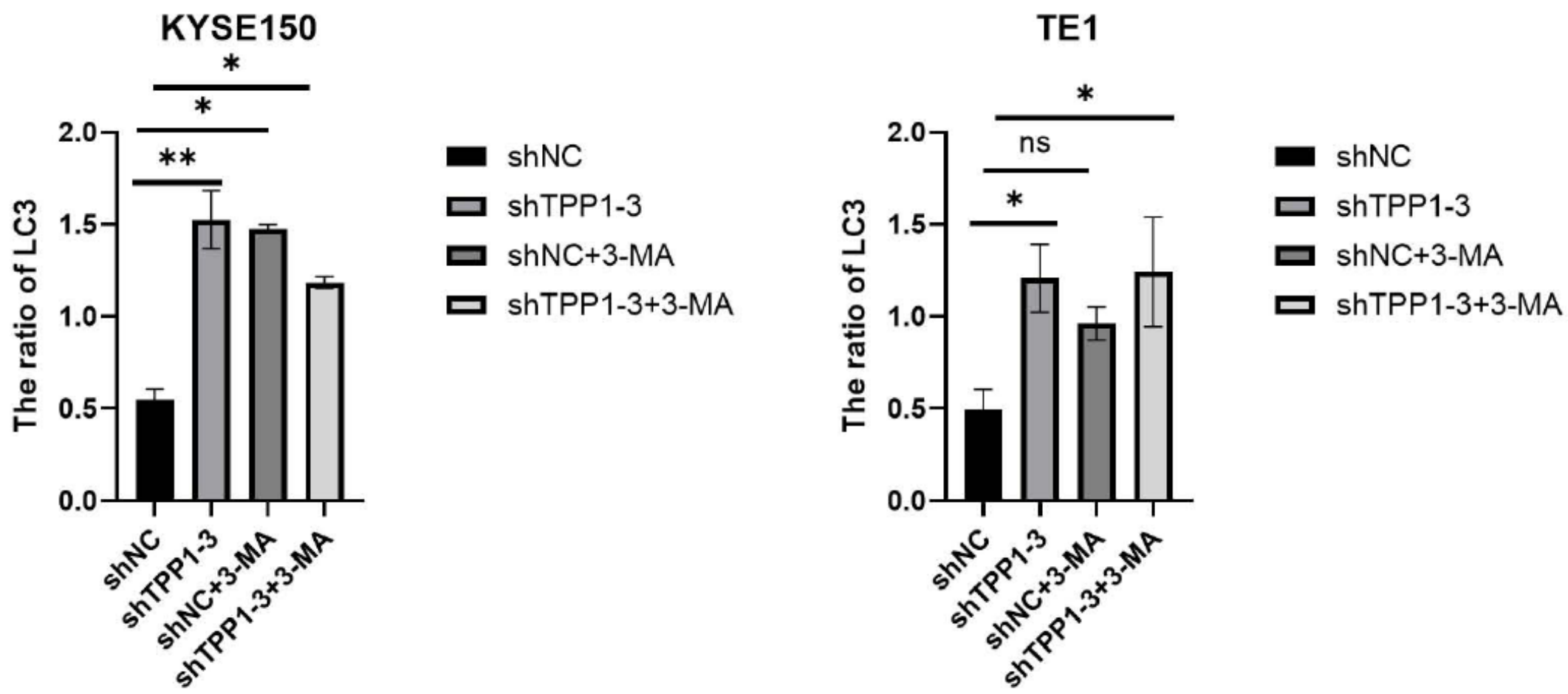

Figure S4: Quantitatively analyze the interference of 3-MA on the expression of autophagy molecule LC3 after TPP1 knocking down TPP1 (Figure 3A).

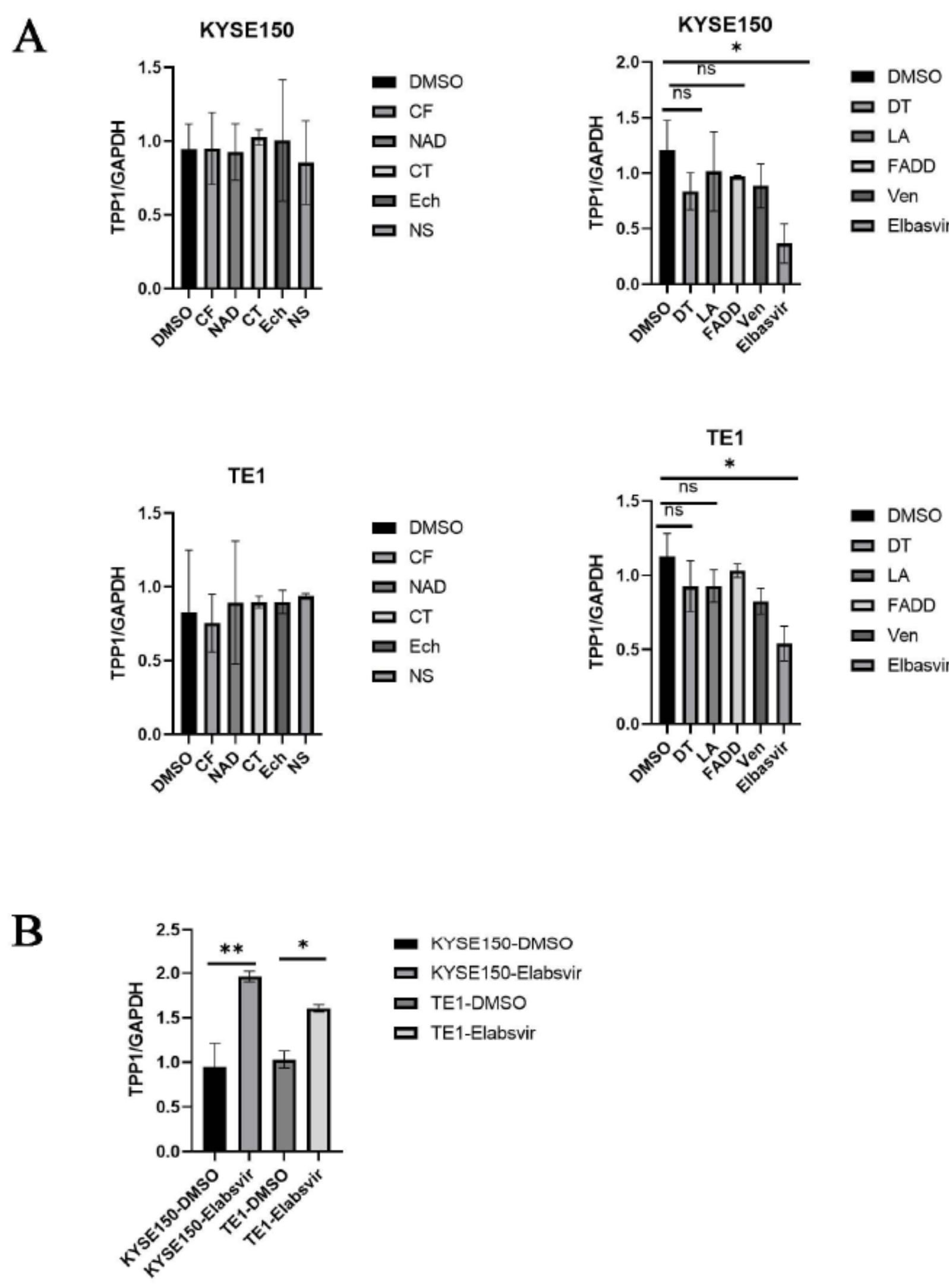

Figure S5: (A) Quantitatively analyze the expression of TPP1 protein after administration of 10 candidate targeted drugs (Figure 4A). (B) Quantitatively analyze the drug affinity reaction target stability test (Figure 4D).

Note: Diquafosol tetrasodium: DT; lithospemmic acid B:LA; Flavin Adenine Dinucleotide Disodium: FADD; Venetoclax:Ven; Ceftaroline fosamil: CF; cangrelor tetrasodiumm: CT; Echinacoside: Ech; Neomycin Sulphate B: NS ; NAD+ Elbasvir.

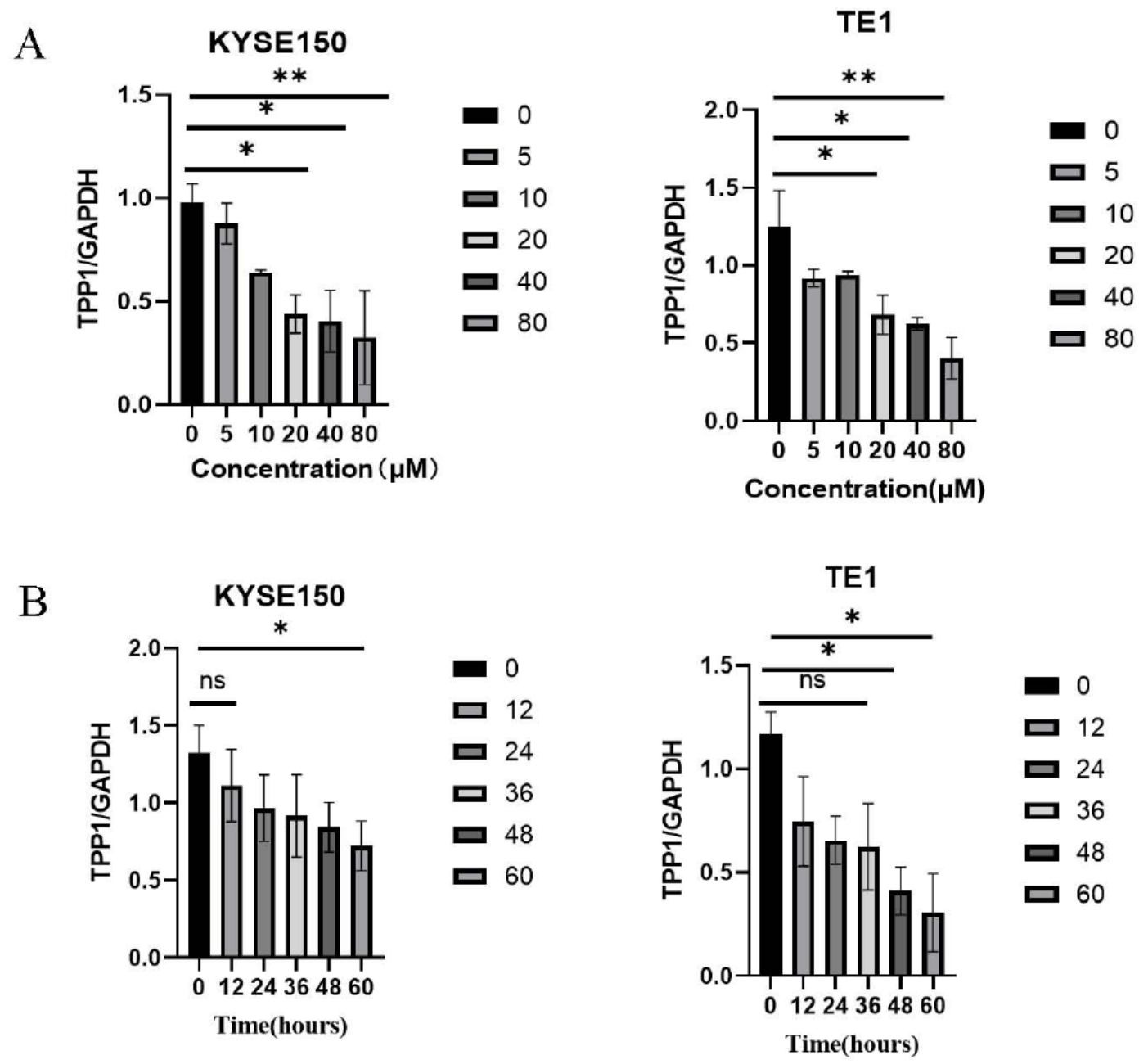

Figure S6: (A) Quantitatively analyze the expression of TPP1 protein after treatment of esophageal cancer with different concentrations of Elbasvir (Figure 5B). (B) Quantitatively analyze the expression of TPP1 protein in esophageal cancer cells treated with Elbasvir at different times(Figure 5C).

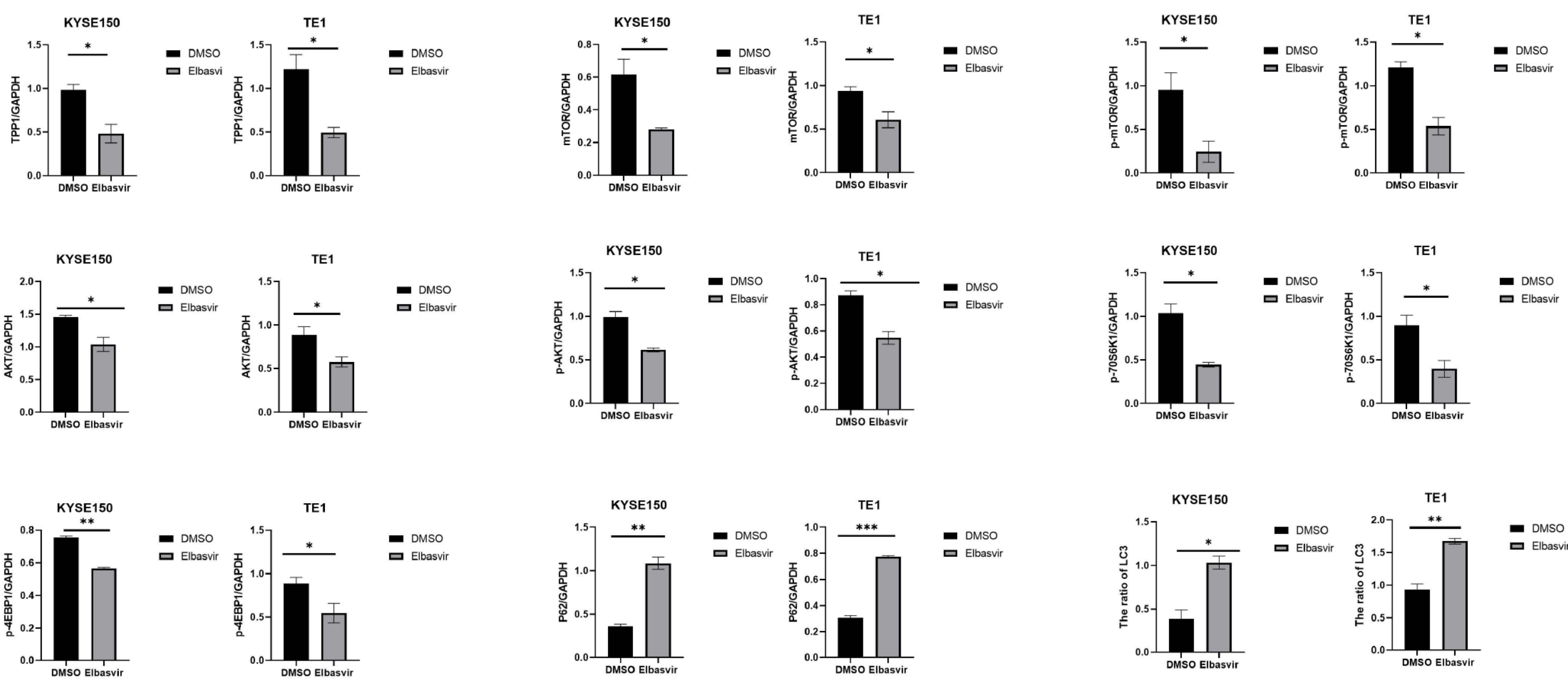

Figure S7: Quantitatively analyze the levels of AKT-mTOR signaling pathway and autophagy-related proteins 48 hours after Elbasvir treatment (Figure 6A).

Note: in order from left to right and top to bottom, the proteins are: TPP1, mTOR, p-mTOR, AKT, p-AKT, p-70S6K1, p-4EBP1, P62, LC3.

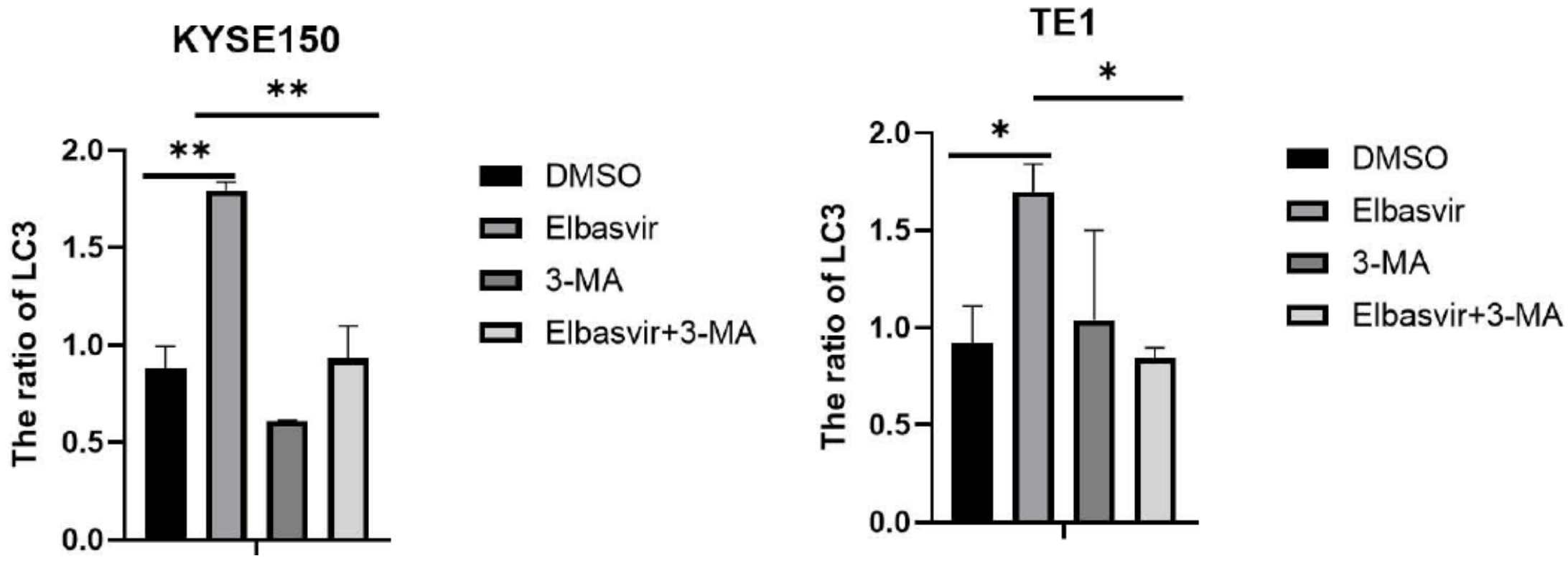

Figure S8: Quantitatively analyze the expression level of autophagy protein LC3 after 3-MA pretreatment with Elbasvir for 48 hours (Figure 6B).

Table1. Oligonucleotides sequences and primers

Names	Oilgonucleotides
**shRNAs**	
Human TPP1 shRNA1	ACATGTCATCCGAGGAAAG
Human TPP1 shRNA2	CTGAGAATGACCAGCTAAT
Human TPP1 shRNA3	AAGACTTAGATGTTCAGAA
**PCR primers**	
PCR-TPP1-F	5′-CAGAGTCCACCTCGTCCAAT-3′
PCR-TPP1-R	5′-CAGTGTCAGGCAGCTTTCAG -3′
PCR-β-actin-F	5′-GCAAGCAGGAGTATGACGAG-3′
PCR-β-actin-R	5′-CAA ATAAAGCCATGCCAATC-3′

Note: F：Forward primer；R：Reverse primer.

Table S2: Molecular inhibitors targeting TPP1 protein were screened using DOCK 6.9.

ID	Name	CAS	Grid_Score	Grid_vdw_energy	Grid_es_energy	Internal_energy_repulsive
T7423	Diquafosol tetrasodium	211427-08-6	-96.6653	-73.7993	-22.866	23.1336
T5725	lithospermic acid B/Salvianolic acid B	121521-90-2	-89.6658	-86.1516	-3.5142	29.6725
T5067	Flavin Adenine Dinucleotide Disodium	84366-81-4	-88.876	-77.7945	-11.0815	19.0504
T2119	ABT199（Venetoclax）	1257044-40-8	-88.5911	-81.1958	-7.3953	17.6538
T4544	Elbasvir	1370468-36-2	-86.0067	-84.9077	-1.099	39.5698
T7387	Ceftaroline fosamil	400827-46-5	-84.4566	-59.6563	-24.8003	10.0935
T1609	NAD+	53-84-9	-83.5418	-70.2805	-13.2613	10.3254
T5036	cangrelor tetrasodium	163706-36-3	-82.8493	-70.5056	-12.3437	17.3061
T1716	Echinacoside	82854-37-3	-82.1067	-75.0617	-7.0451	25.03
T2727	Salvianolic acid B/lithospermic acid B	115939-25-8	-82.0528	-76.4457	-5.607	15.0497
T2096	Neomycin Sulphate B	4146-30-9	-80.1315	-33.4595	-46.672	19.5487
T5024	Cefotiam Hexetil Hydrochloride	95789-30-3	-80.0314	-77.876	-2.1554	14.7417
T2971	Phytic acid	83-86-3	-79.9964	-34.5697	-45.4267	9.5364
T2813	Madecassoside	34540-22-2	-79.6991	-76.8917	-2.8074	43.9512
T5584	Sodium phytate hydrate	14306-25-3	-79.2969	-34.0268	-45.2701	10.409
T3934	Isavuconazonium sulfate	946075-13-4	-79.2202	-74.5237	-4.6965	37.9371
T4203	Ledipasvir acetone	1441674-54-9	-79.0815	-78.984	-0.0975	35.1951
T1776	Plerixafor	110078-46-1	-77.5387	-35.7098	-41.8289	4.4628
T1776L	Plerixafor 8HCl (AMD3100 8HCl)	155148-31-5	-77.3373	-35.5341	-41.8032	4.6131
T3334	Velpatasvir	1377049-84-7	-77.0124	-78.3448	1.3323	35.3045

T6200	Ledipasvir	1256388-51-8	-76.66	-74.4093	-2.2507	36.0038
T7903	Saquinavir	127779-20-8	-76.4807	-74.8646	-1.6161	20.4868
T1441	Capastat sulfate	1405-37-4	-76.2713	-52.8392	-23.4321	30.6625
T2605	R788 Disodium	1025687-58-4	-76.2094	-60.9958	-15.2136	10.1839
T4474	Asunaprevir	630420-16-5	-76.0854	-74.0022	-2.0833	34.209
T4509	Inositol nicotinate	6556-11-2	-76.0836	-74.3173	-1.7663	26.8726
T4686	Simeprevir	923604-59-5	-76.0447	-74.5442	-1.5005	20.6777
T0772	Troxerutin	7085-55-4	-75.9537	-72.11	-3.8437	19.9971
T0078	Lapatinib	231277-92-2	-75.8139	-72.834	-2.9799	9.5634
T0078L	Lapatinib ditosylate monohydrate	388082-78-8	-75.7808	-74.5582	-1.2226	10.0033
T0966	Sennoside A	81-27-6	-75.6939	-67.6322	-8.0617	44.1218
T6235	Lapatinib Ditosylate	388082-77-7	-75.6187	-71.3213	-4.2974	10.4697
T2217	Cephalomannine	71610-00-9	-75.4514	-72.8645	-2.5869	57.4648
T6229	Daclatasvir	1009119-64-5	-75.3824	-73.9898	-1.3926	23.397
T0313	Evans blue	314-13-6	-74.8522	-64.3416	-10.5106	40.8179
T1011	Itraconazole	84625-61-6	-74.515	-73.8433	-0.6717	21.0596
T3025	Asiaticoside	16830-15-2	-74.3485	-71.5051	-2.8433	25.4933
T3125	Amikacin Sulfate Salt	149022-22-0	-74.3299	-43.8021	-30.5278	9.5857
T8156	Saquinavir mesylate	149845-06-7	-74.3216	-72.4783	-1.8433	19.4826
T6211	Posaconazole	171228-49-2	-74.319	-73.2603	-1.0587	25.49
T8118	Bekanamycin	4696-76-8	-74.1382	-36.8094	-37.3288	8.5403
T1786	Daclatasvir dihydrochloride	1009119-65-6	-74.1301	-73.7739	-0.3562	37.2485
T1623	Lopinavir	192725-17-0	-74.1138	-71.8777	-2.2361	23.0977
T1670	Lanatoside C	17575-22-3	-73.9506	-71.7327	-2.2179	44.964
T0228	Methyl hesperidin	11013-97-1	-73.8692	-69.904	-3.9652	19.4788
T6115	Fostamatinib	901119-35-5	-73.8503	-61.2433	-12.6071	10.7895
T1104	Paromomycin Sulfate	1263-89-4	-73.7334	-49.8934	-23.84	50.2719
T2492	Perifosine	157716-52-4	-73.5204	-68.6365	-4.8839	13.2489
T1219	Sisomicin Sulfate	53179-09-2	-73.5073	-36.6939	-36.8134	10.9625
T0974	Novobiocin Sodium	1476-53-5	-73.166	-66.7413	-6.4247	22.5995
T1013	Amikacin	37517-28-5	-72.9678	-42.4355	-30.5323	20.0841
T1036	Netilmicin sulfate	56391-57-2	-72.8816	-42.8529	-30.0288	18.9317
T7943	Fluphenazine decanoate	5002-47-1	-72.7728	-73.275	0.5022	20.5101
T2734	Sennoside B	128-57-4	-72.6913	-67.0652	-5.6261	43.9521
T6019	Volasertib	755038-65-4	-72.5555	-54.7224	-17.8332	24.1866
T6577	Manidipine	89226-50-6	-72.3351	-67.6423	-4.6928	18.4127
T7766	Remdesivir	1809249-37-3	-72.2878	-69.1956	-3.0922	24.2323
T6736	Zafirlukast	107753-78-6	-72.2667	-68.0107	-4.256	27.6045
T6512	Geneticin sulfate	108321-42-2	-72.1915	-38.5479	-33.6436	15.84
T3523	Fostemsavir	864953-29-7	-72.0701	-59.3718	-12.6982	15.238
T2543	Cabazitaxel	183133-96-2	-72.0321	-71.7617	-0.2704	50.4527
T0950	Neomycin sulfate	1405-10-3	-72.0041	-38.5914	-33.4127	15.1759
T1791	LDK378	1032900-25-6	-71.9626	-67.6715	-4.2911	19.3712
T6267	Lomitapide	182431-12-5	-71.932	-70.5122	-1.4198	14.9446
T7213	Demecarium bromide	56-94-0	-71.8779	-65.7131	-6.1648	24.7954
T2767	Neohesperidin Dihydrochalcone	20702-77-6	-71.6658	-65.5188	-6.147	15.322
T1352	Adenosine 5'-triphosphate disodium salt	987-65-5	-71.6415	-45.372	-26.2695	4.6882
T3621	Brigatinib	1197953-54-0	-71.3861	-48.7566	-22.6295	19.7991

T0193	Manidipine dihydrochloride	89226-75-5	-71.3799	-67.9352	-3.4447	25.8967
T8397	Ixazomib citrate	1239908-20-3	-71.2403	-63.6782	-7.5621	17.7438
T1677	Montelukast	158966-92-8	-71.0593	-66.531	-4.5283	24.8982
T3762	Hyaluronic acid	9004-61-9	-71.001	-66.4633	-4.5377	24.046
T1616	Cefotiam hydrochloride	66309-69-1	-70.9952	-64.4253	-6.5699	7.6599
T2400	Candesartan Cilexetil	145040-37-5	-70.977	-66.4285	-4.5484	19.4433
T6549	Isepamicin sulfate	67814-76-0	-70.9437	-40.6266	-30.3172	19.5583
T0968	Paclitaxel	33069-62-4	-70.9264	-70.5237	-0.4027	77.0978
T5031	Elagolix sodium	832720-36-2	-70.8898	-63.5874	-7.3024	45.5217
T0420	Pentetic Acid	67-43-6	-70.8025	-45.0726	-25.7299	11.3035
T1677L	Montelukast sodium	151767-02-1	-70.7295	-65.6134	-5.1161	25.5552
T5028	Landiolol hydrochloride	144481-98-1	-70.6508	-67.3725	-3.2783	6.7808
T2713	D-Stachyose tetrahydrate	10094-58-3	-70.4951	-64.6691	-5.826	15.9351
T1518	Olmesartan Medoxomil	144689-63-4	-70.4717	-64.5902	-5.8815	31.4923
T2741	Glycyrrhizin	1405-86-3	-70.3931	-50.3528	-20.0402	24.7251
T0861	Tylosin tartrate	74610-55-2	-70.3636	-68.7934	-1.5702	50.771
T6874	Lercanidipine hydrochloride	132866-11-6	-70.3517	-67.0337	-3.3181	27.8733
T1255	Tobramycin	32986-56-4	-70.3353	-33.3858	-36.9495	13.3703
T1326	Gentamycin sulfate	1405-41-0	-70.2956	-36.3983	-33.8973	16.1655
T4168	Visomitin	934826-68-3	-70.1978	-70.4365	0.2388	17.2511
T5161	Gadodiamide	131410-48-5	-70.1807	-44.6356	-25.5451	9.2241
T2519	Lercanidipine	100427-26-7	-70.0622	-70.1556	0.0934	25.3353
T2872	Ginsenoside Re	52286-59-6	-69.8033	-67.9761	-1.8272	46.1068
T0138	Cefpiramide acid	70797-11-4	-69.7413	-62.8448	-6.8964	15.912
T7392	Revefenacin	864750-70-9	-69.7202	-67.7574	-1.9628	23.6315
T2737	Neohesperidin	13241-33-3	-69.6053	-65.7949	-3.8105	14.8487
T6230	Imatinib	152459-95-5	-69.4629	-51.7575	-17.7053	8.3444
T1489	Fosinopril sodium	88889-14-9	-69.3806	-62.8367	-6.5439	36.2553
T0793	Kanamycin sulfate	25389-94-0	-69.2756	-34.4618	-34.8138	8.8262
T4409	Gilteritinib	1254053-43-4	-69.2638	-62.8144	-6.4493	19.2522
T6431	Calcium Levofolinate	80433-71-2	-69.1836	-60.6171	-8.5665	11.6286
T3977	Lifitegrast	1025967-78-5	-69.0232	-65.2825	-3.7407	14.2713
T6487	Encorafenib	1269440-17-6	-69.0231	-67.3587	-1.6645	15.3337
T8393	UTP, Trisodium Salt	19817-92-6	-68.988	-40.5975	-28.3905	7.4911
T2761	Naringin dihydrochalcone	18916-17-1	-68.8991	-63.2561	-5.6431	15.0163
T1084	Apramycin sulfate	65710-07-8	-68.7633	-40.966	-27.7972	10.6424
T1474	Otilonium bromide	26095-59-0	-68.7575	-65.7655	-2.9919	12.1304
T1520	Aliskiren hemifumarate	173334-58-2	-68.7492	-55.9532	-12.7959	25.2784
T6595	Moexipril HCl	82586-52-5	-68.6005	-65.3796	-3.2209	31.9564
T6437	Cefoperazone	62893-19-0	-68.5517	-66.4258	-2.1259	32.8743
T1485	Methotrexate	59-05-2	-68.4847	-58.2201	-10.2646	14.9251
T0148	Calcium folinate	1492-18-8	-68.4621	-59.3574	-9.1047	20.7079
T7417	Avatrombopag	570406-98-3	-68.4515	-61.3748	-7.0767	15.7191
T1073	Dronedarone hydrochloride	141625-93-6	-68.4197	-66.3621	-2.0577	11.1717
T6044	Telotristat Etiprate	1137608-69-5	-68.3921	-70.9427	2.5506	16.9233
T0121	Azelnidipine	123524-52-7	-68.3426	-61.3346	-7.008	20.3288
T0522	Diammonium Glycyrrhizinate	79165-06-3	-68.2846	-65.2552	-3.0294	19.9312
T8301	Amaranth	915-67-3	-68.053	-57.2374	-10.8156	19.8637

T5134	Vilanterol trifenatate	503070-58-4	-68.0422	-63.5778	-4.4643	12.4085
T1228	Cefonicid sodium	61270-78-8	-68.0242	-63.1583	-4.8659	11.4537
T0148L	Folinic Acid Calcium Salt Pentahydrate	6035-45-6	-67.9286	-59.1018	-8.8268	13.6642
T5863	Indinavir sulfate	157810-81-6	-67.9148	-64.3447	-3.57	16.0518
T2409L	Tenofovir Disoproxil Fumarate	202138-50-9	-67.8818	-68.6159	0.7341	14.6266
T1218	Cefoperazone sodium	62893-20-3	-67.8547	-63.0074	-4.8473	26.4204
T6813	Cyclo(RGDyK)	250612-42-1	-67.8412	-56.7444	-11.0968	17.2122
T2911	Stevioside	57817-89-7	-67.8217	-67.0791	-0.7426	30.2147
T0795	Rutin	153-18-4	-67.7254	-65.3148	-2.4107	14.4008
T0328	Hexetidine	141-94-6	-67.7152	-38.8371	-28.8781	10.5342
T1465	Mupirocin	12650-69-0	-67.7066	-55.5609	-12.1456	17.005
T4506	Gadopentetate dimeglumine	86050-77-3	-67.6161	-42.7669	-24.8492	5.6967
T1000L	Chlorhexidine Acetate	56-95-1	-67.6031	-60.4736	-7.1296	10.3281
T6001	Nelfinavir Mesylate	159989-65-8	-67.5602	-64.6765	-2.8837	22.4658
T6226	Pemetrexed Disodium Hydrate	357166-30-4	-67.5515	-54.7765	-12.775	11.1286
T0062	Folic acid	59-30-3	-67.5426	-56.4016	-11.141	8.3543
T6025	Danoprevir	850876-88-9	-67.5263	-56.8945	-10.6318	18.0674
T6219	Azilsartan Medoxomil	863031-21-4	-67.3597	-67.4327	0.073	14.112
T0158	Mitoxantrone hydrochloride	70476-82-3	-67.3375	-54.5561	-12.7813	33.9695
T0878	Diosmin	520-27-4	-67.2784	-65.5445	-1.7339	14.3129
T8148	Mupirocin calcium	115074-43-6	-67.2783	-65.8677	-1.4106	19.8628
T2372	Ponatinib	943319-70-8	-67.2126	-47.7651	-19.4474	10.5202
T0595	Naringin	10236-47-2	-67.168	-62.577	-4.591	13.2069
T8126	Cefodizime Sodium	86329-79-5	-67.1204	-59.0005	-8.1199	10.6499
T2891	Monoammonium glycyrrhizinate	53956-04-0	-67.0995	-65.2461	-1.8534	19.9333
T1621	Imatinib Mesylate	220127-57-1	-67.0983	-62.2463	-4.852	4.3012
T1000	Chlorhexidine	55-56-1	-67.0307	-60.5882	-6.4425	10.7073
T0033	Miltefosine	58066-85-6	-67.0213	-61.3265	-5.6948	11.1235
T6438	Cefoselis Sulfate	122841-12-7	-66.9618	-61.0746	-5.8872	18.9919
T2409	Tenofovir alafenamide	379270-37-8	-66.9584	-65.1818	-1.7765	8.2105
T1470	Fexofenadine hydrochloride	153439-40-8	-66.9308	-64.8269	-2.1039	20.7586
T7043	Cefotetan	69712-56-7	-66.9122	-55.3612	-11.551	10.3539
T1147	Chlorhexidine hydrochloride	3697-42-5	-66.855	-60.645	-6.21	10.2917
T0752	L-Ascorbyl 6-palmitate	137-66-6	-66.8316	-61.7329	-5.0987	11.1249
T1470L	Fexofenadine hydrochloride	138452-21-8	-66.8176	-65.2707	-1.5469	21.6136
T2016	MLN9708	1201902-80-8	-66.816	-60.4996	-6.3164	13.9736
T4547	Grazoprevir	1350514-68-9	-66.7908	-56.1807	-10.6101	22.94
T2961	Notoginsenoside R1	80418-24-2	-66.6846	-65.0348	-1.6498	40.1923
T1035	Hesperidin	520-26-3	-66.6026	-64.7349	-1.8677	16.4432
T8239	Cromoglicic acid	16110-51-3	-66.5737	-60.2442	-6.3295	14.5694
T6190	Telaprevir	402957-28-2	-66.4973	-64.8954	-1.6019	27.0718
T4977	Sofalcone	64506-49-6	-66.4512	-57.2149	-9.2363	12.0304
T7604	Valrubicin	56124-62-0	-66.4206	-63.4837	-2.9369	38.6534
T7593	Levomefolate calcium	151533-22-1	-66.3856	-56.6334	-9.7522	5.8042
T0189	Pemetrexed acid	137281-23-3	-66.3842	-53.9393	-12.4448	11.5586
T1260	Sodium cromoglycate	15826-37-6	-66.3605	-59.4344	-6.9261	13.8619
T1995	Fedratinib	936091-26-8	-66.3035	-64.5332	-1.7703	15.9057
T5001	Nintedanib Ethanesulfonate Salt	656247-18-6	-66.2523	-62.4494	-3.8029	20.3879

T1777	Nintedanib	656247-17-5	-66.1989	-62.5274	-3.6715	20.6307
T8183	Deslanoside	17598-65-1	-66.1799	-62.7624	-3.4175	32.4712
T2494	Cilengitide	188968-51-6	-66.1434	-55.0206	-11.1228	14.961
T0136	Citicoline	987-78-0	-66.054	-56.4346	-9.6194	9.0916
T11897	Telotristat ethyl	1033805-22-9	-66.0079	-62.8928	-3.1151	24.1034
T7056	Dronedarone	141626-36-0	-65.9931	-65.8989	-0.0942	11.0979
T0186	Docetaxel trihydrate	148408-66-6	-65.9852	-65.1975	-0.7878	70.8303
T17199	Udenafil	268203-93-6	-65.9741	-57.7555	-8.2186	19.7779
T1790	Fosaprepitant dimeglumine	265121-04-8	-65.9588	-54.826	-11.1328	16.048
T0189L	Pemetrexed disodium	150399-23-8	-65.9573	-49.0175	-16.9397	15.124
T7111	Tenofovir Disoproxil	201341-05-1	-65.8822	-63.6474	-2.2348	11.7214
T2066	Quizartinib	950769-58-1	-65.8239	-56.7111	-9.1128	10.0886
T1214	Ribostamycin sulfate	53797-35-6	-65.8203	-26.6164	-39.2039	21.9097
T6403	Siponimod	1230487-00-9	-65.8017	-64.5695	-1.2322	17.4833
T2609	Masitinib	790299-79-5	-65.7822	-50.415	-15.3671	4.3614
T3216	Selexipag	475086-01-2	-65.6594	-60.6121	-5.0473	16.8471
T1034	Docetaxel	114977-28-5	-65.5969	-64.6589	-0.9381	69.96
T2490	Osimertinib	1421373-65-0	-65.4731	-62.4745	-2.9986	8.7702
T6653	Salmeterol Xinafoate	94749-08-3	-65.441	-61.2983	-4.1427	10.2488
T1208	Citicoline sodium	33818-15-4	-65.4195	-55.4186	-10.0009	10.9559
T1020	Doxorubicin hydrochloride	25316-40-9	-65.3186	-59.5667	-5.7519	30.4905
T3226	Paritaprevir	1221573-85-8	-65.2845	-65.8805	0.596	22.4449
T3116	Atorvastatin Calcium	134523-03-8	-65.2232	-59.8071	-5.4161	22.5557
T2323	Efonidipine	111011-63-3	-65.1433	-64.8267	-0.3166	22.4013
T2561	Macitentan	441798-33-0	-65.11	-64.1907	-0.9193	14.1271
T2213	Terconazole	67915-31-5	-65.0373	-64.4366	-0.6007	22.7663
T1634	Glyburide	10238-21-8	-65.0172	-58.5292	-6.488	7.0898
T6632	Raltitrexed	112887-68-0	-64.9219	-56.5945	-8.3275	12.7014
T3054	Daurisoline	70553-76-3	-64.8678	-62.2499	-2.618	18.9252
T1405	Cefditoren pivoxil	117467-28-4	-64.8458	-63.1679	-1.6779	13.4444
T0371	Gliquidone	33342-05-1	-64.8382	-54.7395	-10.0987	17.6619
T0152	Bosutinib	380843-75-4	-64.7946	-47.1863	-17.6082	11.7453
T4998	Cefpodoxime proxetil	87239-81-4	-64.7769	-63.0486	-1.7283	14.6116
T5022	Ceforanide	60925-61-3	-64.7088	-53.7029	-11.0059	8.1576
T1129	Benfotiamine	22457-89-2	-64.6945	-53.4317	-11.2628	15.065
T3112	Verteporfin	129497-78-5	-64.6681	-58.9411	-5.727	14.849
T6322	Copanlisib	1032568-63-0	-64.6211	-48.5242	-16.0969	9.0673
T8003	Methotrexate disodium	7413-34-5	-64.6153	-55.2589	-9.3564	8.6796
T2325	Neratinib	698387-09-6	-64.6102	-53.4522	-11.158	12.2351
T2335	Ebastine	90729-43-4	-64.5927	-62.5805	-2.0121	16.3359
T2381	Abemaciclib	1231929-97-7	-64.5647	-48.2481	-16.3166	13.7295
T4991	Ertapenem sodium	153773-82-1	-64.4966	-52.7059	-11.7908	10.6016
T4978	Sarpogrelate hydrochloride	135159-51-2	-64.4729	-54.4479	-10.025	14.9807
T2146	Fulvestrant	129453-61-8	-64.4708	-62.3327	-2.138	12.2976
T7346	Lodenafil	139755-85-4	-64.4199	-55.9553	-8.4646	9.3545
TQ0277	Pralsetinib	2097132-94-8	-64.3741	-63.7005	-0.6736	17.0883
T6917	Oleuropein	32619-42-4	-64.369	-62.5372	-1.8318	17.5743
T3678	Entrectinib	1108743-60-7	-64.3685	-63.1482	-1.2203	14.7368

T1523	Teniposide	29767-20-2	-64.2866	-61.945	-2.3416	11.8686
T5856	Barnidipine	104757-53-1	-64.2827	-60.0788	-4.2039	17.5107
T5964	Olmesartan	144689-24-7	-64.1921	-58.3813	-5.8108	15.8174
T2147	Nilotinib (monohydrochloride monohydrate)	923288-90-8	-64.1761	-63.7266	-0.4495	8.3114
T2328	Radotinib	926037-48-1	-64.1016	-64.6041	0.5025	7.453
T2324	Darunavir	206361-99-1	-64.0742	-63.1602	-0.9141	14.3233
T6264	Bosentan	147536-97-8	-64.0462	-64.1224	0.0762	17.4175
T1053	Mebeverine hydrochloride	2753-45-9	-64.0415	-61.3445	-2.697	14.8747
T1151	Roxithromycin	80214-83-1	-64.0211	-59.2474	-4.7737	51.5354
T3263	Cefminox Sodium	92636-39-0	-63.9845	-54.7295	-9.255	10.1198
T0060	Streptomycin sulfate	3810-74-0	-63.9756	-49.378	-14.5976	22.0691
TMS0418	Dihydroergotoxine mesylate	8067-24-1	-63.9413	-61.5761	-2.3652	15.472
T1223	Ceftriaxone sodium	104376-79-6	-63.9192	-51.1445	-12.7747	12.1136
T6179	Latamoxef sodium	64953-12-4	-63.8831	-53.8709	-10.0123	17.7307
T4990	Cefcapene Pivoxil Hydrochloride	147816-24-8	-63.8266	-62.3192	-1.5074	15.7044
T2369	Rociletinib	1374640-70-6	-63.7668	-64.5772	0.8104	18.1293
T1254	Chlorophyllin	11006-34-1	-63.7379	-43.6613	-20.0766	21.3235
T1088	Repaglinide	135062-02-1	-63.6511	-53.3274	-10.3237	16.5579
T3S1858	Lutein	127-40-2	-63.6491	-62.6244	-1.0248	20.6855
T1511	Daunorubicin hydrochloride	23541-50-6	-63.6375	-59.6173	-4.0202	35.5505
T8401	Efonidipine hydrochloride monoethanolate	111011-76-8	-63.4411	-61.8048	-1.6363	24.7867
T0191	Linagliptin	668270-12-0	-63.4189	-63.5006	0.0817	10.1282
T3335	Darunavir?Ethanolate	635728-49-3	-63.4082	-61.0238	-2.3844	24.2894
T0694	Pranlukast	103177-37-3	-63.3975	-58.5575	-4.84	8.7046
T1675	Adefovir dipivoxil	142340-99-6	-63.3896	-62.7357	-0.6538	12.6156
T7779	Nelfinavir	159989-64-7	-63.3798	-59.2053	-4.1744	16.6251
T5666	Dipotassium glycyrrhizinate	68797-35-3	-63.3706	-50.6027	-12.7678	22.6457
T1549	Raloxifene hydrochloride	82640-04-8	-63.3055	-63.911	0.6055	9.7084
T0969	Doramectin	117704-25-3	-63.2575	-62.2501	-1.0074	37.2837
T7282	Tenofovir alafenamide fumarate	1392275-56-7	-63.1804	-62.9401	-0.2403	9.2512
T1504	Silodosin	160970-54-7	-63.149	-58.9317	-4.2173	11.5119
T0827	Cefsulodine sodium	52152-93-9	-63.1484	-50.0591	-13.0893	16.5259
T1524	Nilotinib	641571-10-0	-63.0698	-62.2395	-0.8303	8.3029
T3634	Osimertinib mesylate	1421373-66-1	-63.0356	-58.361	-4.6746	11.6594
T1569	Tigecycline	220620-09-7	-63.0068	-53.7722	-9.2345	28.3983
T0805	Quinapril hydrochloride	82586-55-8	-62.9578	-59.4389	-3.5189	24.6585
T2334	Avanafil	330784-47-9	-62.9371	-61.8929	-1.0442	9.2591
T3093	Spermine?Tetrahydrochloride	306-67-2	-62.9277	-18.93	-43.9977	3.5667
T2537	Tirofiban hydrochloride monohydrate	150915-40-5	-62.9124	-54.1667	-8.7457	8.4268
T6120	Pralatrexate	146464-95-1	-62.726	-53.9857	-8.7404	12.2189
T0155	Argatroban	74863-84-6	-62.7235	-60.9632	-1.7603	19.9791
T5841	Travoprost	157283-68-6	-62.609	-60.2374	-2.3716	15.3745
T6295	Dabigatran	211914-51-1	-62.5891	-52.2014	-10.3877	14.899
T3111	LY2835219	1231930-82-7	-62.5587	-55.8086	-6.7501	18.4728
T0081	Lafutidine	118288-08-7	-62.5349	-64.5625	2.0275	11.4668
T6S1966	Curculigoside	85643-19-2	-62.4744	-58.8264	-3.648	9.436
T1215	Nicardipine hydrochloride	54527-84-3	-62.4732	-60.6246	-1.8486	11.6953
T2528	Latanoprost	130209-82-4	-62.3916	-61.0588	-1.3329	15.2909

T1475	Fesoterodine fumarate	286930-03-8	-62.3758	-60.5747	-1.801	35.2904
T1461	Candesartan	139481-59-7	-62.3731	-56.0384	-6.3347	16.1492
T4097	Vardenafil hydrochloride trihydrate	330808-88-3	-62.231	-58.1439	-4.087	13.4736
T0198	Sodium ceftiofur	104010-37-9	-62.2025	-56.3526	-5.8499	9.1509
T1788	EPZ6438	1403254-99-8	-62.1846	-61.1513	-1.0333	18.369
T0271	Iodipamide	606-17-7	-62.1697	-52.4259	-9.7438	13.5489
T2322	Fimasartan	247257-48-3	-62.1623	-57.0967	-5.0656	24.7646
T1227	Cepazine	64544-07-6	-62.1575	-60.3483	-1.8091	19.6256
T4202	Delamanid	681492-22-8	-62.1571	-59.2573	-2.8998	7.3031
T0388	Cilnidipine	132203-70-4	-62.1463	-61.06	-1.0863	14.1649
T2535	Ivabradine hydrochloride	148849-67-6	-62.1375	-60.3462	-1.7913	10.5402
T5164	Cabozantinib hydrochloride (849217-68-1(free base))	T5164	-62.0805	-63.8532	1.7727	11.4073
T1057	Azilsartan	147403-03-0	-61.9898	-56.5355	-5.4543	12.5126
T4200	AHU377 calcium salt	1369773-39-6	-61.9453	-58.796	-3.1493	15.8005
T1029	Aztreonam	78110-38-0	-61.9364	-57.5803	-4.3561	10.2788
T5515	Hemin	16009-13-5	-61.7913	-35.6953	-26.0961	12.8452
T1331	Flavin mononucleotide	130-40-5	-61.7374	-57.1949	-4.5426	14.8361
T6388	Amprenavir	161814-49-9	-61.7168	-59.4729	-2.244	16.802
T1448	Dasatinib	302962-49-8	-61.714	-55.151	-6.563	14.6834
T6227	Benidipine hydrochloride	91599-74-5	-61.7001	-57.6007	-4.0995	15.9804
T1570	Telmisartan	144701-48-4	-61.629	-58.3798	-3.2492	14.3874
T6676	Sofosbuvir	1190307-88-0	-61.596	-60.7268	-0.8692	19.6715
T0467	Sildenafil citrate	171599-83-0	-61.5647	-59.3291	-2.2355	15.1659
T3283	Cetilistat	282526-98-1	-61.5612	-60.9143	-0.6469	7.8892
T5126	Glecaprevir	1365970-03-1	-61.4833	-51.6714	-9.8118	18.1495
T3726	Erdafitinib	1346242-81-6	-61.4265	-60.3313	-1.0952	9.3277
T0132	Etoposide	33419-42-0	-61.4082	-57.9482	-3.46	11.9654
T3170	Troglitazone	97322-87-7	-61.3973	-56.8561	-4.5412	10.4459
T1190	Cefmenoxime hydrochloride	75738-58-8	-61.388	-56.3294	-5.0586	10.4736
T3380	Homoharringtonine	26833-87-4	-61.3498	-57.9386	-3.4112	20.5775
T0179	Ticagrelor	274693-27-5	-61.3383	-61.0431	-0.2952	7.8501
T6268	Ceftiofur hydrochloride	103980-44-5	-61.3054	-53.5278	-7.7776	9.5542
T1723	ADP	58-64-0	-61.2558	-41.687	-19.5688	4.2704
T2509	Tozasertib	639089-54-6	-61.2553	-58.8065	-2.4489	5.7862
T4988	Boceprevir	394730-60-0	-61.2525	-58.0439	-3.2086	23.6666
T0679	Ketoconazole	65277-42-1	-61.2316	-61.0026	-0.229	23.5051
T1797	Cabozantinib Malate	1140909-48-3	-61.2315	-62.1839	0.9524	10.4644
T6983	Sildenafil Mesylate	1308285-21-3	-61.1734	-58.9788	-2.1947	13.2424
T6633	Ranolazine	95635-55-5	-61.0664	-42.8427	-18.2237	7.6368
T2586	Cabozantinib	849217-68-1	-61.0072	-61.2646	0.2575	12.5273
T0932	Cefixime	79350-37-1	-60.9861	-55.696	-5.2901	6.5629
T4995	Cefpirome sulfate	98753-19-6	-60.9638	-55.8292	-5.1346	13.3286
T1671	Mirabegron	223673-61-8	-60.9585	-50.4391	-10.5194	6.2359
T7120	Cefetamet pivoxil hydrochloride	111696-23-2	-60.878	-58.9303	-1.9478	13.354
T6628	Pirarubicin	72496-41-4	-60.8701	-52.3111	-8.5591	53.7455
T6597	Mosapride Citrate	112885-42-4	-60.8563	-57.9118	-2.9444	13.3107
T2538	Bimatoprost	155206-00-1	-60.8512	-59.1323	-1.7189	10.0116
T1075	Vitamin K1	84-80-0	-60.8473	-61.6039	0.7567	19.0364

T4874	Thiamine pyrophosphate	154-87-0	-60.7894	-44.8575	-15.9319	9.0805
T1603	Glipizide	29094-61-9	-60.6906	-51.6889	-9.0016	7.4064
T0953	Cefazolin sodium salt	27164-46-1	-60.6721	-53.9975	-6.6746	10.7987
T2303	Afatinib	439081-18-2	-60.6676	-56.3705	-4.2972	10.0372
T2555	Aclidinium bromide	320345-99-1	-60.4711	-58.4987	-1.9724	13.9962
T6544	Inulin	9005-80-5	-60.4689	-55.5641	-4.9048	19.0136
T0247	Acarbose	56180-94-0	-60.4645	-56.7908	-3.6737	25.9097
T3913	Saikosaponin D	20874-52-6	-60.4244	-57.7908	-2.6336	18.8644
T2732	(-)-Epicatechin gallate	1257-08-5	-60.4179	-56.0091	-4.4088	8.4654
T8405	Vardenafil hydrochloride	224785-91-5	-60.4094	-61.2957	0.8863	21.2587
T2211	Dihydrostreptomycin sulfate	5490-27-7	-60.3767	-52.1056	-8.2711	18.0604
T6476	Dofetilide	115256-11-6	-60.3698	-54.776	-5.5937	12.4998
T1291	Cisapride	81098-60-4	-60.3377	-59.5552	-0.7825	7.6254
T0125	Epirubicin hydrochloride	56390-09-1	-60.2907	-49.8354	-10.4553	28.9692
T4451	Estramustine phosphate sodium	52205-73-9	-60.2151	-57.5859	-2.6292	14.1254
T2789	Scutellarin	27740-01-8	-60.1991	-55.2829	-4.9162	11.647
T1448L	Dasatinib monohydrate	863127-77-9	-60.1783	-56.3542	-3.8241	7.6131
T0127	Glimepiride	93479-97-1	-60.1582	-55.576	-4.5821	13.9571
T2S0257	Dehydroandrographolide succinate	786593-06-4	-60.1387	-53.1756	-6.963	16.6478
T2531	Eprosartan Mesylate	144143-96-4	-60.1221	-51.0222	-9.0999	14.7764
T6272	Fosbretabulin Disodium	168555-66-6	-60.1217	-46.7752	-13.3465	11.2216
T4749	Squalene	111-02-4	-60.1195	-60.4929	0.3734	18.0807
T0474	Erythromycin ethylsuccinate	1264-62-6	-60.116	-61.0792	0.9631	64.249
T2923	Apremilast	608141-41-9	-59.9996	-60.452	0.4524	16.9848
T8270	Deserpidine	131-01-1	-59.9946	-62.1903	2.1957	13.5102
T8062	Cefazedone	56187-47-4	-59.9524	-53.6179	-6.3345	10.5249
T6675	Sodium Picosulfate	10040-45-6	-59.9053	-53.228	-6.6773	15.8736
T2712	Nifekalant hydrochloride	130656-51-8	-59.8935	-58.4255	-1.468	5.3895
T1067	Amphotericin B	1397-89-3	-59.8909	-53.022	-6.8689	40.7459
T0244	Tilorone dihydrochloride	27591-69-1	-59.775	-56.7544	-3.0206	7.2956
T1633	Beta-Carotene	7235-40-7	-59.7683	-59.4673	-0.3011	83.0627
T3630	Relugolix	737789-87-6	-59.762	-58.7494	-1.0126	19.63
T3606	Gamma-Oryzanol	11042-64-1	-59.7023	-59.5455	-0.1569	16.1654
T2239L	Raltegravir	518048-05-0	-59.6982	-52.1258	-7.5724	17.3788
T0706	Lisinopril dihydrate	83915-83-7	-59.6288	-48.6529	-10.976	17.8059
T2901	Daidzin	552-66-9	-59.5821	-54.5258	-5.0563	5.2064
T1773	Afatinib Dimaleate	850140-73-7	-59.5818	-56.7454	-2.8364	9.3038
T6542	Imidapril HCl	89396-94-1	-59.5741	-54.3622	-5.212	33.2078
T8144	Raffinose	512-69-6	-59.5636	-55.6694	-3.8942	32.5041
T7603	Pinaverium bromide	53251-94-8	-59.5429	-58.2522	-1.2907	17.1856
T6293	Mycophenolate Mofetil	128794-94-5	-59.5338	-56.1044	-3.4294	15.3276
T2775	Baicalin	21967-41-9	-59.4961	-55.1202	-4.3759	9.5405
T8128	Mosapride	112885-41-3	-59.304	-56.7751	-2.5289	6.033
T6986	Sivelestat	127373-66-4	-59.3038	-59.0455	-0.2583	17.2899
T2364	Irbinitinib	937263-43-9	-59.2885	-59.8987	0.6103	8.7789
T2368L	Edoxaban	912273-65-5	-59.2736	-55.7309	-3.5427	21.01
T1070	Cefmetazole sodium	56796-39-5	-59.2695	-52.7449	-6.5246	9.6912
T0104	Arbidol hydrochloride	131707-23-8	-59.2427	-57.0811	-2.1617	14.7141

T0486	Irinotecan hydrochloride trihydrate	136572-09-3	-59.1706	-56.9076	-2.263	13.4712
T6978	Almitrine dimesylate	29608-49-9	-59.1653	-58.7632	-0.4021	8.0718
T0053	Hydroxyzine Pamoate	10246-75-0	-59.1234	-51.2691	-7.8543	15.9981
T1510	Rosuvastatin calcium	147098-20-2	-59.1216	-52.806	-6.3157	11.9657
T6588	Mitoxantrone	65271-80-9	-59.0794	-48.9975	-10.0819	18.6501
T8690	Brexpiprazole HCl	913612-38-1	-59.0301	-57.0265	-2.0036	6.6284
T4992	Cefamandole nafate	42540-40-9	-59.0233	-54.7163	-4.307	17.3553
T5189	Omadacycline	389139-89-3	-59.0165	-44.3602	-14.6563	21.6149
T1259	Pyrantel pamoate	22204-24-6	-58.9678	-51.6546	-7.3132	16.9471
T1496	Amiodarone hydrochloride	19774-82-4	-58.952	-56.5621	-2.3899	14.1751
T2765	Rosmarinic acid	20283-92-5	-58.9282	-50.6115	-8.3167	3.4867
T6918	Olmutinib	1353550-13-6	-58.9125	-59.5609	0.6483	14.9271
T1010	Verapamil hydrochloride	152-11-4	-58.8821	-57.1013	-1.7808	36.7361
T1418	Bucladesine sodium salt	16980-89-5	-58.8667	-56.0399	-2.8269	8.5374
T0929	Docusate sodium	577-11-7	-58.7735	-59.8608	1.0873	15.6043
T1678	Nystatin	1400-61-9	-58.7387	-43.3242	-15.4145	39.8185
T0791	Reserpine	50-55-5	-58.6756	-57.09	-1.5856	12.0614
T7106	Menaquinone-4	863-61-6	-58.6471	-59.5538	0.9067	18.4414
T1676	Rosuvastatin	287714-41-4	-58.5789	-53.4696	-5.1094	18.4108
T2844	Hyperoside	482-36-0	-58.5741	-55.8311	-2.743	13.8169
T6841	Fumagillin	23110-15-8	-58.5365	-50.8612	-7.6753	20.4506
T0207	Ranolazine dihydrochloride	95635-56-6	-58.5361	-36.8702	-21.666	6.9759
T21312	Afatinib free base	850140-72-6	-58.5231	-54.2858	-4.2373	12.1655
T0097L	Pazopanib	444731-52-6	-58.5125	-57.5739	-0.9387	10.7154
T6199	Ribociclib	1211441-98-3	-58.5022	-52.0732	-6.429	16.7299
T3275	Nefazodone hydrochloride	82752-99-6	-58.4881	-57.2513	-1.2368	10.0658
T3139	Sultamicillin Tosylate	83105-70-8	-58.4869	-56.2093	-2.2776	19.6159
T1181	Gefitinib	184475-35-2	-58.4779	-57.8313	-0.6466	7.8941
T1192	Protoporphyrin IX	553-12-8	-58.4691	-57.7689	-0.7002	13.0943
T6670	Silymarin	65666-07-1	-58.4267	-53.7593	-4.6674	15.3544
T0777	Tilmicosin	108050-54-0	-58.4254	-54.3414	-4.084	36.9517
T1743	Aprepitant	170729-80-3	-58.4137	-39.4842	-18.9295	17.82
T3085	Piperacetazine	3819-00-9	-58.4098	-58.1186	-0.2912	15.3758
T2076	Pimavanserin	706779-91-1	-58.3913	-55.5586	-2.8327	17.1048
T5836	Eprinomectin	123997-26-2	-58.3896	-59.0731	0.6835	30.5777
T1457	Deferasirox	201530-41-8	-58.3849	-52.8455	-5.5394	5.7588
T2318	LCZ696	936623-90-4	-58.3577	-50.1252	-8.2325	28.5651
T6450	Clinofibrate	30299-08-2	-58.3147	-50.8595	-7.4552	34.2079
T6035	Odanacatib	603139-19-1	-58.3136	-48.1177	-10.196	16.8841
T2346	Enasidenib	1446502-11-9	-58.2626	-54.4953	-3.7674	5.8437
T2219	Puromycin dihydrochloride	58-58-2	-58.2475	-52.9358	-5.3117	10.5814
T2263	VX661	1152311-62-0	-58.2342	-56.7916	-1.4426	15.5383
T6280	Enzastaurin	170364-57-5	-58.2283	-58.5796	0.3513	13.1331
T19862	Voxilaprevir	1535212-07-7	-58.2279	-55.7837	-2.4442	26.0006
T6198	Dolutegravir	1051375-16-6	-58.1898	-51.1819	-7.0079	4.817
T2795	Amygdalin	29883-15-6	-58.1468	-52.6439	-5.5029	9.9906
T1903	Dabrafenib	1195765-45-7	-58.127	-57.3875	-0.7395	10.4458
T6486	Enalapril Maleate	76095-16-4	-58.1266	-52.5848	-5.5417	13.4797

T0918	Trimebutine	39133-31-8	-58.1263	-54.4417	-3.6846	19.4034
T1037	Doripenem Hydrate	364622-82-2	-58.1262	-47.1199	-11.0062	5.0945
T1022	Hygromycin B	31282-04-9	-58.1202	-37.6681	-20.4522	28.901
T7733	Cefotaxime	63527-52-6	-58.0464	-51.8882	-6.1582	14.4102
T1081	Nafronyl oxalate	3200-06-4	-58.0257	-55.685	-2.3407	14.5038
T2777	Ginsenoside Rg1	22427-39-0	-58.004	-56.386	-1.618	42.1794
T6232	Vilazodone HCl	163521-08-2	-57.9894	-54.8274	-3.1619	6.9381
T1279	Vilazodone	163521-12-8	-57.9787	-54.6241	-3.3546	6.0521
T5394	Prulifloxacin	123447-62-1	-57.9729	-50.3465	-7.6264	7.9959
T6946	Pimavanserin tartrate	706782-28-7	-57.9627	-56.266	-1.6967	17.3133
T5330	Fluralaner	864731-61-3	-57.9579	-57.4033	-0.5546	12.4866
T1626	Alprostadil	745-65-3	-57.8923	-51.6134	-6.2789	9.7663
T1487	Fluvastatin sodium salt	93957-55-2	-57.8519	-47.6245	-10.2273	18.7239
T6301	Tosedostat	238750-77-1	-57.8278	-51.4585	-6.3693	15.3517
T1660	Silibinin	22888-70-6	-57.7996	-51.5205	-6.2791	12.0275
T5396	Letermovir	917389-32-3	-57.7905	-57.5243	-0.2662	18.5823
T2483	Dacomitinib	1110813-31-4	-57.7811	-60.0998	2.3188	5.7398
T1213	Piperacillin sodium salt	59703-84-3	-57.7584	-56.5774	-1.181	19.5455
T0907	Sulfasalazine	599-79-1	-57.7033	-49.9423	-7.7609	4.1791
T2546	Pimozide	2062-78-4	-57.6923	-56.3389	-1.3535	17.3257
T0295	Digitoxin	71-63-6	-57.6913	-57.1242	-0.567	30.6589
T2402	Tianeptine sodium	30123-17-2	-57.691	-53.4171	-4.2739	12.4375
T1266	Terfenadine	50679-08-8	-57.5802	-57.4675	-0.1127	21.2351
T2656	Fruquintinib	1194506-26-7	-57.56	-57.837	0.2771	12.3667
T1016	Vitamin E Acetate	58-95-7	-57.5503	-57.7561	0.2057	18.5697
T3716	Rolapitant	552292-08-7	-57.5328	-56.2688	-1.264	19.1829
T13072	Taltirelin acetate	1549593-23-8	-57.4855	-57.584	0.0985	12.2593
T6999	Teneligliptin hydrobromide	906093-29-6	-57.4805	-39.8198	-17.6607	10.9577
T0979	Dequalinium chloride	522-51-0	-57.4777	-57.6106	0.1329	19.2623
T3213	Hematoporphyrin dihydrochloride	17696-69-4	-57.4691	-46.2869	-11.1822	12.026
T2768	Saikosaponin A	20736-09-8	-57.4623	-55.6926	-1.7697	20.4041
T1615	Irbesartan	138402-11-6	-57.4473	-52.0532	-5.3941	8.4278
T1533	Valganciclovir hydrochloride	175865-59-5	-57.4137	-54.3427	-3.071	12.5648
T1534	Darifenacin HBr	133099-07-7	-57.4056	-55.4034	-2.0023	24.4863
T4250	Bilastine	202189-78-4	-57.3598	-55.2768	-2.083	12.8337
T6698	Temocapril HCl	110221-44-8	-57.3289	-38.9511	-18.3778	10.5208
T1912	Dinaciclib	779353-01-4	-57.3227	-54.239	-3.0837	8.3669
T5S0754	Isoquercitrin	482-35-9	-57.3181	-54.188	-3.1301	12.2374
T0180	Benazepril hydrochloride	86541-74-4	-57.3082	-54.3902	-2.9181	13.7335
T6228	Irinotecan	97682-44-5	-57.2961	-55.3725	-1.9236	17.8169
T3625	ETC1002	738606-46-7	-57.2859	-47.0188	-10.2672	20.8462
T2595	Lumacaftor	936727-05-8	-57.2717	-58.0993	0.8276	12.7167
T3281	Delapril?Hydrochloride	83435-67-0	-57.2669	-52.3927	-4.8742	23.1676
T6S2023	Harpagoside	19210-12-9	-57.2517	-53.9655	-3.2861	13.0192
T3S0309	Raffinose	17629-30-0	-57.2188	-53.5419	-3.6768	15.1668
T4405	Ceftibuten dihydrate	118081-34-8	-57.21	-49.2627	-7.9472	7.6432
T0447	Carvedilol	72956-09-3	-57.1933	-52.5	-4.6933	8.7378
T2512	Mocetinostat	726169-73-9	-57.1837	-56.1836	-1.0001	6.7837

T3724	Rolapitant hydrochloride	914462-92-3	-57.1513	-53.2283	-3.923	21.0261
T3368	Alpha-Hederin	27013-91-8	-57.1214	-55.0934	-2.028	36.7621
T2319	Acotiamide hydrochloride	773092-05-0	-57.1208	-55.1818	-1.939	17.7373
TQ0084	Lumateperone Tosylate	1187020-80-9	-57.1194	-50.9318	-6.1876	5.2072
T1618	Dypyridamole	58-32-2	-57.1187	-55.6536	-1.4652	11.1861
T8222	Selpercatinib	2152628-33-4	-57.0458	-56.0991	-0.9467	12.0233
T0942	Quinacrine dihydrochloride	69-05-6	-57.0263	-55.6487	-1.3776	17.1468
T6825	Eltrombopag Olamine	496775-62-3	-56.9796	-52.2124	-4.7672	8.8673
T1554	Iohexol	66108-95-0	-56.97	-57.2806	0.3107	24.6757
T7217	EGT1442	1118567-05-7	-56.9568	-54.8715	-2.0853	11.856
T6452	Closantel Sodium	61438-64-0	-56.9431	-52.6205	-4.3226	10.9332
T3617	Ivosidenib	1448347-49-6	-56.8774	-56.5277	-0.3497	23.7654
T1890	Pracinostat	929016-96-6	-56.8679	-50.9971	-5.8708	14.6043
T8134	Cefathiamidine	33075-00-2	-56.8031	-54.6315	-2.1716	15.523
T7212	Delavirdine (mesylate)	147221-93-0	-56.7941	-56.0333	-0.7608	13.7827
T7225	AnaMorelin hydrochloride	861998-00-7	-56.7938	-55.5932	-1.2007	36.8733
T3489	Dasabuvir	1132935-63-7	-56.7602	-55.1254	-1.6348	28.0037
T1197	Trimebutine maleate	34140-59-5	-56.7567	-55.2315	-1.5252	22.498
T5030	Zofenopril (calcium)	81938-43-4	-56.7248	-57.3243	0.5996	10.4731
T7584	zanubrutinib	1691249-45-2	-56.704	-56.98	0.276	14.7002
T6018	Zosuquidar 3HCl	167465-36-3	-56.6605	-51.9	-4.7605	6.0445
T7975	Rhodamine?B	81-88-9	-56.6591	-52.2811	-4.3781	12.6641
T4546	Dinoprost Tromethamine	38562-01-5	-56.6566	-49.836	-6.8206	10.2236
T2544	Bazedoxifene acetate	198481-33-3	-56.6356	-56.9038	0.2682	13.8257
T3663	Eliglustat	491833-29-5	-56.6149	-54.8967	-1.7182	7.2311
T6453	Conivaptan hydrochloride	168626-94-6	-56.6025	-56.6761	0.0736	16.3186
T4610	BMS927711	1289023-67-1	-56.598	-52.1505	-4.4475	25.585
T0210	Tamsulosin hydrochloride	106463-17-6	-56.5766	-53.3026	-3.2739	11.5211
T0956	Abamectin	71751-41-2	-56.5392	-57.0556	0.5164	26.0015
T6715	Valnemulin HCl	133868-46-9	-56.4391	-53.2995	-3.1396	18.6524
T6716	Valsartan	137862-53-4	-56.4115	-47.4673	-8.9442	25.9479
T0108	Bumetanide	28395-03-1	-56.4045	-49.695	-6.7095	12.9812
T1278	Astemizole	68844-77-9	-56.3881	-55.8829	-0.5052	10.5064
T6334	Ridaforolimus	572924-54-0	-56.3767	-56.8373	0.4606	32.5247
T8651	Idelalisib	1146702-54-6	-56.3735	-54.655	-1.7185	9.908
T0133	Cefdinir	91832-40-5	-56.3497	-52.7319	-3.6178	6.4227
T1894	Idelalisib	870281-82-6	-56.3414	-54.598	-1.7434	11.5616
T2239	Raltegravir potassium	871038-72-1	-56.341	-54.9267	-1.4143	16.0533
T3004	Batyl alcohol	544-62-7	-56.3372	-54.2952	-2.042	9.4197
T3034	Eprazinone dihydrochloride	10402-53-6	-56.332	-54.7119	-1.6201	5.8835
T0401	Benzethonium chloride	121-54-0	-56.316	-53.7773	-2.5388	21.3044
T0216	Eletriptan HBr	177834-92-3	-56.2744	-54.0256	-2.2489	7.1272
T4551	Acrivastine	87848-99-5	-56.266	-53.1965	-3.0696	5.8361
T6930	Pazopanib HCl	635702-64-6	-56.2413	-55.5991	-0.6422	11.2216
T1557	Acetylspiramycin	24916-51-6	-56.2371	-57.2449	1.0078	41.4877
T5038	Cephapirin Sodium	24356-60-3	-56.2045	-51.2559	-4.9486	14.2246
T2583	Cilastatin	82009-34-5	-56.1811	-51.3284	-4.8528	7.0976
T0334	Rosiglitazone	122320-73-4	-56.1422	-51.7622	-4.38	4.9123

T8654	Plx-4032	1029872-54-5	-56.1414	-56.402	0.2606	8.0756
T2136	Geniposidic Acid	27741-01-1	-56.1276	-46.5835	-9.5441	8.0452
T6271	Tipifarnib	192185-72-1	-56.1169	-53.8403	-2.2766	23.0248
T1434	Clarithromycin	81103-11-9	-56.0928	-53.4022	-2.6906	57.5395
T7714	Temocapil	111902-57-9	-56.0684	-48.7453	-7.3232	11.6177
T7000	Tiagabine hydrochloride	145821-59-6	-56.0615	-52.1728	-3.8887	5.8796
T2742	Aloin	1415-73-2	-56.0254	-51.8497	-4.1757	27.1804
T2336	Menatetrenone	11032-49-8	-56.0067	-56.8132	0.8065	15.475
T2499	Torcetrapib	262352-17-0	-56.0043	-55.1747	-0.8296	35.0143
T2382	Vemurafenib	918504-65-1	-55.9841	-56.4165	0.4324	12.937
T0091	Alfuzosin hydrochloride	81403-68-1	-55.9791	-56.3485	0.3694	9.7148
T0672	Pravastatin sodium	81131-70-6	-55.9504	-44.8487	-11.1016	13.2385
T0992	Carbenoxolone disodium	7421-40-1	-55.9384	-35.8822	-20.0562	11.4207
T2383	Panobinostat	404950-80-7	-55.8991	-50.875	-5.0242	4.4937
T1452	Axitinib	319460-85-0	-55.8852	-56.1607	0.2754	4.6534
T4576	Perospirone	150915-41-6	-55.8236	-49.1901	-6.6334	8.0772
T2391	Camostat mesilate	59721-29-8	-55.8125	-52.0483	-3.7642	11.8891
T0346	Camylofin	54-30-8	-55.76	-47.981	-7.779	11.3049
T0819	Spiramycin	8025-81-8	-55.7463	-54.6347	-1.1116	30.7829
T0681	Rifampicin	13292-46-1	-55.7283	-53.0675	-2.6608	58.8713
T0044	Oxethazaine	126-27-2	-55.7243	-52.2451	-3.4792	45.1758
T1766	Empagliflozin	864070-44-0	-55.6758	-52.9297	-2.7461	7.084
T6231	Tebipenem Pivoxil	161715-24-8	-55.673	-54.1879	-1.4851	13.1225
T2899	Liquiritin	551-15-5	-55.6461	-51.3056	-4.3406	10.7284
T1292	Tiapride hydrochloride	51012-33-0	-55.5982	-53.5421	-2.056	9.7908
T0971	Dirithromycin	62013-04-1	-55.5579	-44.4936	-11.0643	82.2225
T6492	Davercin	55224-05-0	-55.5569	-52.7923	-2.7645	40.5097
T1142	Clindamycin phosphate	24729-96-2	-55.4788	-39.024	-16.4548	20.1721
T5014	Prostaglandin E2 (PGE2)	363-24-6	-55.466	-49.7753	-5.6907	9.3175
T1539	Iloperidone	133454-47-4	-55.4395	-55.1574	-0.2821	13.77
T1929	Filgotinib	1206161-97-8	-55.4256	-54.6366	-0.7889	10.0307
T3211	Midostaurin	120685-11-2	-55.4254	-55.6895	0.2641	37.8225
T0864	L-Thyroxine	51-48-9	-55.4206	-44.7495	-10.6711	10.4558
T8063	Cephapirin Benzathine	97468-37-6	-55.4116	-48.5881	-6.8235	6.7099
T2S1464	Thiocolchicoside	602-41-5	-55.4051	-55.1615	-0.2436	14.1679
T2542	Prucalopride	179474-81-8	-55.3532	-53.2215	-2.1317	4.3023
T6958	Prucalopride Succinate	179474-85-2	-55.314	-52.4669	-2.8471	5.9484
T1576	Iopamidol	60166-93-0	-55.2693	-53.9814	-1.2879	14.1468
T0519	Euquinine	83-75-0	-55.2644	-53.5086	-1.7558	11.4785
T0462	Cilostazol	73963-72-1	-55.236	-54.2016	-1.0344	7.6045
T0231	Amlodipine Besylate	111470-99-6	-55.2238	-52.373	-2.8508	14.3754
T8404	Kasugamycin hydrochloride hydrate	200132-83-8	-55.2226	-28.6073	-26.6153	6.5818
T5109	Avapritinib	1703793-34-3	-55.2189	-39.2733	-15.9456	9.2333
T7066	Fosphenytoin sodium	92134-98-0	-55.2085	-45.9098	-9.2987	12.0431
T2396	Ramatroban	116649-85-5	-55.1946	-49.4413	-5.7533	8.3354
T1648	Vitamin E	59-02-9	-55.1725	-54.4601	-0.7123	19.711
T6560	Lappaconite HBr	97792-45-5	-55.1373	-40.5106	-14.6267	27.0905
T1211	Levosulpiride	23672-07-3	-55.1344	-53.6926	-1.4417	14.379

T2500	Cediranib	288383-20-0	-55.127	-51.9945	-3.1325	10.9093
T2332	Elvitegravir	697761-98-1	-55.1249	-51.1332	-3.9917	13.4748
T2562	Eltrombopag	496775-61-2	-55.1211	-45.3906	-9.7305	8.5255
T6302	Lonafarnib	193275-84-2	-55.1169	-54.1358	-0.9811	23.6053
T7133	Anagliptin	739366-20-2	-55.0991	-54.4471	-0.652	5.9131
T6541	Ibutilide Fumarate	122647-32-9	-55.087	-52.7304	-2.3566	11.6223
T2973	Astragaloside IV	84687-43-4	-55.0609	-53.2799	-1.781	32.4081
T8406	Gadobutrol	770691-21-9	-55.0508	-40.8375	-14.2132	16.4812
T3266	Lumefantrine	82186-77-4	-54.9991	-54.5263	-0.4728	15.1108
T0381	Amodiaquine hydrochloride	6398-98-7	-54.9628	-43.1522	-11.8106	5.6229
T0884	n-Docosanol	661-19-8	-54.9456	-55.005	0.0594	13.28
T0049	Azlocillin sodium	37091-65-9	-54.9291	-46.3012	-8.6279	12.8904
T1090	Perphenazine	58-39-9	-54.9074	-55.9595	1.0521	9.1369
T2329	Dolutegravir sodium	1051375-19-9	-54.8755	-48.7514	-6.1241	2.6687
T2306	Brexpiprazole	913611-97-9	-54.8642	-53.5709	-1.2933	5.434
T1988	Duvelisib	1201438-56-3	-54.8594	-54.2403	-0.6191	9.7637
T6646	Rosiglitazone HCl	302543-62-0	-54.8533	-49.9648	-4.8886	6.3994
T0082	Domperidone	57808-66-9	-54.8439	-55.6567	0.8128	19.2991
T4574	AHU377	149709-62-6	-54.8262	-50.753	-4.0732	15.5747
T0348	Labetalol hydrochloride	32780-64-6	-54.7779	-43.9218	-10.8561	4.3067
T8064	Lactobionic acid	96-82-2	-54.7732	-47.0185	-7.7547	8.2646
T2360	Baricitinib?Phosphate	1187595-84-1	-54.7665	-54.7624	-0.0041	5.6336
T6240	Palbociclib Isethionate	827022-33-3	-54.7282	-48.5258	-6.2024	16.0287
T6016	Maraviroc	376348-65-1	-54.7275	-53.6372	-1.0903	23.1992
T0868	Cefepime Dihydrochloride Monohydrate	123171-59-5	-54.7186	-44.0066	-10.7121	8.0365
T0811	Amisulpride	71675-85-9	-54.7058	-51.4007	-3.3051	9.0697
T1597	Proadifen hydrochloride	62-68-0	-54.6727	-52.9992	-1.6735	22.6859
T1138	Taurocholic acid sodium salt hydrate	345909-26-4	-54.6619	-51.6395	-3.0224	19.5235
T0512	Phthalylsulfathiazole	85-73-4	-54.6272	-50.1518	-4.4754	8.973
TMA2394	DL-??-Tocopherol	10191-41-0	-54.5746	-53.8698	-0.7048	16.31
T1031	Cloxacillin sodium monohydrate	7081-44-9	-54.5155	-53.0396	-1.4759	9.8968
T1242	Alvimopan	156053-89-3	-54.5118	-49.1329	-5.3789	13.3734
T1593	Ezetimibe	163222-33-1	-54.5109	-53.6468	-0.8641	12.0654
T2805	Chlorogenic Acid	327-97-9	-54.4839	-48.9279	-5.556	19.2367
T1033	Monensin sodium salt	22373-78-0	-54.4643	-47.907	-6.5573	35.217
T0237	Closantel	57808-65-8	-54.4599	-53.4707	-0.9892	11.3799
T2532	Tauroursodeoxycholic Acid	14605-22-2	-54.4326	-51.3227	-3.1099	9.5154
T0999	Clevidipine butyrate	167221-71-8	-54.4171	-55.2951	0.878	19.5
T1516	Curcumin	458-37-7	-54.3873	-51.6106	-2.7768	7.634
T4980	Betrixaban maleate	936539-80-9	-54.3864	-52.6287	-1.7577	10.0952
T0209	Loperamide hydrochloride	34552-83-5	-54.3361	-55.6425	1.3064	34.6201
T0194	Chloroquine diphosphate	50-63-5	-54.3245	-30.0565	-24.2681	7.061
T1963	Capmatinib	1029712-80-8	-54.3229	-54.0193	-0.3035	4.6609
T2534	Pitavastatin calcium	147526-32-7	-54.2778	-50.3341	-3.9437	17.0816
T3402	Ginsenoside Rg3	14197-60-5	-54.2534	-53.413	-0.8404	32.0632
T1522	Iopromide	73334-07-3	-54.2347	-52.1225	-2.1122	16.469
T1105	Penfluridol	26864-56-2	-54.2252	-53.5554	-0.6698	14.738
T4284	(S)-Amlodipine Besylate (103129-82-4(free base))	T4284	-54.1651	-48.9968	-5.1683	12.928

T3265	Azathramycin	76801-85-9	-54.1603	-52.739	-1.4213	58.0043
T2677	Crenolanib	670220-88-9	-54.1567	-46.5277	-7.629	16.5032
T1835	Ibrutinib	936563-96-1	-54.1513	-54.5835	0.4322	14.4588
T2980	Chondroitin sulfate	9007-28-7	-54.1472	-51.1736	-2.9737	8.0505
T7551	Belotecan hydrochloride	213819-48-8	-54.1313	-53.6407	-0.4907	10.4751
T6501	Fludarabine Phosphate	75607-67-9	-54.1238	-40.6718	-13.452	5.8873
T1566	Aripiprazole	129722-12-9	-54.1141	-50.7446	-3.3695	4.217
T6788	Bitopertin	845614-11-1	-54.1035	-54.0511	-0.0524	14.107
T0905	Pyrithioxin	1098-97-1	-54.1017	-50.2831	-3.8186	8.9735
T6405	Bepotastine Besilate	190786-44-8	-54.0928	-51.2629	-2.83	19.1424
T1001	Dicloxacillin sodium monohydrate	13412-64-1	-54.0313	-51.8839	-2.1474	11.0349
T2368	Edoxaban Tosylate Monohydrate	1229194-11-9	-54.0277	-56.186	2.1583	13.2373
T1087	Valacyclovir hydrochloride	124832-27-5	-54.009	-51.2045	-2.8045	7.1792
T0342	Carvedilol phosphate	610309-89-2	-54.0078	-48.5154	-5.4924	8.2073
T6665	Sertaconazole nitrate	99592-39-9	-53.9918	-53.2921	-0.6998	8.4527
T1201	Sulpiride	15676-16-1	-53.9815	-53.0412	-0.9404	4.5723
T1921	Alpelisib	1217486-61-7	-53.98	-53.5262	-0.4538	18.7616
T0841	Bezafibrate	41859-67-0	-53.9798	-47.3299	-6.6499	10.989
T6955	Propafenone HCl	34183-22-7	-53.9693	-54.8679	0.8986	8.3865
T4341	Betrixaban	330942-05-7	-53.9575	-52.1841	-1.7734	12.4903
T5037	Midecamycin	35457-80-8	-53.9529	-55.4785	1.5256	56.2332
T1176	Racecadotril	81110-73-8	-53.9526	-53.4655	-0.4871	11.1547
T4615	Balsalazide disodium salt dihydrate	150399-21-6	-53.9482	-44.6052	-9.343	3.7816
T1085	Glutathione	70-18-8	-53.935	-43.5438	-10.3911	5.7678
T1735	Lurasidone hydrochloride	367514-88-3	-53.932	-55.6173	1.6854	13.9805
T0068	Fluphenazine hydrochloride	146-56-5	-53.9243	-46.4318	-7.4925	7.8307
T6796	CB5083	1542705-92-9	-53.9143	-53.4821	-0.4321	9.2867
T1118	Cefprozil hydrate	121123-17-9	-53.895	-41.496	-12.399	8.3652
T0323	Oxantel Pamoate	68813-55-8	-53.8712	-48.1945	-5.6767	15.3755
T5006	Flucloxacillin sodium	1847-24-1	-53.8678	-52.6425	-1.2253	7.4399
T6499	Fenticonazole Nitrate	73151-29-8	-53.8581	-53.7747	-0.0834	15.1917
T0911	Cefotaxime sodium	64485-93-4	-53.8516	-47.4649	-6.3867	9.7567
T6915	Darolutamide	1297538-32-9	-53.8479	-53.0205	-0.8274	5.1458
T2580	Formoterol	73573-87-2	-53.8355	-50.7295	-3.106	6.9575
T6968	Riociguat	625115-55-1	-53.824	-53.587	-0.237	9.6903
T0215L	Losartan	114798-26-4	-53.8206	-50.1813	-3.6393	21.8224
T6998	Tedizolid Phosphate	856867-55-5	-53.8199	-38.6121	-15.2078	4.6338
T5023	Propiverine hydrochloride	54556-98-8	-53.8174	-50.8964	-2.921	26.4936
T1439	Lacidipine	103890-78-4	-53.8066	-54.3971	0.5905	33.8183
T3632	Doravirine	1338225-97-0	-53.7427	-49.8882	-3.8545	13.2864
T1239	Indacaterol maleate	753498-25-8	-53.7272	-54.9189	1.1918	17.259
T6239	Palbociclib hydrochloride	827022-32-2	-53.7153	-51.2287	-2.4866	13.8401
T0727	Piperonyl butoxide	51-03-6	-53.714	-54.1091	0.3951	9.2646
T0118	Mizolastine	108612-45-9	-53.6788	-54.0789	0.4001	14.2392
T22314	Doripenem	148016-81-3	-53.6739	-46.1039	-7.57	4.8807
T7581	voxelotor	1446321-46-5	-53.6496	-52.0639	-1.5857	8.2744
T1469	Levocetirizine	130018-77-8	-53.6398	-50.0215	-3.6183	6.4527
T0703	Flavoxate hydrochloride	3717-88-2	-53.6382	-54.1003	0.4621	9.9128

T8416	Capmatinib hydrochloride(free base)	1029714-89-3	-53.6258	-52.3486	-1.2771	9.3573
T1780	Parecoxib	198470-84-7	-53.6121	-48.5375	-5.0746	6.7066
T0343	Nimodipine	66085-59-4	-53.5909	-52.6358	-0.9551	16.3338
T0325	Diperodon hydrochloride	537-12-2	-53.5747	-51.1795	-2.3952	8.6843
T2871	Quercitrin	522-12-3	-53.5705	-46.4519	-7.1186	14.1712
T2574	Alizapride hydrochloride	59338-87-3	-53.5525	-50.8399	-2.7125	5.6812
T5009	Trimethobenzamide hydrochloride	554-92-7	-53.5421	-40.4585	-13.0836	7.9171
T2399	Bortezomib	179324-69-7	-53.5356	-53.5356	0	12.4975
T0520	Lenvatinib	417716-92-8	-53.4959	-52.8486	-0.6473	9.8692
T0368	Cinepazide maleate	26328-04-1	-53.4875	-50.9551	-2.5324	13.7779
T2456	Tivozanib	475108-18-0	-53.4845	-52.2595	-1.225	12.3141
T0373L	Erlotinib hydrochloride	183319-69-9	-53.4514	-52.982	-0.4694	4.6596
T1784	Everolimus	159351-69-6	-53.4268	-53.3606	-0.0662	27.4685
T0165	Urapidil	34661-75-1	-53.4101	-49.6392	-3.7709	6.711
T1792	Regorafenib	755037-03-7	-53.4095	-54.7107	1.3012	9.9606
T2522	Peramivir Trihydrate	1041434-82-5	-53.395	-50.9349	-2.4601	20.2247
T1206	Amsacrine	51264-14-3	-53.392	-50.5374	-2.8546	11.6927
T0709	Pimaricin	7681-93-8	-53.3724	-43.9528	-9.4196	19.6225
T3015	Olaparib	763113-22-0	-53.3584	-52.272	-1.0863	14.6458
T0402	Octocrilene	6197-30-4	-53.3471	-52.5846	-0.7625	11.5484
T0093L	Sorafenib	284461-73-0	-53.3097	-55.0685	1.7588	8.557
T0093	Sorafenib tosylate	475207-59-1	-53.2951	-54.8592	1.564	9.8214
T6505	Formoterol Hemifumarate	43229-80-7	-53.2928	-48.6647	-4.6281	6.3496
T1224	Cefuroxime sodium	56238-63-2	-53.283	-49.8126	-3.4704	7.7528
T4226	Amenamevir	841301-32-4	-53.2636	-52.3805	-0.8831	20.9594
T2438	HMN214	173529-46-9	-53.2628	-51.4205	-1.8423	23.2574
T4690	Betrixaban hydrochloride(330942-05-7(free base))	T4690	-53.2577	-51.4095	-1.8482	13.793
T1785	Palbociclib	571190-30-2	-53.244	-51.3407	-1.9033	12.7101
T0215	Losartan potassium	124750-99-8	-53.2439	-47.205	-6.039	12.3774
T1290	Oxiconazole nitrate	64211-46-7	-53.2374	-52.3359	-0.9015	9.5957
T4063	Delafloxacin	189279-58-1	-53.227	-51.7523	-1.4747	11.7778
T1257	Ubenimex	58970-76-6	-53.2211	-43.7608	-9.4603	7.608
T2554	Etofibrate	31637-97-5	-53.1631	-50.9636	-2.1995	9.1813
T6401	Azithromycin	83905-01-5	-53.1239	-52.2903	-0.8337	78.6831
T1032	Erythromycin	114-07-8	-53.1109	-48.8204	-4.2905	59.2206
T0089	Cetirizine dihydrochloride	83881-52-1	-53.0951	-49.4379	-3.6573	5.9589
T2582	Apatinib	811803-05-1	-53.0832	-52.9306	-0.1526	10.594
T8143	Bevantolol hydrochloride	42864-78-8	-53.0828	-37.9289	-15.1539	15.528
T0088	Urapidil hydrochloride	64887-14-5	-53.0697	-52.9562	-0.1136	8.9112
T2389	Dapagliflozin	461432-26-8	-53.0481	-48.7141	-4.334	12.2999
T2539	Fingolimod hydrochloride	162359-56-0	-53.0469	-35.6852	-17.3617	9.939
T4493	Bictegravir	1611493-60-7	-53.0404	-45.5298	-7.5106	11.9679
T0853L	Adenosine 5'-monophosphate monohydrate	18422-05-4	-53.0359	-38.0702	-14.9657	6.3138
T1312	nafcillin sodium	7177-50-6	-53.0193	-51.9157	-1.1037	10.3345
T5858	Sertindole	106516-24-9	-53.0114	-55.3518	2.3405	11.8747
T3529	Bestatin hydrochloride	65391-42-6	-53.001	-43.9408	-9.0602	10.0114
T6387	Ampiroxicam	99464-64-9	-52.9941	-51.883	-1.1111	12.1055
T2553	Etofenamate	30544-47-9	-52.9716	-51.9288	-1.0428	6.8563

T1492	Gemifloxacin?mesylate	210353-53-0	-52.9332	-48.3192	-4.6139	8.0465
T7744	Piperaquine tetraphosphate tetrahydrate	915967-82-7	-52.929	-47.3754	-5.5536	10.5942
T0366	Cefadroxil	50370-12-2	-52.8869	-40.5538	-12.3331	6.4257
T1622	Rosiglitazone maleate	155141-29-0	-52.8729	-48.0114	-4.8614	5.1324
T0896	Furaltadone hydrochloride	3759-92-0	-52.8602	-49.3004	-3.5598	4.7722
T1385	Amlodipine	88150-42-9	-52.8411	-51.2576	-1.5834	16.5215
T1005L	Amoxicillin trihydrate	61336-70-7	-52.7952	-39.7046	-13.0906	6.6871
T0806	Flunarizine dihydrochloride	30484-77-6	-52.7677	-49.2896	-3.478	6.9969
T2564	Flupenthixol dihydrochloride	51529-01-2	-52.7673	-48.8665	-3.9008	11.6034
T0865	Ranitidine Hydrochloride	66357-59-3	-52.7485	-49.2473	-3.5012	13.9959
T5857	Trametinib DMSO solvate	1187431-43-1	-52.7432	-51.9851	-0.7581	28.266
T5153	Apatinib mesylate	1218779-75-9	-52.7389	-52.4394	-0.2996	9.2676
T5293	Maltotriose	1109-28-0	-52.7281	-49.6202	-3.1079	10.2743
T7147	Valbenazine	1025504-45-3	-52.7176	-40.6521	-12.0655	13.8692
T2144	Tacrolimus	104987-11-3	-52.6827	-52.5566	-0.1261	28.5483
T0157	Roxatidine Acetate hydrochloride	93793-83-0	-52.6618	-50.8484	-1.8134	4.1038
T5135	Fupentixol Dihydrochloride	2413-38-9	-52.6553	-48.1505	-4.5048	11.237
T6472	Difluprednate	23674-86-4	-52.6514	-50.1337	-2.5176	18.6872
T2854	Forsythin	487-41-2	-52.6228	-50.2425	-2.3803	14.8626
T1005	Amoxicillin	26787-78-0	-52.6173	-39.3109	-13.3064	6.9168
T2320	Indacaterol	312753-06-3	-52.5786	-51.3268	-1.2517	18.1471
T5842	Bromocriptine mesylate	22260-51-1	-52.5584	-53.9376	1.3792	27.6797
T8046	Tosufloxacin p-Toluenesulfonate Hydrate	1400591-39-0	-52.5137	-45.1337	-7.38	5.6545
T1756	Ilaprazole sodium	172152-50-0	-52.4613	-45.2801	-7.1812	9.4453
T1467	Acemetacin	53164-05-9	-52.447	-45.4818	-6.9652	19.0348
T4503	Talniflumate	66898-62-2	-52.4468	-51.349	-1.0978	5.914
T5460	Mofezolac	78967-07-4	-52.4141	-54.3267	1.9126	9.275
T4552	Fursultiamine	804-30-8	-52.4071	-51.0794	-1.3277	8.6005
T1132	Nicergoline	27848-84-6	-52.4028	-51.0014	-1.4014	8.45
T8136	Perospirone hydrochloride	129273-38-7	-52.3858	-55.0436	2.6579	9.0954
T1926	NVP-LDE225	956697-53-3	-52.3751	-51.7573	-0.6178	8.2086
T6340	Enalaprilat Dihydrate	84680-54-6	-52.3734	-47.4184	-4.955	15.5045
T0749	Succinylsulfathiazole	116-43-8	-52.3711	-46.445	-5.9261	6.8137
T0919	Benfluorex hydrochloride	23642-66-2	-52.334	-50.0377	-2.2963	10.2703
T1049L	Oxybutynin	5633-20-5	-52.3217	-51.2013	-1.1204	17.861
T0985	Oxacillin sodium	1173-88-2	-52.3056	-46.1233	-6.1823	7.4363
T0782	Benzthiazide	91-33-8	-52.289	-51.4218	-0.8672	6.7193
T6571	Lomerizine hydrochloride	101477-54-7	-52.2458	-52.3034	0.0576	14.2865
T7093	Rafoxanide	22662-39-1	-52.2429	-51.6442	-0.5988	6.8866
T8689	CHLOROQUINE	54-05-7	-52.2039	-29.3442	-22.8597	8.1677
T1049	Oxybutynin hydrochloride	1508-65-2	-52.2002	-49.7818	-2.4184	16.8977
T1184	Rivaroxaban	366789-02-8	-52.199	-52.5829	0.3839	9.5686
T1505	Imipenem	74431-23-5	-52.196	-33.2454	-18.9507	5.8206
T1332	Permethrin	52645-53-1	-52.1809	-52.4464	0.2656	16.9058
T4501	Ceftizoxime	68401-81-0	-52.1634	-45.337	-6.8264	6.9664
T2A2481	Taurochenodeoxycholic Acid	516-35-8	-52.1623	-49.4142	-2.7481	16.1762
T0055	Glafenine	3820-67-5	-52.1609	-45.7948	-6.366	5.2892
T3457	Olodaterol	868049-49-4	-52.1552	-48.4979	-3.6573	11.496

T8066	Sulbenicillin Sodium	28002-18-8	-52.1302	-50.3659	-1.7644	14.0437
T8402	Regorafenib Hydrochloride	835621-07-3	-52.1286	-52.4659	0.3373	9.7223
T5159	BI 1744 hydrochloride	869477-96-3	-52.109	-51.795	-0.314	9.2023
T0072	Adiphenine hydrochloride	50-42-0	-52.1076	-49.4393	-2.6683	10.6466
T7405	Piperidolate	82-98-4	-52.0985	-49.4459	-2.6526	6.5596
T0214	Pioglitazone	111025-46-8	-52.0291	-50.5892	-1.4399	6.9587
T1575	Nizatidine	76963-41-2	-52.0167	-49.7624	-2.2543	10.8344
T3289	Amifostine	20537-88-6	-52.0088	-19.0208	-32.9881	4.553
T5391	Bepridil hydrochloride	68099-86-5	-52.0053	-51.6704	-0.3348	12.5927
T0412	Idebenone	58186-27-9	-51.9877	-52.6947	0.707	11.2378
T0214L	Pioglitazone hydrochloride	112529-15-4	-51.9872	-50.6816	-1.3056	8.0797
T6106	Selinexor	1393477-72-9	-51.9864	-51.9692	-0.0173	7.7056
T2447	AZD7545	252017-04-2	-51.9578	-50.62	-1.3379	11.3242
T1063	Ticarcillin disodium	4697-14-7	-51.9524	-47.2097	-4.7427	15.3555
T6218	Selumetinib	606143-52-6	-51.9088	-51.0872	-0.8216	5.8034
T2831	Glycocholic acid	475-31-0	-51.9051	-50.3909	-1.5142	25.6616
T6436	Cefaclor	53994-73-3	-51.8934	-45.6843	-6.2091	7.4086
T0344L	Miconazole nitrate	22832-87-7	-51.881	-51.0906	-0.7905	13.0049
T0385	Prochlorperazine Maleate	84-02-6	-51.8723	-51.9283	0.056	10.5804
T1107	Sarafloxacin hydrochloride	91296-87-6	-51.8491	-43.7795	-8.0695	9.2648
T0374L	Sunitinib	557795-19-4	-51.8335	-49.325	-2.5085	11.6053
T2485	Baricitinib	1187594-09-7	-51.8127	-52.5624	0.7496	4.9879
T0224	Meropenem	96036-03-2	-51.7612	-32.1639	-19.5973	14.827
T4997	Umeclidinium bromide	869113-09-7	-51.7554	-49.6789	-2.0765	22.5984
T4460	Dapagliflozin ((2S)-1,2-propanediol, hydrate)	960404-48-2	-51.7377	-48.061	-3.6767	8.7825
T1782	Canagliflozin	842133-18-0	-51.7316	-49.5754	-2.1562	9.2726
T7394	Toceranib Phosphate	874819-74-6	-51.7134	-50.5258	-1.1876	12.243
T1669	Liothyronine sodium	55-06-1	-51.7081	-49.9165	-1.7916	12.9364
T1335	Mycophenolic acid	24280-93-1	-51.7055	-45.3685	-6.3371	13.2713
T1006	Propantheline bromide	50-34-0	-51.6942	-52.6319	0.9377	21.801
T2515	Roxadustat	808118-40-3	-51.6866	-45.8019	-5.8848	6.7312
T6S0722	Isoorientin	4261-42-1	-51.684	-47.2206	-4.4634	13.4092
T0103	Dipivefrin hydrochloride	64019-93-8	-51.6781	-48.4607	-3.2175	11.9698
T1736	Apixaban	503612-47-3	-51.6733	-51.2397	-0.4336	6.5084
T7964	Palatinitol	64519-82-0	-51.6664	-47.9839	-3.6825	6.1789
T0280	Spiperone	749-02-0	-51.6662	-52.2426	0.5764	12.5053
T1062	Capsaicin	404-86-4	-51.658	-47.456	-4.202	4.9204
T1645	Ramipril	87333-19-5	-51.6521	-50.4657	-1.1864	11.7734
T2762	Kasugamycin hydrochloride	19408-46-9	-51.6283	-31.2129	-20.4154	5.7604
T5820	Amsacrine hydrochloride	54301-15-4	-51.6087	-48.5226	-3.0861	10.3127
T0055L	Glafenine Hydrochloride	65513-72-6	-51.6056	-46.2284	-5.3772	6.6836
T1008	Cephalexin	15686-71-2	-51.5884	-44.3876	-7.2009	8.5492
T5328	Fosfluconazole	194798-83-9	-51.5596	-43.3862	-8.1734	31.5889
T0161	Pantoprazole Sodium Hydrate	164579-32-2	-51.5592	-45.1571	-6.4021	11.0237
T0150	Hydroxyhexamide	3168-01-2	-51.5564	-43.4326	-8.1238	5.3226
T1562	Rebamipide	90098-04-7	-51.5468	-50.6501	-0.8968	7.9213
T5008	Teprenone	6809-52-5	-51.5452	-51.2781	-0.2671	12.3436
T6943	Picropodophyllin	477-47-4	-51.53	-52.8984	1.3685	12.5535

T2125	Trametinib	871700-17-3	-51.5294	-52.5043	0.9749	25.396
T0409	Phthalylsulfacetamide	131-69-1	-51.5274	-42.6665	-8.8609	8.5832
T4054	Dantrolene sodium hemiheptahydrate	24868-20-0	-51.5227	-44.6351	-6.8876	4.9184
T2914	Melibiose	585-99-9	-51.4885	-48.2603	-3.2282	5.0827
T1629	Rifapentine	61379-65-5	-51.4849	-52.186	0.7011	54.2915
T2163	Dihydrocapsaicin	19408-84-5	-51.4833	-46.9779	-4.5054	11.4884
T6195	Baloxavir marboxil	1985606-14-1	-51.4795	-52.109	0.6295	23.8722
T0212	Seratrodast	112665-43-7	-51.4761	-46.5606	-4.9155	17.7387
T17035	Temoporfin	122341-38-2	-51.4575	-51.0203	-0.4372	15.159
T0866	Propafenone	54063-53-5	-51.4217	-48.1134	-3.3084	6.1671
T1122	Cephalothin sodium	58-71-9	-51.4134	-45.8692	-5.5442	6.9849
T6S1373	Vitexicarpin	479-91-4	-51.3835	-44.6863	-6.6973	18.801
T2571	Benactyzine hydrochloride	57-37-4	-51.3293	-48.8032	-2.526	15.5908
T1023	Cefoxitin sodium	33564-30-6	-51.3196	-48.4624	-2.8572	14.0903
T0344	Miconazole	22916-47-8	-51.298	-50.6168	-0.6812	9.4782
T1109	Zucapsaicin	25775-90-0	-51.2901	-47.1901	-4.1	6.1842
T7369	Gemigliptin	911637-19-9	-51.2696	-50.2605	-1.0091	14.1199
T3607	Kitasamycin	1392-21-8	-51.264	-51.7038	0.4398	44.0252
T8133	SulfadiMethoxine sodium	1037-50-9	-51.2579	-41.2541	-10.0039	4.0305
T3547	Sotagliflozin	1018899-04-1	-51.2415	-49.2212	-2.0203	11.3499
T1641	Strontium ranelate	135459-87-9	-51.2301	-45.3874	-5.8428	8.3245
T2585	Bedaquiline	843663-66-1	-51.2256	-48.6079	-2.6177	24.8052
T2270	Trans-Tranilast	70806-55-2	-51.2172	-45.1015	-6.1157	10.3624
T2200	Hydroxyzine dihydrochloride	2192-20-3	-51.2127	-30.7569	-20.4558	6.6895
T3524	Halofuginone hydrobromide	64924-67-0	-51.2069	-34.725	-16.4819	5.9638
T1093	Adapalene	106685-40-9	-51.1972	-49.3593	-1.8379	16.5023
T2115	Pexidartinib	1029044-16-3	-51.1895	-50.3855	-0.804	3.8953
T8389	Trifluoperazine	117-89-5	-51.1763	-48.8986	-2.2777	11.5158
T1121	Podophyllotoxin	518-28-5	-51.1672	-50.6107	-0.5565	27.5088
T6880	Larotrectinib sulfate	1223405-08-0	-51.1671	-50.2526	-0.9146	7.1084
T0373	Erlotinib	183321-74-6	-51.1651	-51.3151	0.15	4.3567
T2122	Ixazomib	1072833-77-2	-51.1519	-51.1519	0	7.4904
T4117	Zuclopenthixol	53772-83-1	-51.1403	-51.4949	0.3546	8.5761
T3610	Ranitidin	66357-35-5	-51.1394	-48.7455	-2.3939	10.2745
T1207	Lovastatin	75330-75-5	-51.1067	-49.1904	-1.9163	18.931
T3623	Cobimetinib	934660-93-2	-51.0856	-48.1329	-2.9527	5.7863
T2212	Geniposide	24512-63-8	-51.0764	-48.9123	-2.1641	10.4747
T1653	O-(4-Hydroxy-3-iodophenyl)-3,5-diiodo-L-tyrosine	6893-02-3	-51.073	-49.676	-1.397	13.1462
T0444	Econazole	27220-47-9	-51.0704	-50.5499	-0.5205	13.0485
T0895	Oxytetracycline	79-57-2	-51.0399	-46.0966	-4.9433	27.1921
T0101	Doxazosin mesylate	77883-43-3	-51.0113	-51.3305	0.3192	18.7947
T2957	Arctigenin	7770-78-7	-50.9936	-50.9411	-0.0525	11.1513
T4538	palovarotene	410528-02-8	-50.9743	-45.9523	-5.022	8.122
T1170	Olopatadine hydrochloride	140462-76-6	-50.953	-43.0401	-7.9129	8.4662
T1408	Capecitabine	154361-50-9	-50.9498	-50.0145	-0.9354	8.5839
T0364L	Dibucaine hydrochloride	61-12-1	-50.9473	-48.4572	-2.49	10.8039
T1410	Torasemide	56211-40-6	-50.9315	-39.8034	-11.1281	11.7153
T7555	Piperidolate hydrochloride	129-77-1	-50.9304	-48.5915	-2.3388	7.9364

T6744	Methylbenactyzine Bromide	3166-62-9	-50.9265	-49.2699	-1.6566	15.3249
T8010	Dibutyl sebacate	109-43-3	-50.918	-49.3069	-1.6111	9.2364
T2296	Trelagliptin succinate	1029877-94-8	-50.8835	-47.7555	-3.128	10.9776
T1627	Famotidine	76824-35-6	-50.8815	-44.7433	-6.1381	5.9878
T6233	Entinostat	209783-80-2	-50.8645	-50.344	-0.5205	5.9088
T0771	Casanthranol	8024-48-4	-50.8561	-47.0169	-3.8392	27.6177
T0374	Sunitinib Malate	341031-54-7	-50.8514	-50.7081	-0.1434	10.9895
T0143	(??)-Bisoprolol hemifumarate	104344-23-2	-50.8377	-48.4958	-2.3419	16.0597
T1537	Rapamycin	53123-88-9	-50.816	-49.1758	-1.6402	26.3468
T2388	Nalfurafine hydrochloride	152658-17-8	-50.8004	-48.9202	-1.8802	16.9235
T2828	Spiculisporic Acid	469-77-2	-50.7955	-46.0214	-4.7741	10.055
T1552	Itopride hydrochloride	122892-31-3	-50.7689	-47.7107	-3.0582	6.7021
T1333	Tolazamide	1156-19-0	-50.7336	-40.0392	-10.6943	4.7607
T1398	Tadalafil	171596-29-5	-50.7183	-52.0771	1.3588	3.2808
T6956	Protirelin	24305-27-9	-50.6865	-49.333	-1.3535	13.3793
T6923	Ozanimod	1306760-87-1	-50.6837	-47.7022	-2.9815	4.6856
T1110	Meclizine dihydrochloride	1104-22-9	-50.6749	-31.6737	-19.0012	8.5816
T1484L	Perindopril erbumine	107133-36-8	-50.6562	-48.2983	-2.3579	20.9284
T7804	Garenoxacin mesylate hydrate	223652-90-2	-50.6491	-43.0776	-7.5715	23.2423
T3254	PHYTOL	150-86-7	-50.6371	-49.423	-1.2141	13.6287
T6020	Pacritinib	937272-79-2	-50.6343	-48.9571	-1.6771	6.2673
T0903	Stugeron	298-57-7	-50.6316	-47.9001	-2.7315	6.6885
T2885	Esculin	531-75-9	-50.6215	-47.6122	-3.0093	4.5016
T0464	Carbetapentane citrate	23142-01-0	-50.6103	-49.4387	-1.1716	13.7558
T0687	Simvastatin	79902-63-9	-50.5935	-51.2708	0.6773	23.5883
T1604	Tiratricol	51-24-1	-50.5842	-44.6734	-5.9109	13.761
T2385	Ipragliflozin	761423-87-4	-50.5512	-47.1323	-3.4189	5.899
T1594	Vecuronium bromide	50700-72-6	-50.5502	-49.6088	-0.9414	29.1318
T0031	Ziprasidone hydrochloride monohydrate	138982-67-9	-50.5477	-53.6344	3.0867	8.5753
T7839	Lasofoxifene Tartrate	190791-29-8	-50.5448	-48.5867	-1.9581	11.4299
T0435	Sulfinpyrazone	57-96-5	-50.52	-50.888	0.368	4.2515
T0230	Prasugrel	150322-43-3	-50.506	-50.5857	0.0797	13.4774
T0696	Naftopidil	57149-07-2	-50.478	-51.062	0.5841	18.8529
T1222	Trifluoperazine dihydrochloride	440-17-5	-50.4735	-29.4521	-21.0214	7.4777
T7148	JOSAMYCIN	16846-24-5	-50.4629	-51.6906	1.2277	34.1922
T2230	Paeoniflorin	23180-57-6	-50.445	-46.5291	-3.9159	10.9055
T1519	Rimonabant hydrochloride	158681-13-1	-50.4434	-50.2071	-0.2363	4.9362
T1446	Bambuterol hydrochloride	81732-46-9	-50.4409	-48.2812	-2.1597	19.3805
T0077	Dantrolene sodium salt	14663-23-1	-50.4392	-41.8079	-8.6314	3.1707
T2801	Aristolochic Acid	313-67-7	-50.4388	-49.247	-1.1918	8.4896
T1519L	Rimonabant	168273-06-1	-50.3915	-50.1505	-0.241	5.0434
T1651	Rebeprazole sodium	117976-90-6	-50.3853	-45.5254	-4.8599	9.8202
T2671	Regadenoson	313348-27-5	-50.381	-49.3131	-1.0678	3.4167
T1131	Ivermectin	70288-86-7	-50.3753	-49.8492	-0.5261	34.339
T1160	Fusidine	6990-06-3	-50.3687	-43.7071	-6.6617	20.1816
T4481	Tucidinostat	1616493-44-7	-50.3555	-49.6519	-0.7036	4.1542
T0369	Ethaverine hydrochloride	985-13-7	-50.3529	-50.0734	-0.2795	19.7797
T0299	Lactitol monohydrate	81025-04-9	-50.3341	-47.0528	-3.2812	7.6671

T2O2666	Methyl stearate	112-61-8	-50.3238	-50.6691	0.3453	8.564
T8482	Ripretinib	1442472-39-0	-50.3223	-47.8698	-2.4524	9.1948
T7412	Tandospirone citrate	112457-95-1	-50.3058	-47.9797	-2.3261	4.7487
T6485	Embelin	550-24-3	-50.3001	-39.2857	-11.0144	11.688
T0199	Cephradine	38821-53-3	-50.2871	-38.2456	-12.0415	7.4811
T4P2931	Linoleic acid	60-33-3	-50.2725	-42.175	-8.0975	7.0276
T7939	Fingolimod	162359-55-9	-50.2657	-33.6649	-16.6008	7.9055
T0156	Rocuronium bromide	119302-91-9	-50.2552	-49.9101	-0.3452	15.6886
T23133	Pentamidine	100-33-4	-50.2431	-32.5667	-17.6765	6.6429
T1193	Clomifene citrate	50-41-9	-50.2326	-46.9097	-3.3229	11.3992
T5495	Decloxizine dihydrochloride	13073-96-6	-50.2305	-30.6537	-19.5768	6.3122
T6963	Reboxetine mesylate	98769-84-7	-50.2304	-43.6738	-6.5566	11.5643
T6237	Trelagliptin	865759-25-7	-50.2277	-48.3132	-1.9145	10.0437
T2690	Tranilast	53902-12-8	-50.1951	-39.2778	-10.9173	9.1022
T4008	Metergoline	17692-51-2	-50.1849	-50.9033	0.7184	10.3072
T1419	Azithromycin dihydrate	117772-70-0	-50.1799	-49.6668	-0.5132	49.8909
T0364	Dibucaine	85-79-0	-50.1759	-47.4422	-2.7337	8.1057
T6952	Prasugrel Hydrochloride	389574-19-0	-50.154	-50.4245	0.2705	11.2998
T1495	Cefaclor monohydrate	70356-03-5	-50.1357	-38.4411	-11.6946	5.3795
T1174	Topotecan hydrochloride	119413-54-6	-50.1331	-46.7538	-3.3793	10.644
T2330	Rilpivirine	500287-72-9	-50.1083	-49.8572	-0.2511	8.6434
T0076	Paliperidone	144598-75-4	-50.1041	-39.7371	-10.3671	8.4753
T0378	Oxybuprocaine hydrochloride	5987-82-6	-50.0989	-46.7608	-3.3381	9.4284
T7808	Alacepril	74258-86-9	-50.0911	-46.5062	-3.5849	14.0065
T2970	Rotenone	83-79-4	-50.0715	-50.6694	0.5979	4.1993
T0799	Lactulose	4618-18-2	-50.0572	-46.4737	-3.5835	6.1576
T14495	Baloxavir	1985605-59-1	-50.0544	-45.1224	-4.932	60.6794
T0713	Drofenine hydrochloride	548-66-3	-50.0421	-48.857	-1.1851	17.6055
T1421	Aceclofenac	89796-99-6	-50.0415	-47.8526	-2.1889	11.0485
T0900	Cetylpyridinium chloride monohydrate	6004-24-6	-50.0402	-48.6155	-1.4246	9.3954
T1527	Gliclazide	21187-98-4	-49.9938	-39.8093	-10.1845	5.7479
T0226	Betaxolol hydrochloride	63659-19-8	-49.9706	-47.6737	-2.2969	4.0804
T6326	BMS345541	445430-58-0	-49.9706	-26.2397	-23.7309	2.0445
T6550	Isoconazole nitrate	24168-96-5	-49.9502	-50.0551	0.1049	6.9554
T6411	Biapenem	120410-24-4	-49.9181	-35.4315	-14.4866	2.6909
T1646	Famciclovir	104227-87-4	-49.9114	-48.7321	-1.1793	9.4187
T0166	Pancuronium bromide	15500-66-0	-49.8899	-49.8572	-0.0326	17.4981
T3465	Vesnarinone	81840-15-5	-49.8814	-48.1515	-1.7299	8.7747
T0031L	Ziprasidone hydrochloride	122883-93-6	-49.8585	-53.3082	3.4498	10.3711
T2P2810	Methyl Linoleate	112-63-0	-49.8343	-49.7161	-0.1182	6.451
T0937	Riboflavin	83-88-5	-49.8065	-47.6007	-2.2057	15.8931
T6307	Lubiprostone	136790-76-6	-49.804	-48.4627	-1.3414	12.6568
T6S1369	Vitexin	3681-93-4	-49.7923	-45.5943	-4.1981	12.7164
T0372	Gabexate mesylate	56974-61-9	-49.7884	-48.816	-0.9725	6.3708
T8070	Nonadecanoic acid	646-30-0	-49.7874	-43.6384	-6.149	10.5792
T0116	Balofloxacin	127294-70-6	-49.7732	-43.2427	-6.5305	21.8707
T3233	Sophoricoside	152-95-4	-49.7645	-48.9292	-0.8353	13.3911
T5171	Treprostinil Sodium	289480-64-4	-49.7403	-44.8431	-4.8972	8.6189

T2269	Pirinixic Acid	50892-23-4	-49.737	-42.4702	-7.2669	3.3526
T0380	Bicalutamide	90357-06-5	-49.7205	-49.4458	-0.2747	10.8737
T0038	Doxapram hydrochloride monohydrate	7081-53-0	-49.682	-49.6677	-0.0143	28.0565
T7952	Noscapine	128-62-1	-49.6812	-46.7121	-2.9691	12.9679
T0056	Oxeladin citrate	52432-72-1	-49.6784	-50.4269	0.7485	23.2155
T2231	17-Beta-Estradiol-3,17-Dipropionate	113-38-2	-49.6761	-48.5064	-1.1698	4.7619
T2401	Alogliptin Benzoate	850649-62-6	-49.6729	-45.8046	-3.8684	9.2714
T4999	Ertugliflozin	1210344-57-2	-49.6379	-46.8564	-2.7815	9.0671
T5032	Iguratimod	123663-49-0	-49.6372	-49.0503	-0.5868	7.1272
T0697	Aspartame	22839-47-0	-49.6123	-41.975	-7.6373	5.4066
T1169	Moguisteine	119637-67-1	-49.6026	-49.3822	-0.2203	9.7204
T6357	(S)-crizotinib	1374356-45-2	-49.6025	-50.3298	0.7273	14.4533
T6374	Alfacalcidol	41294-56-8	-49.5891	-48.8988	-0.6903	26.32
T0281	Meclocycline sulfosalicylate salt	73816-42-9	-49.5687	-44.2967	-5.272	21.471
T22459	Xanthinol Nicotinate	437-74-1	-49.5637	-46.8709	-2.6928	6.4626
T6686	Sulconazole Nitrate	61318-91-0	-49.5581	-49.3489	-0.2092	9.0879
T6192	Alogliptin	850649-61-5	-49.5567	-45.4444	-4.1123	11.18
T0320	Colchicine	64-86-8	-49.5436	-51.6539	2.1104	12.419
T7651	Rose Bengal	632-69-9	-49.541	-39.1958	-10.3453	0
T6482	Efaproxiral Sodium	170787-99-2	-49.5308	-45.4041	-4.1267	8.5019
T2806	Lappaconitine	32854-75-4	-49.5116	-41.4031	-8.1084	46.0855
T1936	Alectinib	1256580-46-7	-49.5078	-47.5532	-1.9546	8.5419
T5995	Larotrectinib	1223403-58-4	-49.5065	-48.4934	-1.0131	6.6403
T6439	Cetylpyridinium Chloride	123-03-5	-49.5053	-48.3201	-1.1852	8.3864
T1330	Acitretin	55079-83-9	-49.5006	-45.3399	-4.1607	14.9881
T1133	Nimesulide	51803-78-2	-49.4883	-48.9799	-0.5084	5.3311
T2481	Ascomycin	104987-12-4	-49.4767	-46.5916	-2.8851	26.1623
T4966	Ufenamate	67330-25-0	-49.4748	-48.443	-1.0318	5.5792
T0683	Mevastatin	73573-88-3	-49.4632	-48.6851	-0.7781	22.6096
T0982	(+)-Emetine dihydrochloride hydrate	7083-71-8	-49.46	-38.12	-11.34	19.3193
T4297	Flibanserin	167933-07-5	-49.4401	-50.9949	1.5548	9.7262
T0322	Fipexide	34161-24-5	-49.4365	-48.7059	-0.7306	10.8013
T6929	Pantoprazole sodium	138786-67-1	-49.403	-44.1606	-5.2424	7.5036
T0837	Furosemide	54-31-9	-49.3896	-48.3514	-1.0383	8.1082
T0095	Bendamustine hydrochloride	3543-75-7	-49.3886	-46.636	-2.7526	8.835
T1321	Nonivamide	2444-46-4	-49.3719	-45.1759	-4.196	6.2053
T8065	Tiamulin	55297-95-5	-49.3634	-48.9817	-0.3817	14.4581
T4718	Uridine 5'-monophosphate	58-97-9	-49.36	-34.8503	-14.5097	5.2457
T0912	Tetracycline hydrochloride	64-75-5	-49.3521	-46.2123	-3.1398	20.2896
T0423	Edetate?Trisodium	150-38-9	-49.3389	-36.4553	-12.8836	5.0104
T6213	Vinorelbine Tartrate	125317-39-7	-49.3389	-50.0087	0.6698	40.382
T0444L	Econazole nitrate	24169-02-6	-49.3101	-48.8774	-0.4327	8.042
T5004	Sodium Hyaluronate	9067-32-7	-49.3097	-48.1857	-1.124	5.6315
T1872	Fenretinide	65646-68-6	-49.3065	-47.7646	-1.5419	8.2468
T0197	Terazosin hydrochloride	63074-08-8	-49.268	-46.2915	-2.9765	9.2042
T2O2667	Methyl Oleate	112-62-9	-49.227	-49.1472	-0.0797	8.7075
T0017	Phenolphthalein	77-09-8	-49.2177	-45.461	-3.7567	8.0185
T2529	Zanamivir	139110-80-8	-49.2059	-46.2852	-2.9208	7.2526

T8387	Alectinib hydrochloride	1256589-74-8	-49.1933	-44.9702	-4.2231	6.6857
T3273	Bifendatatum	73536-69-3	-49.1769	-46.9243	-2.2526	9.41
T1175	Bromopride	4093-35-0	-49.1709	-47.2641	-1.9069	6.1692
T6447	Clindamycin	18323-44-9	-49.1488	-36.8977	-12.2511	14.4581
T0317	Buflomedil hydrochloride	35543-24-9	-49.1397	-49.1862	0.0465	6.9868
T1638	N-Acetylneuraminic acid	131-48-6	-49.1083	-45.0768	-4.0314	11.521
T8388	Vonoprazan	881681-00-1	-49.1027	-43.6172	-5.4855	16.4701
T3626	Acalabrutinib	1420477-60-6	-49.102	-45.8417	-3.2604	15.5857
T4462	Roflumilast N-oxide	292135-78-5	-49.1004	-44.694	-4.4064	7.1097
T0211L	Azelastine hydrochloride	79307-93-0	-49.0783	-47.0824	-1.9959	11.1505
T1551	Tegaserod maleate	189188-57-6	-49.0766	-47.3979	-1.6787	5.6109
T6875	Lesinurad	878672-00-5	-49.0762	-42.1419	-6.9344	7.1851
T1464	Toremifene citrate	89778-27-8	-49.0655	-46.4827	-2.5828	8.4805
T8385	Metoclopramide hydrochloride hydrate	54143-57-6	-49.0519	-47.3991	-1.6529	5.3649
T2025	Chidamide	743420-02-2	-49.0394	-48.1845	-0.8549	4.6745
T2404	Vonoprazan fumarate	1260141-27-2	-49.0325	-44.0463	-4.9862	9.6268
T0497	Cetrimonium bromide	57-09-0	-48.9903	-47.7144	-1.2759	9.4729
T1472	Trospium chloride	10405-02-4	-48.9835	-47.8161	-1.1674	11.0704
T0112	Diltiazem hydrochloride	33286-22-5	-48.9744	-46.1197	-2.8547	24.1423
T5570	Cephalotin acid	153-61-7	-48.9653	-41.5456	-7.4197	9.4551
T1654	Pentamidine isethionate	140-64-7	-48.9628	-45.6752	-3.2876	7.0234
T4121	Bicyclol	118159-48-1	-48.9612	-47.1995	-1.7617	9.6262
T2648	Pimecrolimus	137071-32-0	-48.9557	-49.7184	0.7627	37.3575
T0814L	Ampicillin	69-53-4	-48.9501	-36.5523	-12.3978	5.5648
T0119	Nitrendipine	39562-70-4	-48.9349	-49.5499	0.6149	18.0368
T1619	Trazodone hydrochloride	25332-39-2	-48.9309	-50.7959	1.8651	5.9762
T2317	Almotriptan Malate	181183-52-8	-48.882	-50.302	1.42	9.9305
T0586	Lactose	63-42-3	-48.8756	-44.6408	-4.2347	4.9111
T0239	Lonidamine	50264-69-2	-48.8747	-44.815	-4.0597	6.2566
T6414	Bisacodyl	603-50-9	-48.8742	-48.6867	-0.1875	10.6435
T0057	Famprofazone	22881-35-2	-48.8637	-48.1915	-0.6722	18.4582
T0111	Solifenacin succinate	242478-38-2	-48.8571	-46.6105	-2.2466	14.9669
T1277	Lincomycin hydrochloride	859-18-7	-48.8521	-43.5499	-5.3022	19.748
T6S1665	Irisflorentin	41743-73-1	-48.8425	-48.275	-0.5675	10.7387
T1061	Toltrazuril	69004-03-1	-48.8216	-48.0984	-0.7232	6.452
T2305	Isavuconazole	241479-67-4	-48.7961	-46.9251	-1.871	19.9788
T2590	Vismodegib	879085-55-9	-48.7897	-48.8427	0.053	6.9618
T2556	Nandrolone decanoate	360-70-3	-48.7854	-48.6491	-0.1363	6.2056
T1157	Moxidectin	113507-06-5	-48.7777	-48.3031	-0.4746	12.854
T1661	Crizotinib	877399-52-5	-48.776	-44.4893	-4.2867	14.1856
T0242	Sitagliptin	486460-32-6	-48.76	-46.158	-2.602	10.5909
T2394	Pixantrone dimaleate	144675-97-8	-48.7588	-45.6153	-3.1435	15.9966
T7013	Vorapaxar	618385-01-6	-48.7491	-48.0379	-0.7112	10.4914
T2508	Binimetinib	606143-89-9	-48.7416	-48.955	0.2133	4.8029
T0154	Nebivolol hydrochloride	152520-56-4	-48.717	-45.8116	-2.9054	5.0312
T0351	Risperidal	106266-06-2	-48.7161	-47.758	-0.958	23.6102
T1422	Nilvadipine	75530-68-6	-48.6869	-47.8809	-0.806	11.9015
T0810	Ethambutol dihydrochloride	1070-11-7	-48.6783	-24.032	-24.6463	7.5967

T2815	Puerarin	3681-99-0	-48.636	-45.008	-3.628	9.307
T2316	Omarigliptin	1226781-44-7	-48.6211	-26.9991	-21.622	12.4597
T1095	Sparfloxacin	110871-86-8	-48.6081	-44.0838	-4.5243	20.1071
T3098	Vorapaxar sulfate	705260-08-8	-48.6032	-48.7435	0.1403	13.9561
T1050	Prazosin hydrochloride	19237-84-4	-48.6019	-46.0033	-2.5986	14.06
T6386	Ampicillin sodium	69-52-3	-48.5961	-36.7013	-11.8948	6.9821
T6101	Entospletinib	1229208-44-9	-48.5714	-47.7293	-0.8421	5.6268
T1276	Danofloxacin mesylate	119478-55-6	-48.5274	-37.6774	-10.85	5.1876
T6096	Pretomanid	187235-37-6	-48.5224	-46.8851	-1.6373	4.6162
T0695	Avobenzone	70356-09-1	-48.5098	-45.5591	-2.9508	10.4596
T1293	Gatifloxacin	112811-59-3	-48.5093	-43.1308	-5.3784	19.5461
T3362	Eupatilin	22368-21-4	-48.5056	-45.8184	-2.6872	10.6167
T1656	Vandetanib	443913-73-3	-48.5041	-46.9935	-1.5106	26.999
T6552	Istradefylline	155270-99-8	-48.4995	-48.9672	0.4677	9.263
T2886	Aleuritic Acid	533-87-9	-48.4946	-41.2837	-7.2108	8.7601
T1852	Belinostat	414864-00-9	-48.4936	-43.2193	-5.2742	12.89
T7041	Donepezil	120014-06-4	-48.4852	-45.245	-3.2402	10.2608
T1687L	Doxycycline hyclate	24390-14-5	-48.4807	-34.7	-13.7807	22.748
T0691	Sulisobenzone	4065-45-6	-48.4573	-40.4627	-7.9945	5.939
T1069	Metoclopramide hydrochloride	7232-21-5	-48.4355	-47.5261	-0.9094	4.4667
T3276	Benorilate	5003-48-5	-48.4354	-48.6168	0.1814	5.8081
T1438	Butoconazole nitrate	64872-77-1	-48.4069	-48.4274	0.0204	10.5821
T1270	Vincristine sulfate	2068-78-2	-48.3753	-48.0367	-0.3385	59.7744
T6288	Mozavaptan	137975-06-5	-48.3629	-47.2081	-1.1548	20.0335
T1542	Pirenzepine dihydrochloride	29868-97-1	-48.3569	-47.944	-0.4129	18.3386
T0951	Hydroxychloroquine sulfate	747-36-4	-48.3432	-40.2551	-8.0881	4.6088
T8146	Imidocarb dipropionate	55750-06-6	-48.341	-39.4204	-8.9206	2.5998
T2P2923	Stearic acid	57-11-4	-48.3266	-40.7683	-7.5583	10.7919
T5784	Topotecan	123948-87-8	-48.3175	-49.19	0.8725	6.9991
T0254	Probucol	23288-49-5	-48.3068	-48.3064	-0.0004	48.3114
T8399	Crizotinib hydrochloride	1415560-69-8	-48.2845	-45.733	-2.5515	10.9738
T0458	Indometacin	53-86-1	-48.2802	-40.9712	-7.309	16.9986
T1583	Vorinostat	149647-78-9	-48.2798	-39.3645	-8.9153	4.3853
T0518	Methacycline hydrochloride	3963-95-9	-48.2263	-44.1383	-4.088	15.454
T6467	Dexrazoxane HCl	149003-01-0	-48.2204	-43.2978	-4.9227	6.0696
T0384	Estradiol benzoate	50-50-0	-48.2128	-48.1347	-0.0781	3.2156
T4616	5-Chloro-2-methoxyprocainamide	364-62-5	-48.1536	-46.419	-1.7346	5.3704
T0708	Oxaprozin	21256-18-8	-48.1503	-44.5209	-3.6294	4.5305
T1640	Ciprofloxacin	85721-33-1	-48.1431	-38.5172	-9.6258	7.6994
T4602	Hydrocortisone 21-hemisuccinate	2203-97-6	-48.143	-38.3283	-9.8147	14.6086
T2132	Buspirone hydrochloride	33386-08-2	-48.1408	-47.6023	-0.5385	13.1065
T0585	Maltitol	585-88-6	-48.1291	-43.2313	-4.8977	13.8681
T1077	Fluvoxamine maleate	61718-82-9	-48.0951	-45.2307	-2.8644	5.9999
T1304	Chlortetracycline hydrochloride	64-72-2	-48.0875	-48.3423	0.2549	23.4182
T4059	Selamectin	220119-17-5	-48.0851	-46.9192	-1.1658	18.9033
T5051	Isopropamide Iodide	71-81-8	-48.074	-47.7782	-0.2958	32.569
T8384	Gatifloxacin hydrochloride	121577-32-0	-48.0721	-42.5355	-5.5365	19.4042
T3441	Anlotinib	1058156-90-3	-48.0638	-43.5047	-4.5591	7.6386

T3145	D-(+)-Trehalose dihydrate	6138-23-4	-48.0457	-42.7977	-5.248	5.9948
T1012	Acebutolol hydrochloride	34381-68-5	-48.0242	-41.8333	-6.1909	8.4352
T1052	Proglumide	6620-60-6	-47.996	-39.12	-8.876	12.9684
T6680	Staurosporine	62996-74-1	-47.9934	-41.1888	-6.8046	11.2905
T6349	Sitafloxacin Hydrate	163253-35-8	-47.99	-41.2254	-6.7646	11.8672
T0990	Droperidol	548-73-2	-47.9835	-50.9604	2.9768	9.7121
T0457	Probenecid	57-66-9	-47.9723	-42.2501	-5.7222	11.4978
T1303	Auranofin	34031-32-8	-47.9438	-46.048	-1.8958	32.214
T8149	Dobutamine (hydrochloride)	49745-95-1	-47.9331	-43.6204	-4.3127	3.7547
T0957	Isradipine	75695-93-1	-47.933	-48.0652	0.1322	16.7946
T4985	Neticonazole Hydrochloride	130773-02-3	-47.929	-46.9173	-1.0117	5.4633
T1024	Roflumilast	162401-32-3	-47.8693	-42.7883	-5.081	5.2091
T1655	Ambrisentan	177036-94-1	-47.8612	-42.1369	-5.7243	35.6951
T0261	Amcinonide	51022-69-6	-47.8426	-47.4165	-0.4261	21.4016
T6406	Besifloxacin HCl	405165-61-9	-47.8352	-37.89	-9.9452	13.7956
T0268	Lobeline hydrochloride	134-63-4	-47.8293	-46.4041	-1.4252	16.1941
T7569	Clebopride malate	57645-91-7	-47.8291	-48.8125	0.9835	5.3593
T0163	Nisoldipine	63675-72-9	-47.8056	-47.4861	-0.3195	18.4731
T1066	Ketanserin	74050-98-9	-47.8027	-50.2763	2.4736	13.0888
T0475	Timolol Maleate	26921-17-5	-47.7989	-44.2707	-3.5282	5.713
T4093	Afobazole hydrochloride	173352-39-1	-47.758	-46.1281	-1.6299	8.001
T2527	Ulipristal acetate	126784-99-4	-47.7576	-47.3822	-0.3754	18.6753
T6241	Quetiapine Fumarate	111974-72-2	-47.7464	-45.6723	-2.0741	2.4699
T1235	Lincomycin hydrochloride monohydrate	7179-49-9	-47.7447	-35.4554	-12.2893	12.1566
T6448	Clindamycin HCl	21462-39-5	-47.6656	-47.6259	-0.0397	17.4354
T0146	Vagistat	65899-73-2	-47.6532	-47.4534	-0.1998	8.7896
T8364	Mebrofenin	78266-06-5	-47.6193	-42.4655	-5.1538	8.0286
T7503	Upadacitinib	1310726-60-3	-47.6172	-45.8398	-1.7774	10.1836
T5602	Benzylpenicillin potassium	113-98-4	-47.6045	-42.224	-5.3805	10.7646
T1282	Carteolol hydrochloride	51781-21-6	-47.6038	-44.9133	-2.6905	7.7835
T0139	Toremifene	89778-26-7	-47.5951	-45.5418	-2.0532	10.0578
T0331	Moxifloxacin hydrochloride	186826-86-8	-47.5933	-39.5789	-8.0145	20.3819
T0917	Sulfaquinoxaline sodium	967-80-6	-47.5841	-41.5953	-5.9888	2.4213
T3704	Lasmiditan	439239-90-4	-47.549	-43.9008	-3.6482	4.4447
T0265	Merbromin	129-16-8	-47.5415	-36.1833	-11.3582	9.8163
T8130	Ciprofloxacin hydrochloride hydrate	86393-32-0	-47.5209	-39.2741	-8.2468	8.3611
T2850	(+)-Bicuculline	485-49-4	-47.5128	-47.2678	-0.245	5.7984
T0826	Meloxicam	71125-38-7	-47.4688	-41.4464	-6.0225	5.7125
T3416	Aucubin	479-98-1	-47.4629	-45.4918	-1.9711	7.0218
T0485	Deflazacort	14484-47-0	-47.4563	-46.2463	-1.2099	28.8825
T0975	Chloroambucil	305-03-3	-47.4424	-42.4962	-4.9462	6.2186
T0203	Sumatriptan succinate	103628-48-4	-47.4411	-44.0068	-3.4343	5.8319
T0484	Deracoxib	169590-41-4	-47.4357	-46.2946	-1.1411	7.6387
T1306	Norfloxacin	70458-96-7	-47.4286	-37.618	-9.8107	8.0213
T8382	Cariprazine HCl	1083076-69-0	-47.428	-44.811	-2.617	9.8435
T1486	Oseltamivir phosphate	204255-11-8	-47.4094	-44.4832	-2.9262	9.4169
T0816	Acetohexamide	968-81-0	-47.3896	-35.6638	-11.7258	8.7378
T7807	Bendazac	20187-55-7	-47.3817	-38.756	-8.6258	4.3199

T1149	Fenofibrate	49562-28-9	-47.3757	-46.1993	-1.1764	7.8738
T5016	Tofogliflozin hydrate	1201913-82-7	-47.3369	-46.4599	-0.877	7.6568
T1734	Rabeprazole Related Compound E	117977-21-6	-47.3173	-46.0858	-1.2316	6.8358
T8123	Diazolidinyl urea	78491-02-8	-47.313	-43.9183	-3.3948	13.019
T5007	Benproperine phosphate	19428-14-9	-47.3051	-47.2711	-0.0341	8.9825
T8452	Dantrolene	7261-97-4	-47.2987	-41.93	-5.3687	3.9876
T0187	Amorolfine hydrochloride	78613-38-4	-47.2961	-45.2179	-2.0783	18.4388
T1179	Tiotropium bromide	136310-93-5	-47.2874	-48.254	0.9666	9.9679
T0377	Clofazimine	2030-63-9	-47.2723	-47.6551	0.3828	7.5582
T0912L	Tetracycline	60-54-8	-47.2462	-36.3338	-10.9124	20.405
T0255	Clomipramine hydrochloride	17321-77-6	-47.2363	-46.3971	-0.8391	7.872
T1389	Dyclonine hydrochloride	536-43-6	-47.2259	-46.0262	-1.1997	9.6677
T1274	Benzbromarone	3562-84-3	-47.2132	-46.903	-0.3101	2.5151
T6465	Dexamethasone Sodium Phosphate	55203-24-2	-47.178	-32.1854	-14.9927	11.4988
T2996	Tetrandrine	518-34-3	-47.1776	-45.8863	-1.2913	7.344
T5376	Aranidipine	86780-90-7	-47.1742	-46.6127	-0.5614	18.2802
T3629	Gamithromycin	145435-72-9	-47.1734	-46.7246	-0.4488	49.1177
T0181	Loteprednol etabonate	82034-46-6	-47.1714	-46.3642	-0.8072	24.3707
T1126	Penicillin V potassium salt	132-98-9	-47.1459	-43.3443	-3.8016	5.046
T3141	Bendazac L-lysine	81919-14-4	-47.1449	-38.8082	-8.3367	3.0621
T0292	Orbifloxacin	113617-63-3	-47.1315	-35.5524	-11.5791	13.0587
T0786	Clidinium bromide	3485-62-9	-47.1314	-44.4207	-2.7107	11.3031
T7560	Pivmecillinam hydrochloride	32887-03-9	-47.1215	-44.9389	-2.1826	15.6867
T0336	Benzalkonium chloride	8001-54-5	-47.112	-47.0262	-0.0857	12.8084
T6478	Donepezil HCl	120011-70-3	-47.1043	-37.689	-9.4153	10.8732
T0723	Cloperastine hydrochloride	14984-68-0	-47.0974	-46.7166	-0.3809	5.6987
T4986	Imidafenacin	170105-16-5	-47.0834	-46.455	-0.6284	24.616
T0222	Proparacaine hydrochloride	5875-06-9	-47.0788	-45.4348	-1.644	9.74
T0023	Valethamate bromide	90-22-2	-47.0771	-46.4041	-0.673	14.2199
T8358	Fenoterol	13392-18-2	-47.0684	-42.0087	-5.0597	6.5765
T6624	Oxytetracycline Dihydrate	6153-64-6	-47.0328	-46.4232	-0.6097	37.8157
T0266	Quinidine hydrochloride	6151-40-2	-47.02	-34.5382	-12.4818	13.4152
T1101	Minocycline hydrochloride	13614-98-7	-47.0137	-34.0512	-12.9625	18.2153
T1289	Sodium fusidate	751-94-0	-47.0134	-43.2561	-3.7573	27.6996
T1203	Nadolol	42200-33-9	-47.0043	-44.5247	-2.4796	10.156
T2588	Ivacaftor	873054-44-5	-47.001	-44.4666	-2.5344	55.6616
T3284	Carazolol	57775-29-8	-46.991	-44.2898	-2.7012	6.1278
T0981	Noscapine hydrochloride	912-60-7	-46.9868	-45.4735	-1.5132	11.2159
T6424	Bromfenac Sodium	91714-93-1	-46.9672	-45.6751	-1.2921	5.0557
T2387	Glycopyrrolate	596-51-0	-46.9351	-45.1047	-1.8304	17.5408
T6565	Levobetaxolol HCl	116209-55-3	-46.9335	-44.9017	-2.0317	7.5199
T5003	Rifamycin sodium salt	14897-39-3	-46.9329	-43.8319	-3.1011	35.6445
T8403	Cinacalcet	226256-56-0	-46.931	-46.8916	-0.0394	6.7772
T0250	Ciprofloxacin hydrochloride	93107-08-5	-46.9307	-37.464	-9.4667	6.5132
T0814	Ampicillin Trihydrate	7177-48-2	-46.9239	-36.8705	-10.0534	8.2732
T2758	Madecassic acid	18449-41-7	-46.9231	-42.8326	-4.0906	29.5666
T6864	Ixabepilone	219989-84-1	-46.923	-44.3543	-2.5687	20.8961
T1078	Sulfadimethoxine	122-11-2	-46.9219	-47.1314	0.2094	9.9674

T0460	Pramoxine hydrochloride	637-58-1	-46.8942	-44.4149	-2.4794	3.9914
T2406	Cinacalcet hydrochloride	364782-34-3	-46.8779	-46.7731	-0.1048	11.7547
T7941	Imipramine (hydrochloride)	113-52-0	-46.8757	-45.9624	-0.9133	5.98
T2756	Swertiamarin	17388-39-5	-46.8601	-45.9338	-0.9263	11.1692
T5832	Besifovir	441785-25-7	-46.8467	-46.4526	-0.3941	8.0626
T6638	Retapamulin	224452-66-8	-46.841	-44.6369	-2.204	12.3999
T1191	Analgin	5907-38-0	-46.8233	-37.6248	-9.1984	4.6454
T1617	Enrofloxacin	93106-60-6	-46.818	-42.3896	-4.4284	8.0763
T4094	Anlotinib Dihydrochloride	1360460-82-7	-46.8175	-42.8598	-3.9577	7.1563
T2392	Nafamostat mesylate	82956-11-4	-46.8145	-45.7774	-1.0371	4.8657
T8383	Cyclopentolate Hydrochloride	5870-29-1	-46.7955	-44.4548	-2.3408	15.5714
T1143	Carbenicillin disodium	4800-94-6	-46.7618	-37.4761	-9.2857	10.7419
T1091	Chlortalidone	77-36-1	-46.7539	-46.2314	-0.5225	8.9736
T6602	Naratriptan	143388-64-1	-46.7368	-43.9298	-2.807	13.9589
T5000	Diflucortolone valerate	59198-70-8	-46.7321	-45.8065	-0.9255	18.0872
T2731	Usnic Acid	125-46-2	-46.712	-45.7554	-0.9566	11.7286
T0142	Brinzolamide	138890-62-7	-46.7071	-45.5335	-1.1736	3.4857
T0073	Octyl 4-methoxycinnamate	5466-77-3	-46.7061	-46.2912	-0.4149	8.8339
T14856	Calcipotriol	112965-21-6	-46.6922	-46.4247	-0.2675	11.1427
T6539	Ibandronate sodium monohydrate	138926-19-9	-46.6888	-45.145	-1.5438	5.2213
T1172	Diclazuril	101831-37-2	-46.6852	-44.8958	-1.7894	15.6631
T0794	Mepenzolate Bromide	76-90-4	-46.6655	-44.5289	-2.1366	9.7032
T2938	Polydatin	65914-17-2	-46.6523	-43.4732	-3.179	8.9459
T2S0157	Methyl hexadecanoate	112-39-0	-46.6467	-46.8853	0.2387	10.5637
T1273	Diacerein	13739-02-1	-46.6135	-46.2493	-0.3642	28.7558
T0391	Linezolid	165800-03-3	-46.6081	-44.6407	-1.9674	4.9689
T1460	Dicyclomine hydrochloride	67-92-5	-46.6027	-44.6058	-1.9969	19.5641
T1599L	Ibandronate sodium	138844-81-2	-46.599	-34.0941	-12.5049	8.254
T1399	Sucralose	56038-13-2	-46.5634	-42.3963	-4.1671	18.4779
T4577	Cariprazine	839712-12-8	-46.5496	-46.9199	0.3703	16.836
T8083	Pyridoxal 5-phosphate monohydrate	41468-25-1	-46.512	-31.7464	-14.7655	4.9916
T0396	Pipemidic acid	51940-44-4	-46.4938	-38.0108	-8.483	8.1877
T6561	Laquinimod	248281-84-7	-46.4898	-40.7382	-5.7516	13.3745
T2403	Bromfenac sodium	120638-55-3	-46.4845	-45.2904	-1.1941	5.1303
T1320	Disopyramide	3737-09-5	-46.483	-45.9274	-0.5556	27.2326
T2O2668	Oleic acid	112-80-1	-46.4718	-44.2946	-2.1772	8.8116
T2S1865	Octyl gallate	1034-01-1	-46.4674	-45.0979	-1.3695	4.388
T3684	CP21R7	125314-13-8	-46.4656	-45.5784	-0.8872	3.0393
T6242	Rupatadine Fumarate	182349-12-8	-46.4573	-49.3671	2.9098	10.2355
T6651	Safinamide mesylate	202825-46-5	-46.4484	-44.2822	-2.1662	6.3234
T12643	(R)-Terazosin	109351-34-0	-46.4075	-46.2557	-0.1519	13.9212
T2880	D-Cellobiose	528-50-7	-46.3226	-42.6491	-3.6736	11.9977
T0172	Maprotiline hydrochloride	10347-81-6	-46.3147	-43.496	-2.8187	9.0615
T2398	Tofacitinib Citrate	540737-29-9	-46.2997	-44.5581	-1.7416	19.6563
T1497	Liranaftate	88678-31-3	-46.2856	-45.6609	-0.6247	3.7114
T1402	Fenofibric acid	42017-89-0	-46.2557	-42.6276	-3.6281	14.2971
T1668	Vinblastine sulfate	143-67-9	-46.2458	-46.19	-0.0559	50.8035
T3232	Higenamine?Hydrochloride	11041-94-4	-46.2346	-41.8835	-4.3511	2.481

T4567	Sulfaphenazole	526-08-9	-46.2262	-46.7502	0.524	4.58
T5077	Sodium Desoxycholate	302-95-4	-46.2247	-40.0016	-6.2231	20.0921
T1625	Lomefloxacin hydrochloride	98079-52-8	-46.2129	-38.7639	-7.449	13.5033
T0025	Haloperidol	52-86-8	-46.2061	-46.3406	0.1346	17.992
T1577	Midodrine hydrochloride	3092-17-9	-46.2048	-41.4462	-4.7586	5.8963
T1392	Fluorescein	2321-07-5	-46.2047	-44.2572	-1.9475	0.057
T0717	Enoxacin Sesquihydrate	84294-96-2	-46.1966	-38.219	-7.9776	3.9713
T4563	Thonzylamine	91-85-0	-46.1918	-44.724	-1.4678	7.0748
T6587	Mitiglinide Calcium	145525-41-3	-46.1916	-39.7702	-6.4214	14.2187
T0809	Dicumarol	66-76-2	-46.159	-37.654	-8.505	7.702
T6905	Netupitant	290297-26-6	-46.1447	-45.6374	-0.5073	82.6367
T0847	Chenodeoxycholic acid	474-25-9	-46.143	-39.5489	-6.5942	19.6858
T1097	Loratadine	79794-75-5	-46.1356	-45.9117	-0.2239	13.7256
T2773	Vindoline	2182-14-1	-46.1267	-45.8699	-0.2568	41.1936
T1563	Nifuroxazide	965-52-6	-46.0966	-44.3851	-1.7115	2.4238
T0726	Benzydamine hydrochloride	132-69-4	-46.0807	-44.5187	-1.5619	5.5132
T6684	Succinylcholine Chloride Dihydrate	6101-15-1	-46.0537	-45.1473	-0.9064	9.2275
T1191L	Dipyron	68-89-3	-46.048	-37.806	-8.242	5.0983
T1822L	Clemizole hydrochloride	1163-36-6	-46.0455	-43.5569	-2.4886	6.4779
T1232	Pyrilamine maleate	59-33-6	-46.016	-43.5692	-2.4469	9.9959
T0183	Pramiracetam	68497-62-1	-46.0054	-45.1032	-0.9021	11.9878
T3941	Choline Fenofibrate	856676-23-8	-46.0023	-42.3092	-3.6931	10.8034
T6630	Quercetin Dihydrate	6151-25-3	-45.9943	-41.0373	-4.957	5.3755
T1822	Clemizole	442-52-4	-45.9803	-43.9288	-2.0515	6.935
T6321	Tofacitinib	477600-75-2	-45.977	-44.1735	-1.8035	16.8969
T1466	Nadifloxacin	124858-35-1	-45.9646	-41.9436	-4.021	7.3906
T0208	Prednisolone phosphate sodium	125-02-0	-45.955	-32.8024	-13.1526	15.9925
T2A2509	Laurocapram	59227-89-3	-45.9121	-45.3556	-0.5565	10.2736
T2686L	Esomeprazole Sodium	161796-78-7	-45.8923	-37.7473	-8.1451	9.5134
T1154	Rifaximin	80621-81-4	-45.8889	-43.2503	-2.6387	30.5999
T0327	Fendiline hydrochloride	13636-18-5	-45.8804	-43.6087	-2.2718	9.0367
T1672	Dimesna	16208-51-8	-45.8787	-33.1243	-12.7545	4.5488
T0995	Clorpyrifos	2921-88-2	-45.8511	-44.4535	-1.3977	3.3978
T3824	Jaceosidin	18085-97-7	-45.8379	-44.3415	-1.4964	8.2842
T0815L	Warfarin sodium	129-06-6	-45.8179	-46.1174	0.2995	16.2727
T6606	Nicaraven	79455-30-4	-45.8174	-44.5367	-1.2807	2.6288
T6892	Niraparib tosylate	1038915-73-9	-45.8136	-32.1281	-13.6855	9.5915
T1092	Zolmitriptan	139264-17-8	-45.8091	-43.9846	-1.8245	4.3513
T6381	Amifostine trihydrate	112901-68-5	-45.8091	-23.394	-22.4151	3.3525
T1503	Esmolol hydrochloride	81161-17-3	-45.8012	-45.6696	-0.1316	7.901
T0862	Diphenidol hydrochloride	3254-89-5	-45.7733	-45.4133	-0.36	11.5197
T7902	Ozenoxacin	245765-41-7	-45.766	-42.3866	-3.3794	13.771
T1045	Trimipramine maleate	521-78-8	-45.7628	-45.5372	-0.2257	17.361
T0302	Triflupromazine hydrochloride	1098-60-8	-45.7506	-43.3613	-2.3893	9.8418
T6906	Tamoxifen	10540-29-1	-45.7254	-42.8574	-2.8679	9.9076
T6493	Escitalopram Oxalate	219861-08-2	-45.7229	-43.3634	-2.3595	9.8689
T1468	Lornoxicam	70374-39-9	-45.7205	-45.4382	-0.2823	4.0559
T6461	Dapoxetine HCl	129938-20-1	-45.7069	-44.1996	-1.5072	6.9222

T1444	Lomefloxacin	98079-51-7	-45.7013	-38.908	-6.7933	12.5764
T0584L	D-(+)-Maltose monohydrate	6363-53-7	-45.6966	-42.4708	-3.2257	7.5425
T4537	SodiumCholate	361-09-1	-45.6889	-40.1401	-5.5488	21.9235
T6504	Flupirtine maleate	75507-68-5	-45.682	-44.0058	-1.6762	10.09
T0099	Tolterodine tartrate	124937-52-6	-45.6799	-45.4686	-0.2112	17.0775
T0466	Celecoxib	169590-42-5	-45.6763	-44.7885	-0.8878	4.8926
T4510	Polaprezinc	107667-60-7	-45.6753	-30.9824	-14.6929	5.7025
T6S0659	Rhynchophylline	76-66-4	-45.6728	-47.081	1.4082	8.6793
T4548	Procaterol hydrochloride	81262-93-3	-45.6683	-43.6943	-1.974	14.0119
T1163	Betamethasone 17-valerate	2152-44-5	-45.6579	-44.502	-1.1558	29.2188
T2174	Quercetin	117-39-5	-45.6477	-40.5954	-5.0523	3.4032
T7363	Ibuprofen Piconol	64622-45-3	-45.6255	-45.7126	0.0871	14.4568
T0836	Sulfadoxine	2447-57-6	-45.6162	-47.1716	1.5554	5.4477
T0393	Anastrozole	120511-73-1	-45.6021	-45.3274	-0.2747	12.5485
T1054	Tolbutamide	64-77-7	-45.5996	-36.0242	-9.5754	5.1129
T3067	Tideglusib	865854-05-3	-45.5985	-45.6923	0.0938	10.5794
T0450	Fluoxetine	54910-89-3	-45.5868	-42.1292	-3.4576	3.4305
T3500	Flurbiprofen Axetil	91503-79-6	-45.5714	-45.5953	0.0239	9.8068
T4269	Buparvaquone	88426-33-9	-45.5666	-42.8009	-2.7657	17.5704
T3428	Helicid	80154-34-3	-45.5507	-40.5622	-4.9885	6.5233
T1649	Tenofovir	147127-20-6	-45.5182	-40.88	-4.6382	4.1108
T5017	Nimustine Hydrochloride	55661-38-6	-45.5179	-45.5575	0.0396	13.1204
T6089	Neflamapimod	209410-46-8	-45.5142	-45.0656	-0.4487	5.2768
T0717L	Enoxacin	74011-58-8	-45.5137	-35.9765	-9.5372	10.4664
T2836	Isorhamnetin	480-19-3	-45.511	-40.1242	-5.3868	5.2851
T0970	Anisodamine	17659-49-3	-45.4927	-43.7414	-1.7513	10.1495
T0219	Valdecoxib	181695-72-7	-45.4723	-45.3009	-0.1714	4.1945
T6112	Doxercalciferol	54573-75-0	-45.4698	-45.3559	-0.1139	27.7636
T0450L	Fluoxetine hydrochloride	56296-78-7	-45.467	-41.8976	-3.5694	3.8277
T2780	Catalpol	2415-24-9	-45.4593	-42.3243	-3.135	12.4192
T0584	Maltose	69-79-4	-45.4516	-41.8791	-3.5725	6.4743
T3578	Pyridoxal phosphate	54-47-7	-45.3914	-31.7391	-13.6524	2.9868
T0818	Decamethonium bromide	541-22-0	-45.3782	-44.9255	-0.4527	12.261
T2946	Tanshinone IIA sulfonate sodium	69659-80-9	-45.3687	-39.5638	-5.8049	1.0512
T0852	Carbazochrome sodium sulfonate	51460-26-5	-45.3462	-33.4243	-11.9219	6.8318
T2131	Articaine HCl	23964-57-0	-45.3068	-44.4265	-0.8803	4.4275
T0506	Clotrimazole	23593-75-1	-45.3059	-45.4992	0.1932	31.0925
T1526	Nifuratel	4936-47-4	-45.2913	-43.2071	-2.0843	4.1083
T1471	Duloxetine hydrochloride	136434-34-9	-45.2851	-43.9252	-1.3599	6.9157
T1334	Hydroquinidine	1435-55-8	-45.2792	-43.3148	-1.9644	15.8677
T4911	alpha-Arbutin	84380-01-8	-45.2588	-42.7015	-2.5573	3.1971
T2216	Entacapone	130929-57-6	-45.2334	-43.8063	-1.4271	11.841
T0263	Chloropyramine hydrochloride	6170-42-9	-45.19	-42.9303	-2.2597	6.7958
T2686	Esomeprazole Magnesium	161973-10-0	-45.1869	-41.3667	-3.8202	5.6626
T8398	Lasmiditan hydrochloride	613677-28-4	-45.178	-45.4865	0.3085	8.2126
T4313	Tinoridine hydrochloride	25913-34-2	-45.1777	-43.6089	-1.5688	7.9802
T6244	Cidofovir	113852-37-2	-45.1757	-41.6474	-3.5283	6.9425
T0188	Fluticasone propionate	80474-14-2	-45.1683	-43.7329	-1.4354	19.083

T1531	Mometasone furoate	83919-23-7	-45.1494	-42.7581	-2.3913	30.2993
T3231	Niraparib	1038915-60-4	-45.1413	-31.0871	-14.0542	6.8185
T6002	Enzalutamide	915087-33-1	-45.1196	-44.4436	-0.676	17.364
T8666	Naratriptan	121679-13-8	-45.1108	-42.2491	-2.8616	18.546
T0147	Clemastine fumarate	14976-57-9	-45.1107	-42.1045	-3.0062	14.876
T6710	Troxipide	30751-05-4	-45.1107	-31.5134	-13.5973	8.1996
T4273	Furagin	1672-88-4	-45.1016	-42.6196	-2.482	4.9341
T4439	Choline glycerophosphate	28319-77-9	-45.0932	-37.2228	-7.8704	4.994
T1829	Ruxolitinib	941678-49-5	-45.0833	-44.9643	-0.119	5.8966
T1384	Chlorpromazine hydrochloride	69-09-0	-45.0793	-46.3819	1.3026	7.6593
T0835	Tamoxifen Z-isomer citrate	54965-24-1	-45.0739	-40.9451	-4.1289	10.0311
T1708	Pimobendan	74150-27-9	-45.0726	-43.0554	-2.0172	5.9421
T0338	Minaprine dihydrochloride	25905-77-5	-45.0658	-44.8589	-0.2069	8.1879
T1704	Diosmetin	520-34-3	-45.0446	-39.0769	-5.9677	4.7388
T2717	Salidroside	10338-51-9	-45.0337	-42.6363	-2.3974	5.0322
T2326	Tolvaptan	150683-30-0	-45.0302	-44.0219	-1.0082	13.1058
T6156	S-Ruxolitinib	941685-37-6	-44.9847	-43.8599	-1.1248	10.0193
T1433	Ritodrine hydrochloride	23239-51-2	-44.9827	-41.8645	-3.1182	5.1198
T1294	Fleroxacin	79660-72-3	-44.9826	-37.437	-7.5456	13.0652
T1674	Nateglinide	105816-04-4	-44.9821	-47.5867	2.6046	10.3979
T7938	Quinidine	56-54-2	-44.9777	-42.7346	-2.2431	10.8412
T6687	Suprofen	40828-46-4	-44.9667	-37.7571	-7.2096	8.7313
T0824	Nonoxynol	26027-38-3	-44.9404	-44.0107	-0.9297	7.2661
T2759	Loganin	18524-94-2	-44.9285	-41.7783	-3.1502	7.7084
T7811	Pilsicainide HCl	88069-49-2	-44.9228	-43.5908	-1.332	14.3625
T0365	Thioridazine hydrochloride	130-61-0	-44.9027	-36.327	-8.5757	15.7518
T0991	Desipramine hydrochloride	58-28-6	-44.8974	-43.106	-1.7914	6.192
T6890	Mitomycin C	50-07-7	-44.8926	-43.7649	-1.1277	10.8071
T1517	Benserazide hydrochloride	14919-77-8	-44.8727	-32.4627	-12.41	2.5692
T1186	Ifenprodil Tartrate	23210-58-4	-44.8625	-44.4654	-0.3971	10.0259
T6502	Fluocinonide	356-12-7	-44.8187	-44.3498	-0.4689	17.4402
T1574	Etoricoxib	202409-33-4	-44.7968	-44.5472	-0.2496	4.9652
T3996	Dapiprazole Hydrochloride	72822-13-0	-44.7696	-46.8073	2.0377	6.7922
T0483	Sotalol hydrochloride	959-24-0	-44.7598	-43.4075	-1.3523	4.2826
T1483	Citalopram HBr	59729-32-7	-44.7575	-42.8041	-1.9534	11.1265
T4573	Cyclothiazide	2259-96-3	-44.7439	-45.3451	0.6013	10.4066
T0405	Hexamethylenetetramine	100-97-0	-44.7307	-9.9633	-34.7674	0
T4463	Rucaparib	283173-50-2	-44.7254	-41.6985	-3.0269	1.7018
T3043	Ruxolitinib (INCB-18424) phosphate	1092939-17-7	-44.7223	-44.8115	0.0891	8.5679
T3272	Metamizole magnesium	63372-86-1	-44.715	-39.0677	-5.6473	5.7598
T4561	Tiaprofenic acid	33005-95-7	-44.6963	-37.7188	-6.9774	2.9668
T1611	Isotretinoin	4759-48-2	-44.688	-39.2359	-5.452	8.3208
T0785	Dexchlorpheniramine Maleate	2438-32-6	-44.6752	-42.309	-2.3662	6.1786
T3117	Marbofloxacin	115550-35-1	-44.6585	-38.293	-6.3655	1.7331
T1237	Azathioprine	446-86-6	-44.6576	-44.6712	0.0136	1.9179
T1400	Demeclocycline hydrochloride	64-73-3	-44.6549	-39.3676	-5.2873	34.0458
T1559	Betamethasone dipropionate	5593-20-4	-44.6496	-43.9414	-0.7082	27.7101
T2551	Etravirine	269055-15-4	-44.6154	-44.3516	-0.2638	10.2093

T5186	Oseltamivir acid	187227-45-8	-44.6016	-24.6815	-19.9201	10.9337
T0758	Piroxicam	36322-90-4	-44.5963	-39.0985	-5.4978	2.3498
T0797	Cimetidine	51481-61-9	-44.5934	-43.7459	-0.8474	3.9805
T0490	Chlorpropamide	94-20-2	-44.5811	-37.0942	-7.4869	3.1923
T1530	Mitiglinide calcium	207844-01-7	-44.5771	-42.503	-2.0742	10.676
T0094	Budesonide	51333-22-3	-44.5624	-43.3592	-1.2032	25.8464
T6127	Rucaparib Phosphate	459868-92-9	-44.5545	-41.3208	-3.2337	2.4792
T1280	Nepafenac	78281-72-8	-44.5482	-43.8716	-0.6766	5.3515
T4553	Procaine penicillin G	54-35-3	-44.5475	-34.4797	-10.0678	6.5647
T2764	10-Hydroxycamptothecin	19685-09-7	-44.5264	-43.4088	-1.1176	3.8251
T0178	Saxagliptin hydrate	945667-22-1	-44.507	-28.9683	-15.5387	8.5501
T1521	Hydroxy Camptothecine	64439-81-2	-44.4929	-43.3815	-1.1114	3.7525
T3979	Emedastine	87233-61-2	-44.488	-43.3175	-1.1705	12.1018
T2908	Palmitic acid	57-10-3	-44.4796	-39.6904	-4.7891	8.7477
T3282	Trimeprazine tartrate	4330-99-8	-44.4666	-45.101	0.6344	7.2197
T1546	Nitazoxanide	55981-09-4	-44.4502	-42.0031	-2.4471	4.0728
T2184L	Sulfamethazine sodium salt	1981-58-4	-44.4475	-39.754	-4.6934	4.8052
T2565	Hesperetin	520-33-2	-44.4471	-41.9948	-2.4522	7.6957
T4982	Benznidazole	22994-85-0	-44.4387	-43.2647	-1.1741	2.8113
T4952	10-Hydroxycampothecin	67656-30-8	-44.4203	-43.922	-0.4983	3.7602
T8386	Esomeprazole Magnesium trihydrate	217087-09-7	-44.4181	-38.0387	-6.3794	7.6625
T2549	Robenidine hydrochloride	25875-50-7	-44.3874	-42.5947	-1.7927	4.8195
T3949	Myristic acid	544-63-8	-44.3791	-37.6583	-6.7208	6.0521
T2526	Ciclesonide	126544-47-6	-44.3758	-43.7379	-0.638	20.6677
T2745	Glycyrrhetinic acid	1449-05-4	-44.3749	-40.3617	-4.0132	11.908
T6316	Calcitriol	32222-06-3	-44.3703	-44.4369	0.0666	21.4599
T1115	Doxylamine succinate	562-10-7	-44.3505	-42.4807	-1.8698	12.4929
T0253	Methscopolamine	155-41-9	-44.2968	-43.3062	-0.9907	5.9422
T6708	Tolcapone	134308-13-7	-44.292	-43.7538	-0.5382	3.7452
T0168	Depofemin	313-06-4	-44.2883	-43.8664	-0.4219	5.2218
T1102	Mifepristone	84371-65-3	-44.279	-44.3385	0.0596	11.5339
T1432	Phenylbutazone	50-33-9	-44.2749	-44.9133	0.6384	8.4785
T4545	Propacetamol Hydrochloride	66532-86-3	-44.2648	-42.3366	-1.9282	7.4941
T7469	Alendronic Acid	66376-36-1	-44.2521	-29.6439	-14.6083	8.8299
T1544	Olsalazine disodium	6054-98-4	-44.2514	-33.0105	-11.2409	2.8192
T6758	Anisomycin	22862-76-6	-44.2338	-41.822	-2.4117	5.1254
T0902	Ofloxacin	82419-36-1	-44.2119	-37.5484	-6.6635	2.0345
T2782	Catharanthine	2468-21-5	-44.2074	-42.6151	-1.5922	8.186
T0007	Atenolol	29122-68-7	-44.1984	-42.6524	-1.546	2.4133
T1318	Ouabain octahydrate	11018-89-6	-44.1902	-42.5935	-1.5967	32.5278
T4566	Lodoxamide	53882-12-5	-44.1765	-38.8972	-5.2792	2.3076
T0182L	(S)-(+)-Clopidogrel hydrogen sulfate	120202-66-6	-44.1559	-42.3065	-1.8494	7.0042
T0454L	Florfenicol	73231-34-2	-44.1498	-41.8463	-2.3035	7.0571
T0958	Penicillin G sodium salt	69-57-8	-44.1458	-36.32	-7.8258	7.387
T2405	Revaprazan hydrochloride	178307-42-1	-44.1379	-43.5178	-0.6201	8.7179
T6696	Tazarotene	118292-40-3	-44.1201	-43.7603	-0.3598	1.8152
T1586	Tenoxicam	59804-37-4	-44.1133	-38.944	-5.1693	4.1949
T2181	Scopolamine N-oxide HBr	6106-81-6	-44.1077	-40.1612	-3.9465	8.2071

T8069	Triethyl citrate	77-93-0	-44.0977	-42.4911	-1.6066	11.4104
T6713	Ulipristal	159811-51-5	-44.0863	-44.5736	0.4873	17.5759
T8413	Lasmiditan succinate	439239-92-6	-44.0741	-42.7251	-1.3491	7.9827
T0773	Febuxostat	144060-53-7	-44.0272	-42.4437	-1.5835	5.3174
T1747	Torezolid	856866-72-3	-44.0261	-42.9228	-1.1033	5.3783
T2112	Fexinidazole	59729-37-2	-44.0188	-43.489	-0.5298	6.9028
T0425	Pefloxacin Mesylate	70458-95-6	-44.0158	-36.2971	-7.7187	5.618
T3262	Creatine phosphate disodium salt	922-32-7	-44.012	-27.2831	-16.729	4.5033
T1072	Loxeen	6856-31-1	-43.9941	-44.4864	0.4923	10.5045
T1212	Ketorolac tromethamine salt	74103-07-4	-43.9392	-39.8105	-4.1287	5.8836
T2587	Ecabet Sodium	86408-72-2	-43.9221	-41.377	-2.545	20.2524
T4560	Alprenolol	13655-52-2	-43.8962	-43.2612	-0.635	4.9793
T6616	Ondansetron HCl	99614-01-4	-43.8929	-44.2468	0.3539	12.532
T0425L	Pefloxacin Mesylate Dihydrate	149676-40-4	-43.8644	-36.7399	-7.1245	5.2744
T2569	Chlorotrianisene	569-57-3	-43.8624	-43.0629	-0.7996	11.8938
T0459	Sulindac	38194-50-2	-43.8591	-37.8004	-6.0587	6.6683
T7002	Trihexyphenidyl hydrochloride	52-49-3	-43.8556	-42.9381	-0.9174	12.0495
T1116	D-Biotin	58-85-5	-43.8539	-38.1004	-5.7535	2.6392
T1478	Ondansetron hydrochloride	103639-04-9	-43.8521	-44.7365	0.8844	27.0991
T2592	Ropinirole hydrochloride	91374-20-8	-43.8291	-42.253	-1.5761	5.7139
T0345	Estropipate	7280-37-7	-43.8274	-40.7621	-3.0653	2.4996
T0860	Mefloquine hydrochloride	51773-92-3	-43.8196	-26.9309	-16.8887	11.6017
T1805	Ataluren	775304-57-9	-43.8196	-37.7118	-6.1078	2.5133
T5013	Brequinar	96187-53-0	-43.7967	-39.382	-4.4147	7.3145
T4564	Ethacrynic acid	58-54-8	-43.7781	-39.0663	-4.7118	8.0149
T6964	Resiquimod	144875-48-9	-43.7629	-43.89	0.1272	9.0648
T1188	Mizoribine	50924-49-7	-43.7482	-42.5335	-1.2147	5.5104
T1209	Flunixin meglumin	42461-84-7	-43.719	-41.2152	-2.5038	11.0266
T0324	Isoxsuprine hydrochloride	579-56-6	-43.7187	-41.0904	-2.6283	10.3226
T1512	Rizatriptan benzoate	145202-66-0	-43.7135	-42.1324	-1.5811	2.1329
T0859	Fenbufen	36330-85-5	-43.7107	-38.1094	-5.6014	4.5959
T1415	Gemfibrozil	25812-30-0	-43.7045	-38.5952	-5.1093	9.1495
T1550	Thiamphenicol	15318-45-3	-43.6988	-42.9882	-0.7106	4.6613
T0844	Sulfamethoxypyridazine	80-35-3	-43.6961	-43.0263	-0.6698	5.8196
T1205	Chloramphenicol	56-75-7	-43.6918	-42.3367	-1.3552	6.2557
T1027	Luteolin	491-70-3	-43.6901	-38.1451	-5.5451	5.9872
T1030	Triprolidine hydrochloride	6138-79-0	-43.686	-41.5061	-2.1799	4.0747
T2395	Vortioxetine	508233-74-7	-43.6818	-42.1273	-1.5546	5.1335
T2835	Morin	480-16-0	-43.6755	-37.5431	-6.1324	6.6655
T1382	Griseofulvin	126-07-8	-43.6656	-44.5145	0.8488	5.3559
T6649	S- (+)-Rolipram	85416-73-5	-43.658	-43.2199	-0.4381	7.8516
T0699	Sulfamonomethoxine	1220-83-3	-43.6507	-44.3942	0.7434	4.6183
T0098	Ipratropium bromide monohydrate	66985-17-9	-43.6342	-41.5678	-2.0664	8.3493
T3285	Eslicarbazepine Acetate	236395-14-5	-43.6242	-43.4774	-0.1468	9.3611
T0428	Alverine citrate	5560-59-8	-43.582	-43.2658	-0.3162	6.8125
T0812	Propranolol hydrochloride	318-98-9	-43.5726	-41.4863	-2.0863	3.9966
T8696	Propranolol hydrochloride	3506-09-0	-43.5726	-41.4863	-2.0863	3.9966
T1540	Doxepin hydrochloride	1229-29-4	-43.5706	-43.2116	-0.359	6.7199

T0869	Atomoxetine hydrochloride	82248-59-7	-43.564	-39.8841	-3.6799	7.0723
T2142	Yohimbine hydrochloride	65-19-0	-43.5549	-46.7883	3.2334	2.9283
T1620	Pazufloxacin Mesylate	163680-77-1	-43.5449	-34.5267	-9.0182	8.2718
T1720	Amfenac Sodium Monohydrate	61618-27-7	-43.5401	-42.0546	-1.4854	5.4034
T1141	Fenbendazole	43210-67-9	-43.5195	-43.372	-0.1475	4.8666
T0443	Salicoside	138-52-3	-43.5167	-41.6905	-1.8262	4.7026
T0839	Ketoprofen	22071-15-4	-43.5152	-43.1141	-0.4012	5.5367
T0685	Retinyl acetate	127-47-9	-43.5102	-43.2468	-0.2634	13.0209
T6618	Orphenadrine Citrate	4682-36-4	-43.5095	-42.6359	-0.8735	6.2585
T6567	Levofloxacin	100986-85-4	-43.499	-40.6161	-2.8829	1.8423
T2183	Scopolamine butylbromide	149-64-4	-43.4981	-41.6471	-1.851	60.5365
T1328	DL-trihexyphenidyl hydrochloride	58947-95-8	-43.4946	-42.5112	-0.9834	13.3922
T1051	Tretinoin	302-79-4	-43.4705	-36.9505	-6.52	9.2788
T1458	Epalrestat	82159-09-9	-43.4705	-40.5617	-2.9088	8.6849
T4271	Diroximel Fumarete	1577222-14-0	-43.4521	-41.9029	-1.5492	5.9481
T1451	Levofloxacin Hemihydrate	138199-71-0	-43.428	-38.3161	-5.1119	1.8611
T1636	Paroxetine hydrochloride	78246-49-8	-43.4137	-41.3732	-2.0405	9.2595
T2222	Carbinoxamine Maleate Salt	3505-38-2	-43.4096	-41.1395	-2.2701	6.1158
T1628	Rivastigmine tartrate	129101-54-8	-43.3959	-42.0982	-1.2977	13.3176
T1275	Phentolamine mesilate	65-28-1	-43.3708	-41.4521	-1.9187	4.2772
T0375L	Atropine sulfate monohydrate	5908-99-6	-43.365	-41.1982	-2.1668	14.1958
T0700	Ursodeoxycholic acid	128-13-2	-43.3437	-36.4704	-6.8732	17.6996
T2593	Eflornithine hydrochloride Monohydrate	96020-91-6	-43.3399	-15.7635	-27.5764	6.9346
T0433	Artesunate	88495-63-0	-43.3329	-39.4086	-3.9243	2.3925
T2339	Apalutamide	956104-40-8	-43.329	-43.4075	0.0785	11.108
T3384	Wedelolactone	524-12-9	-43.3205	-42.8006	-0.52	3.5375
T0182L2	(??) Clopidogrel hydrogen sulfate	135046-48-9	-43.3183	-41.201	-2.1173	22.7281
T0750	Sulfisoxazole	127-69-5	-43.3164	-33.1637	-10.1527	4.6833
T1148	Menbutone	3562-99-0	-43.2973	-37.6546	-5.6428	5.2734
T7126	Hydroquinine	522-66-7	-43.2938	-41.9843	-1.3096	18.638
T0693	Niflumic acid	4394-00-7	-43.2885	-40.0832	-3.2053	2.9796
T1185	Rofecoxib	162011-90-7	-43.2813	-43.3704	0.0891	7.2071
T0260	Meclofenamate Sodium	6385-02-0	-43.2303	-40.9278	-2.3025	5.1297
T3857	magnolin	31008-18-1	-43.2279	-41.5232	-1.7048	11.7107
T0839L	(S)-(+)-Ketoprofen	22161-81-5	-43.2255	-36.6881	-6.5374	6.9035
T1491	Atovaquone	95233-18-4	-43.2251	-38.5074	-4.7177	25.4339
T4499	Mequitazine	29216-28-2	-43.2238	-42.7558	-0.468	75.2647
T1319	Diatrizoate sodium	737-31-5	-43.1714	-39.7502	-3.4212	8.4117
T0788	Spectinomycin dihydrochloride	21736-83-4	-43.1677	-36.0717	-7.096	4.8362
T4565	Tafamidis	594839-88-0	-43.1616	-38.5173	-4.6443	1.3514
T2845	Imperatorin	482-44-0	-43.1613	-42.7834	-0.3779	5.0962
T0921	Sulfabenzamide	127-71-9	-43.1542	-33.1423	-10.0119	2.898
T0885	Sulfamethoxazole	723-46-6	-43.1541	-33.1224	-10.0317	3.5362
T7176	Pitolisant hydrochloride	903576-44-3	-43.1501	-42.1269	-1.0232	4.6116
T4568	Dolasetron	115956-12-2	-43.1498	-41.5532	-1.5966	8.5669
T6574	Lumiracoxib	220991-20-8	-43.1425	-39.7448	-3.3977	3.9558
T0988	Trimetazidine dihydrochloride	13171-25-0	-43.1392	-26.0399	-17.0993	4.8727
T4017	Relebactam	1174018-99-5	-43.1052	-36.3658	-6.7394	9.2787

T0085	Entecavir hydrate	209216-23-9	-43.1023	-42.5867	-0.5156	4.8213
T0718	Brivudine	69304-47-8	-43.0935	-42.5084	-0.5852	3.6764
T2589	Dalasetron Mesylate Hydrate	878143-33-0	-43.0903	-41.8843	-1.206	9.6696
TJS2190	Nordihydroguaiaretic acid	500-38-9	-43.077	-41.9174	-1.1596	7.661
T0766	Promazine hydrochloride	53-60-1	-43.0736	-43.6875	0.614	7.1374
T1308	Orphenadrine hydrochloride	341-69-5	-43.0106	-40.7875	-2.2231	7.7861
T4979	Procyclidine hydrochloride	1508-76-5	-43.0073	-40.6537	-2.3537	16.6178
T2046	Nimorazole	6506-37-2	-42.9887	-39.197	-3.7918	9.5823
T2202	Lithocholic acid	434-13-9	-42.9852	-38.0346	-4.9506	20.9198
T4733	Sucrose	57-50-1	-42.9822	-39.6686	-3.3136	12.8511
T0980	Nifenazone	2139-47-1	-42.9739	-42.1237	-0.8502	3.7204
T6443	Chloroprocaine HCl	3858-89-7	-42.9712	-40.3974	-2.5738	4.8288
T1509	Cyclobenzaprine hydrochloride	6202-23-9	-42.9682	-42.3266	-0.6416	5.0879
T5S1569	AKBA	67416-61-9	-42.9603	-33.8287	-9.1316	9.4335
T0947	Dexamethason acetate	1177-87-3	-42.9551	-38.839	-4.1162	14.4499
T7421	Melperone	3575-80-2	-42.9475	-41.6846	-1.2629	7.8185
T8139	Sulfamethoxazole sodium	4563-84-2	-42.9455	-32.8216	-10.1238	3.5728
T0688	Ganciclovir	82410-32-0	-42.935	-41.7926	-1.1424	4.6538
T0070	Pentoxifylline	6493-05-6	-42.9339	-41.8898	-1.0441	8.0488
T0009	Flubendazole	31430-15-6	-42.9224	-43.1536	0.2312	1.9873
T0955	Oxyclozanide	2277-92-1	-42.9219	-41.1044	-1.8174	2.831
T0748	Sulfameter	651-06-9	-42.9194	-42.8486	-0.0708	7.4099
T1168	Desonide	638-94-8	-42.9118	-41.6286	-1.2833	26.8548
T1499	Dutasteride	164656-23-9	-42.8929	-42.4634	-0.4296	9.3484
T2974	Cyclovirobuxin D	860-79-7	-42.8919	-31.1707	-11.7212	10.7934
T2O2798	D-Trehalose	99-20-7	-42.8841	-40.415	-2.4692	7.4392
T1155	Mebendazole	31431-39-7	-42.8822	-43.0752	0.193	7.9696
T1463	Ramelteon	196597-26-9	-42.87	-42.1981	-0.6719	5.2314
T2856	Epicatechin	490-46-0	-42.8694	-37.6465	-5.2229	2.8305
T6547	Ipratropium Bromide	22254-24-6	-42.8514	-40.4869	-2.3645	14.4223
T3383	Cimifugin	37921-38-3	-42.8389	-39.2548	-3.5841	8.348
T1089	Etomidate	33125-97-2	-42.838	-42.7611	-0.0769	9.4539
T0375	Atropine	51-55-8	-42.8276	-28.5842	-14.2434	6.7688
T3165	Metoprolol Succinate	98418-47-4	-42.8152	-40.0756	-2.7395	4.2987
T0122	Bengenin	477-90-7	-42.8101	-39.9693	-2.8407	4.6481
T1643	Penciclovir	39809-25-1	-42.8067	-41.4492	-1.3575	5.1435
T1555	Diclofenac sodium	15307-79-6	-42.7907	-38.7319	-4.0588	6.7392
T1281	Tropicamide	1508-75-4	-42.7814	-41.8963	-0.8851	13.5726
T3122	Fangchinoline	436-77-1	-42.7785	-41.6529	-1.1256	9.1224
T6694	Tamibarotene	94497-51-5	-42.7758	-41.8731	-0.9027	1.3492
T1189	Milnacipran hydrochloride	101152-94-7	-42.7627	-41.1081	-1.6546	14.7312
T0026	Sulfalen	152-47-6	-42.7531	-40.675	-2.0781	7.1628
T2725	Scopolamine HBr	114-49-8	-42.7336	-40.2484	-2.4852	8.7524
T1543	Naftifine hydrochloride	65473-14-5	-42.732	-40.5649	-2.1671	8.3272
T0196	Diclofenac	15307-86-5	-42.719	-38.5545	-4.1645	6.1681
T7477	Argireline	91-75-8	-42.7153	-41.5176	-1.1977	11.412
T7533	Taurolidine	19388-87-5	-42.6934	-23.2017	-19.4917	1.4265
T6212	Combretastatin A4	117048-59-6	-42.6892	-41.5336	-1.1556	7.5102

T0173	Nylidrin hydrochloride	849-55-8	-42.6821	-40.3634	-2.3187	9.7928
T4333	Quinine dihydrochloride	60-93-5	-42.6313	-41.2961	-1.3352	6.28
T1585	Metolazone	17560-51-9	-42.6294	-41.4826	-1.1468	9.0681
T2333	Efinaconazole	164650-44-6	-42.6231	-34.8457	-7.7774	36.327
T0298	Dimenhydrinate	523-87-5	-42.6226	-42.2521	-0.3705	9.4061
T1113	Imazalil	35554-44-0	-42.6171	-42.6241	0.007	4.404
T1158	Phenoxybenzamine hydrochloride	63-92-3	-42.6147	-42.3311	-0.2835	15.7771
T1123	(S)-(+)-Camptothecin	7689-03-4	-42.5972	-41.8194	-0.7777	3.6956
T0408	Dehydrocholic acid	81-23-2	-42.5968	-36.8985	-5.6983	13.8045
T4276	Hydroxyfasudil Hydrochloride	155558-32-0	-42.5957	-38.9168	-3.679	15.3384
T0085L	Entecavir	142217-69-4	-42.5952	-41.754	-0.8412	6.6318
T2550	Tolmetin	26171-23-3	-42.5912	-41.7195	-0.8717	4.6127
T0711	Niclosamide	50-65-7	-42.5905	-42.2422	-0.3482	3.3496
T6470	Diclofenac Potassium	15307-81-0	-42.5831	-38.7893	-3.7938	6.2296
T0473	Arbutin	497-76-7	-42.5779	-40.3338	-2.2441	3.6435
T1789	Obeticholic Acid	459789-99-2	-42.5751	-44.1501	1.5751	25.6274
T6644	Rolipram	61413-54-5	-42.5524	-41.9527	-0.5997	8.5494
T0850	Primaquine diphosphate	63-45-6	-42.532	-40.8928	-1.6392	4.0096
T0167	Vinpocetine	42971-09-5	-42.5309	-43.8147	1.2839	9.2167
T6702	Terbinafine	91161-71-6	-42.5249	-40.5163	-2.0086	4.9568
T0027	Octisalate	118-60-5	-42.5183	-40.9591	-1.5591	7.7778
T0432	Diclofenac diethylamine	78213-16-8	-42.5176	-38.2584	-4.2592	6.2253
T7582	tafamidis meglumine	951395-08-7	-42.5027	-33.6175	-8.8851	2.0233
T6359	1-Hexadecanol	36653-82-4	-42.4964	-42.1368	-0.3596	7.491
T0858	Flufenamic acid	530-78-9	-42.4911	-39.474	-3.0172	3.6476
T8489	Minodronic acid monohydrate	155648-60-5	-42.4867	-36.8704	-5.6164	9.7642
T4412	Roquinimex	84088-42-6	-42.4849	-36.7075	-5.7774	6.539
T1217	Butenafine hydrochloride	101827-46-7	-42.4842	-42.1247	-0.3596	8.685
T1411	Terbinafine hydrochloride	78628-80-5	-42.4838	-40.5711	-1.9126	5.6858
T3371	Quillaic Acid	631-01-6	-42.4634	-37.8955	-4.568	17.3057
T1233	Oxolinic acid	14698-29-4	-42.456	-33.8284	-8.6277	4.5048
T0962	L-Hyoscyamine	101-31-5	-42.4459	-41.5563	-0.8896	7.9084
T1738	Taxifolin	480-18-2	-42.433	-38.3074	-4.1257	7.1286
T1445	Agomelatine	138112-76-2	-42.4278	-41.3248	-1.103	6.3301
T0274	Cinoxacin	28657-80-9	-42.4128	-34.3044	-8.1084	3.1748
T8049	Pentadecanoic acid	1002-84-2	-42.4098	-39.2815	-3.1284	13.8793
T3060	Fasudil hydrochloride	105628-07-7	-42.4015	-40.1743	-2.2273	16.5844
T1553	Chlorpheniramine maleate	113-92-8	-42.3995	-40.3419	-2.0576	6.8414
T0092	Antazoline hydrochloride	2508-72-7	-42.3927	-41.5925	-0.8002	9.7503
T1317	Cinchonidine	485-71-2	-42.368	-42.0022	-0.3659	8.5585
T5005	Eperisone hydrochloride	56839-43-1	-42.3632	-41.8587	-0.5046	9.448
T0084	Moclobemide	71320-77-9	-42.357	-41.4416	-0.9153	2.9527
T0503	Meglumine	6284-40-8	-42.3375	-37.3575	-4.9801	3.3346
T4274	Melitracen hydrochloride	10563-70-9	-42.3357	-39.5154	-2.8203	7.0056
T6430	Calcifediol	19356-17-3	-42.3352	-42.1717	-0.1635	19.0485
T0445	Promethazine	60-87-7	-42.3314	-39.9293	-2.4021	13.4803
T1134	Clofoctol	37693-01-9	-42.3057	-41.7576	-0.5482	24.2091
T0054	Disulfiram	97-77-8	-42.3029	-42.7387	0.4359	8.0662

T0120	Voriconazole	137234-62-9	-42.2957	-41.5362	-0.7594	23.3964
T0337	Prednisolone-21-acetate	52-21-1	-42.292	-37.841	-4.451	17.3381
T2966	Beta-Sitosterol	83-46-5	-42.2884	-41.9874	-0.301	15.4219
T1145	Diminazene Aceturate	908-54-3	-42.2557	-25.8027	-16.4529	11.6232
T0445L	Promethazine hydrochloride	58-33-3	-42.2387	-40.3338	-1.9049	13.7263
T0487	Metoprolol tartrate	56392-17-7	-42.2231	-40.8536	-1.3695	4.2451
T1502	Vildagliptin	274901-16-5	-42.2118	-30.2248	-11.9869	5.589
T0675	Topiramate	97240-79-4	-42.1982	-40.9368	-1.2615	2.5552
T0690	Quinine	130-95-0	-42.186	-40.6674	-1.5186	6.1179
T0204	Protriptyline hydrochloride	1225-55-4	-42.1815	-40.7136	-1.4679	3.9688
T0074	Chlorprothixene	113-59-7	-42.156	-40.9662	-1.1899	4.2395
T2523	Rufinamide	106308-44-5	-42.149	-41.1218	-1.0272	3.3865
T0894	Thiamine hydrochloride	67-03-8	-42.1208	-40.6239	-1.497	7.0183
T1002	Etodolac	41340-25-4	-42.0876	-35.4312	-6.6563	6.7621
T0714	Oxfendazole	53716-50-0	-42.0455	-42.383	0.3375	1.0042
T2920	Berbamine dihydrochloride	6078-17-7	-42.0444	-38.892	-3.1524	14.2901
T4542	Clonixin	17737-65-4	-42.0011	-39.012	-2.9891	4.8096
T8349	Cyclofenil	2624-43-3	-41.9964	-41.6177	-0.3787	8.4092
T1336	Benztropine mesylate	132-17-2	-41.9961	-41.3516	-0.6445	6.1067
T1381	Milbemycin oxime	129496-10-2	-41.9916	-41.7909	-0.2008	18.1963
T0386L	Ropivacaine	84057-95-4	-41.9742	-40.4857	-1.4885	10.1975
T0757	Omeprazole	73590-58-6	-41.9733	-40.2998	-1.6735	6.7972
T0074L	Chlorprothixene hydrochloride	6469-93-8	-41.9695	-40.7575	-1.212	5.1621
T5593	Ala-Gln	39537-23-0	-41.958	-37.8578	-4.1001	5.6672
T1498	Indapamide	26807-65-8	-41.9574	-42.3852	0.4278	6.4847
T6737	Zaltoprofen	74711-43-6	-41.9442	-38.6691	-3.2751	4.5491
T0205	Brompheniramine hydrogen maleate	980-71-2	-41.9384	-40.1481	-1.7903	5.346
T1240	Flumazenil	78755-81-4	-41.9301	-42.3762	0.4461	1.8246
T6497	Fenoprofen Calcium	34597-40-5	-41.9199	-35.4374	-6.4825	5.495
T1307	Carmofur	61422-45-5	-41.9163	-42.0298	0.1134	12.2159
T3S0807	Berbamine	478-61-5	-41.9087	-41.7322	-0.1765	13.9624
T1327	Nortriptyline hydrochloride	894-71-3	-41.9031	-41.1267	-0.7764	3.5648
T8709	Nandrolone phenpropionate	62-90-8	-41.8989	-41.6309	-0.268	11.4223
T0145	Felodipine	72509-76-3	-41.8857	-41.737	-0.1487	13.8629
T1204	Bendroflumethiazide	73-48-3	-41.8675	-42.9951	1.1276	13.8555
T3614	Isoprinosine	36703-88-5	-41.8637	-41.2299	-0.6339	5.4296
T2848	Osthole	484-12-8	-41.8625	-40.4022	-1.4604	6.5698
T0184	Fenoprofen calcium dihydrate	53746-45-5	-41.8519	-35.0394	-6.8126	5.3709
T2393	Efavirenz	154598-52-4	-41.8514	-41.4152	-0.4361	4.1774
T0893	Tinidazole	19387-91-8	-41.8316	-40.0531	-1.7785	4.5945
T6631	Quinine HCl Dihydrate	6119-47-7	-41.8303	-40.3751	-1.4552	8.1489
T0229	Rivastigmine	123441-03-2	-41.8143	-41.3934	-0.4209	13.198
T0114	Trichlormethiazide	133-67-5	-41.8112	-41.4309	-0.3803	4.4995
T5954	Alpha-Methyldopa Sesquihydrate	41372-08-1	-41.807	-37.8105	-3.9964	9.1406
T8039	Brucine sulfate heptahydrate	60583-39-3	-41.8067	-40.7752	-1.0314	3.2904
T0370	Pheniramine maleate	132-20-7	-41.8044	-40.0868	-1.7176	5.1039
T0175L	Amiloride hydrochloride dihydrate	17440-83-4	-41.7964	-37.3865	-4.41	7.6055
T4056	Cyclizine hydrochloride	303-25-3	-41.7907	-24.2253	-17.5654	7.3301

T6615	Ondansetron	99614-02-5	-41.7829	-42.9523	1.1694	17.1954
T2182	Scopolamine HBr trihydrate	6533-68-2	-41.7686	-40.2885	-1.4801	7.7909
T1127	Beclomethasone dipropionate	5534-09-8	-41.7532	-39.3366	-2.4165	23.3421
T1538	Melphalan	148-82-3	-41.751	-37.3528	-4.3982	5.9972
T2594	Levomilnacipran	96847-55-1	-41.7375	-37.7221	-4.0154	22.9454
T1021	R-(+)-Lansoprazole	138530-94-6	-41.7276	-40.8018	-0.9259	9.2506
T8135	Minoxidil sulphate	83701-22-8	-41.7249	-37.9472	-3.7777	6.6125
T0330	Diphenhydramine hydrochloride	147-24-0	-41.7232	-41.1396	-0.5836	9.415
T0770	Carbadox	6804-07-5	-41.6969	-36.6601	-5.0368	5.6817
T1401	Meclofenoxate hydrochloride	3685-84-5	-41.6755	-37.8001	-3.8755	2.9128
T1409	Methocarbamol	532-03-6	-41.6713	-40.0298	-1.6415	3.3332
T0498	Sasapyrine	552-94-3	-41.6578	-37.0814	-4.5764	4.1108
T1416	Zidovudine	30516-87-1	-41.6505	-40.8019	-0.8486	4.1478
T1137	Clorsulon	60200-06-8	-41.6461	-39.988	-1.6581	10.1483
T1220	Ethidium bromide	1239-45-8	-41.6454	-40.459	-1.1864	10.5509
T1477	AICAR (Acadesine)	2627-69-2	-41.6449	-40.5358	-1.1091	5.7142
T2177	Kaempferol	520-18-3	-41.6433	-36.6971	-4.9462	3.7818
T2O2733	cystine	56-89-3	-41.637	-36.3162	-5.3208	1.3153
T6368	Abscisic Acid	21293-29-8	-41.6358	-36.1572	-5.4786	12.7638
T6367	Abacavir sulfate	188062-50-2	-41.6174	-41.3485	-0.2689	5.5315
T3495	Tasimelteon	609799-22-6	-41.6172	-42.0278	0.4107	6.0787
T0386	Ropivacaine hydrochloride	98717-15-8	-41.6151	-40.1875	-1.4275	8.2933
T1284	Megestrol acetate	595-33-5	-41.6106	-41.0496	-0.561	11.1788
T0716	Molindone hydrochloride	15622-65-8	-41.6075	-43.6702	2.0627	10.336
T1403	Climbazole	38083-17-9	-41.6038	-39.0264	-2.5773	9.1451
T0030	Bupivacaine hydrochloride	18010-40-7	-41.5972	-40.8143	-0.7829	10.3453
T0488	Finasteride	98319-26-7	-41.5774	-42.065	0.4875	7.8327
T1388	Fluconazole	86386-73-4	-41.5759	-40.0651	-1.5107	14.7984
T1536	Promestriene	39219-28-8	-41.5721	-40.9097	-0.6625	3.0244
T2999	Gastrodin	62499-27-8	-41.5622	-38.5497	-3.0125	7.3856
T2197	10-Deacetylbaccatin III	32981-86-5	-41.5416	-40.4368	-1.1048	48.4491
T0175	Amiloride hydrochloride	2016-88-8	-41.5394	-37.5822	-3.9573	4.8007
T2533	Alcaftadine	147084-10-4	-41.5273	-42.5037	0.9764	9.6377
T3279	Silver sulfadiazine	22199-08-2	-41.5234	-38.0379	-3.4855	2.5692
T2963	Cholic Acid	81-25-4	-41.5133	-33.2396	-8.2737	21.4716
T3002	Piperine	94-62-2	-41.5124	-40.6793	-0.8331	4.9351
T6620	Ospemifene	128607-22-7	-41.5113	-41.7312	0.2199	6.4947
T0286	Bephenium Hydroxynaphthoate	3818-50-6	-41.5105	-39.3081	-2.2023	5.267
T2868	Evodiamine	518-17-2	-41.5088	-41.8718	0.363	0
T2149	Eburnalritardo	4880-88-0	-41.5036	-40.6512	-0.8524	8.4889
T4382	Proguanil hydrochloride	637-32-1	-41.5031	-38.8968	-2.6063	2.5264
T1086	Vitamin D2	50-14-6	-41.4531	-41.4769	0.0237	20.2398
T8125	Quinidine sulfate	6591-63-5	-41.4492	-38.4951	-2.9541	9.0331
T6523	Guanosine	118-00-3	-41.4417	-40.3005	-1.1412	1.9443
T0989	7-Aminocephalosporanic acid	957-68-6	-41.4296	-30.903	-10.5266	5.559
T3000	Magnolol	528-43-8	-41.4264	-41.1498	-0.2766	5.3225
T0741	Sulfamethazine	57-68-1	-41.4197	-41.5702	0.1505	3.555
T0135	Estradiol valerate	979-32-8	-41.4143	-41.1987	-0.2156	3.8727

T3288	Sulfisomidine	515-64-0	-41.3755	-40.0735	-1.3019	5.7074
T6S0525	Farrerol	24211-30-1	-41.3714	-39.4102	-1.9612	11.5778
T6645	Ropivacaine HCl monohydrate	132112-35-7	-41.3676	-40.1367	-1.2309	9.1447
T0963	Naltrexone hydrochloride	16676-29-2	-41.3662	-32.1993	-9.1669	4.5073
T1229	Erdosteine	84611-23-4	-41.3569	-34.6604	-6.6964	2.6414
T1395	Diatrizoic Acid	117-96-4	-41.3431	-38.125	-3.218	9.0359
T6625	Ozagrel hydrochloride	78712-43-3	-41.3428	-33.1185	-8.2243	3.7553
T1187	Bifonazole	60628-96-8	-41.3406	-41.963	0.6224	15.4493
T0040	Quinestrol	152-43-2	-41.3233	-40.9431	-0.3801	10.3089
T0455	Clozapine	5786-21-0	-41.3176	-30.3017	-11.0159	4.932
T5041	Faropenem sodium	122547-49-3	-41.3146	-31.9093	-9.4053	5.4933
T0737	L-Ornithine	70-26-8	-41.3066	-15.381	-25.9256	2.4605
T0502	Zinc pyrithione	13463-41-7	-41.3023	-37.8412	-3.4611	0.3765
T0059	Diphenylpyraline hydrochloride	132-18-3	-41.2919	-40.97	-0.3219	5.6712
T2199	Myclobutanil	88671-89-0	-41.2912	-40.9337	-0.3575	14.7616
T0437	Inosine	58-63-9	-41.2875	-40.0292	-1.2584	4.3434
T4374	Edoxudine	15176-29-1	-41.2815	-39.976	-1.3055	6.5464
T4981	Anisotropine Methylbromide	80-50-2	-41.2729	-40.132	-1.1409	12.8908
T0939	Phenytoin	57-41-0	-41.2602	-41.5988	0.3386	14.1939
T0159	Pranoprofen	52549-17-4	-41.2595	-32.9069	-8.3525	2.938
T6529	Halobetasol Propionate	66852-54-8	-41.2459	-40.8535	-0.3923	18.4281
T6410	Bexarotene	153559-49-0	-41.2415	-35.5138	-5.7277	3.2146
T0249	Acecainide	32795-44-1	-41.2405	-39.5533	-1.6872	5.604
T6165	Bardoxolone Methyl	218600-53-4	-41.2216	-41.3087	0.0872	9.6391
T1709	2'-Deoxyinosine	890-38-0	-41.2196	-41.811	0.5914	7.2947
T6533	Hexestrol	84-16-2	-41.2144	-40.1591	-1.0554	12.1902
T1449	Hydrochlorothiazide	58-93-5	-41.1937	-40.0506	-1.1431	3.804
T7333	Pyrvinium pamoate	3546-41-6	-41.191	-41.9165	0.7254	10.7002
T6375	Alibendol	26750-81-2	-41.1829	-39.7803	-1.4026	4.1047
T0960	Iopanoic acid	96-83-3	-41.176	-38.1058	-3.0701	16.9335
T1153	Trimethoprim	738-70-5	-41.1733	-40.2337	-0.9396	37.2116
T2827	Asiatic acid	464-92-6	-41.1602	-38.6624	-2.4978	16.7079
T4559	Isocarboxazid	59-63-2	-41.1472	-39.4791	-1.6681	1.5077
T1269	Tripelennamine hydrochloride	154-69-8	-41.141	-38.5921	-2.5489	5.907
T1427	Fosfosal	6064-83-1	-41.1203	-24.5579	-16.5624	9.7408
T2525	Alosetron hydrochloride	122852-69-1	-41.1	-40.9066	-0.1934	2.8818
T3115	Nicarbazin	330-95-0	-41.0995	-40.2066	-0.8929	8.7849
T0920	Ambroxol	18683-91-5	-41.0888	-37.7919	-3.297	3.8994
T1019	Hexachlorophene	70-30-4	-41.073	-40.1499	-0.9231	12.5118
T6236	Ozagrel	82571-53-7	-41.072	-36.6731	-4.3989	3.1195
T7004	Tubercidin	69-33-0	-41.0669	-38.8997	-2.1672	5.1739
T2578	Didanosine	69655-05-6	-41.0443	-41.8096	0.7653	5.6121
T2184	Sulfachloropyridazine	80-32-0	-41.0378	-40.2569	-0.7809	5.9652
T0725	Isoliquiritigenin	961-29-5	-41.032	-39.2441	-1.7879	4.414
T5S2178	Fargesin	31008-19-2	-40.9975	-39.2951	-1.7024	16.3874
T6S0232	Eriodictyol	552-58-9	-40.9736	-36.8939	-4.0797	8.3767
T1267	Abacavir	136470-78-5	-40.9396	-40.5075	-0.432	7.8136
T2134	Dorzolamide hydrochloride	130693-82-2	-40.9362	-38.3097	-2.6265	1.1817

T1596	Chlormadinone acetate	302-22-7	-40.9295	-40.6685	-0.261	9.9938
T1590	Letrozole	112809-51-5	-40.9216	-39.8912	-1.0303	6.9687
T6373	Alendronate sodium hydrate	121268-17-5	-40.9213	-22.3734	-18.5478	6.0124
T6234	Ambroxol hydrochloride	23828-92-4	-40.921	-37.4698	-3.4512	4.2216
T1117	Clorgyline hydrochloride	17780-75-5	-40.9006	-40.3055	-0.5951	2.4741
T0463	Loxoprofen	68767-14-6	-40.8995	-35.2637	-5.6359	6.814
T12408	Perhexiline maleate	6724-53-4	-40.8965	-28.6119	-12.2846	17.2758
T4006	Pentostatin	53910-25-1	-40.8625	-37.8335	-3.0289	3.9152
T3278	Piribedil	3605-01-4	-40.8351	-41.4252	0.5901	7.0059
T1639	Amlexanox	68302-57-8	-40.8217	-36.9963	-3.8254	13.8501
T0113	Mebhydrolin napadisylate	6153-33-9	-40.818	-39.2095	-1.6085	3.3008
T0674	Lansoprazole	103577-45-3	-40.7823	-38.0912	-2.6911	6.4378
T6647	Rotigotine	99755-59-6	-40.774	-42.6754	1.9014	7.9527
T0375L2	Atropine sulfate	55-48-1	-40.7385	-39.7364	-1.0022	9.5572
T5720	Boldine	476-70-0	-40.7356	-38.3265	-2.4091	5.7745
T6723	Voglibose	83480-29-9	-40.7142	-30.166	-10.5482	4.7824
T3001	Honokiol	35354-74-6	-40.6822	-39.1051	-1.5771	6.8805
T0760	Cholesterol	57-88-5	-40.6776	-39.6283	-1.0493	12.4873
T0719	Tetrabenazine	58-46-8	-40.6556	-27.9382	-12.7173	6.4441
T2411	Luliconazole	187164-19-8	-40.6427	-40.0878	-0.555	10.3359
T0394	Moxisylyte hydrochloride	964-52-3	-40.6393	-38.0744	-2.5649	17.5393
T0822	Cianidanol	154-23-4	-40.6389	-37.5173	-3.1216	1.5559
T0075	Nicorandil	65141-46-0	-40.6237	-37.4494	-3.1742	6.568
T1787	Levobupivacaine	27262-47-1	-40.6224	-41.3203	0.6979	9.989
T7085	Mebhydroline	524-81-2	-40.6186	-38.9931	-1.6254	3.6026
T7117	Sapropterin Hydrochloride	69056-38-8	-40.587	-39.6609	-0.9261	3.595
T4562	Ramosetron	132036-88-5	-40.585	-39.7928	-0.7922	3.3815
T3290	Pikamilone	34562-97-5	-40.5673	-34.0018	-6.5654	3.01
T3S0738	yangonin	500-62-9	-40.5652	-39.7555	-0.8097	5.5218
T1042	Granisetron hydrochloride	107007-99-8	-40.5585	-39.8531	-0.7054	1.9632
T1659	Melatonine	73-31-4	-40.5465	-39.445	-1.1015	2.9068
T1258	Nabumetone	42924-53-8	-40.5321	-38.8753	-1.6568	3.0136
T1313	Nitrofurantoin	67-20-9	-40.5279	-38.7245	-1.8034	5.7236
T0913	Sulfamerazine	127-79-7	-40.5162	-39.7723	-0.7439	5.4026
T6221	Nalmefene hydrochloride	58895-64-0	-40.5013	-30.9209	-9.5803	6.6437
T0036	Enoxolone	471-53-4	-40.5006	-38.0496	-2.4511	4.7741
T0482	Sertraline hydrochloride	79559-97-0	-40.4854	-37.9707	-2.5147	5.8666
T1264	Ciprofibrate	52214-84-3	-40.4716	-36.0671	-4.4045	11.2034
T0890	Mefenamic acid	61-68-7	-40.4692	-37.6501	-2.8191	5.0309
T1343L	Crystal Violet	548-62-9	-40.466	-37.872	-2.5941	14.3156
T3026	(-)-Huperzine A	102518-79-6	-40.4254	-36.4862	-3.9392	3.6559
T5292	N-Acetyl-L-tyrosine	537-55-3	-40.4204	-40.0408	-0.3796	7.9434
T0802	Procaine hydrochloride	51-05-8	-40.4101	-37.5344	-2.8757	6.8217
T2175	Apigenin	520-36-5	-40.3735	-35.8693	-4.5042	3.0616
T2279	Tizoxanide	173903-47-4	-40.3727	-39.7834	-0.5893	4.0034
T0892	Diprophylline	479-18-5	-40.3633	-38.5144	-1.8489	5.572
T1582L	Naproxen sodium	26159-34-2	-40.3617	-30.6511	-9.7106	2.9471
T1647	Ronidazole	7681-76-7	-40.3499	-39.444	-0.9059	5.4073

T1198	Tetracaine hydrochloride	136-47-0	-40.3249	-37.8688	-2.4561	3.2752
T6603	Nelarabine	121032-29-9	-40.3243	-38.5684	-1.756	10.5103
T0684	Ribavirin	36791-04-5	-40.3227	-38.2505	-2.0723	2.8905
T6215	Abiraterone Acetate	154229-18-2	-40.3017	-39.7357	-0.566	8.308
T6566	Levobupivacaine HCl	27262-48-2	-40.2909	-39.5818	-0.7092	10.6157
T8145	Benzyl cinnamate	103-41-3	-40.2871	-39.0338	-1.2533	7.5165
T4572	Desoximetasone	382-67-2	-40.2536	-39.4679	-0.7857	10.7045
T6153	Icotinib	610798-31-7	-40.2434	-39.2038	-1.0396	1.1131
T0784	Tolfenamic Acid	13710-19-5	-40.2081	-37.4729	-2.7352	4.1739
T7812	Acitazanolast	114607-46-4	-40.2076	-32.5818	-7.6258	3.2832
T0930	Sulfadiazine	68-35-9	-40.173	-39.541	-0.632	9.4846
T1146	Nifedipine	21829-25-4	-40.1391	-37.4563	-2.6828	13.0843
T0270	Hydrocortisone butyrate	13609-67-1	-40.1368	-38.442	-1.6948	21.2925
T4201	Avibactam sodium	1192491-61-4	-40.1079	-37.0717	-3.0362	4.9315
T0678	Amitriptyline hydrochloride	549-18-8	-40.0937	-40.2588	0.165	9.6097
T6543	Indoximod	110117-83-4	-40.0493	-39.7538	-0.2955	10.0209
T0008	Phenytoin sodium	630-93-3	-40.032	-38.8642	-1.1678	17.1201
T1139	Salbutamol	18559-94-9	-40.0233	-37.8942	-2.1291	6.2938
T6336	Amonafide	69408-81-7	-40.0174	-39.9338	-0.0837	7.5771
T7636	Phenprocoumon	435-97-2	-39.9981	-36.8434	-3.1547	9.2014
T0131	Cepharanthine	481-49-2	-39.9867	-35.4329	-4.5537	8.3338
T1325	Carprofen	53716-49-7	-39.9849	-32.9081	-7.0768	2.7206
T0504	Cyclandelate	456-59-7	-39.9796	-38.8164	-1.1633	9.0304
T0012	Cinchonine	118-10-5	-39.9624	-38.1924	-1.77	10.3801
T2858	Baicalein	491-67-8	-39.93	-37.6731	-2.2569	3.2276
T7164	Levalbuterol tartrate	661464-94-4	-39.9254	-36.4917	-3.4337	7.6754
T0034	Cortisone acetate	50-04-4	-39.9009	-39.4619	-0.439	8.9414
T0029	Procaine	59-46-1	-39.8899	-38.0562	-1.8336	7.9437
T0994	Ketotifen fumarate	34580-14-8	-39.87	-38.5696	-1.3004	5.7782
T2752	2'-Deoxyadenosine monohydrate	16373-93-6	-39.8439	-40.672	0.8281	6.6847
T4983	Isoetharine mesylate salt	7279-75-6	-39.8269	-38.219	-1.6079	6.0711
T7940	Milnacipran ((1S-cis) hydrochloride)	175131-60-9	-39.7907	-23.8544	-15.9363	14.5939
T1591L	Cytidine	65-46-3	-39.784	-38.6512	-1.1328	10.2068
T1528	Azasetron hydrochloride	123040-16-4	-39.7809	-37.5491	-2.2318	5.196
T0873	Dinitolmide	148-01-6	-39.7795	-37.9418	-1.8377	4.6832
T7537	Norgestimate	35189-28-7	-39.7701	-39.2822	-0.4879	22.5288
T0754	Sulfamethizole	144-82-1	-39.7615	-38.6278	-1.1337	7.4975
T3003	5-hydroxytryptophan	56-69-9	-39.7589	-34.7346	-5.0243	2.7646
T0698	Dioxybenzone	131-53-3	-39.7545	-37.1126	-2.6419	4.5313
T0872L	Amprolium Hydrochloride	137-88-2	-39.736	-37.5942	-2.1417	8.2304
T1038	Fludarabine	21679-14-1	-39.73	-38.396	-1.334	5.6488
T0470	Nefopam hydrochloride	23327-57-3	-39.7286	-37.4789	-2.2497	2.4382
T0511	Tolperisone hydrochloride	3644-61-9	-39.7157	-39.3726	-0.3431	5.845
T0830	Bithionol	97-18-7	-39.7143	-39.4474	-0.2669	2.0868
T1056	Isoprenaline hydrochloride	51-30-9	-39.6782	-36.8132	-2.865	2.3457
T1180	Blonanserin	132810-10-7	-39.6772	-38.9572	-0.72	6.5389
T1379	L-5-Hydroxytryptophan	4350-09-8	-39.6681	-34.9086	-4.7595	3.3637
T5S0802	Palmatine	3486-67-7	-39.6651	-41.3006	1.6355	3.7058

T0144	Ftaxilide	19368-18-4	-39.6441	-37.9099	-1.7342	6.8078
T1094	Tolnaftate	2398-96-1	-39.6173	-39.6681	0.0508	10.5914
T0472	Venlafaxine hydrochloride	99300-78-4	-39.615	-38.5091	-1.1058	7.7717
T0510	Sulfaguanidine	57-67-0	-39.581	-36.6522	-2.9287	3.8293
T1632	Amitraz	33089-61-1	-39.5785	-39.0203	-0.5581	4.3925
T0715	Chlormezanone	80-77-3	-39.5558	-40.26	0.7042	4.442
T0480	Doxofylline	69975-86-6	-39.554	-39.1119	-0.442	3.0358
T2547	Droxidopa	23651-95-8	-39.5459	-31.8074	-7.7385	5.7801
T2921	Sinomenine hydrochloride	6080-33-7	-39.537	-29.2238	-10.3132	71.5801
T6992	Sodium sulfadiazine	547-32-0	-39.5347	-29.9189	-9.6157	3.8493
T0367	Fluocinolone acetonide	67-73-2	-39.5181	-37.9204	-1.5978	14.2202
T2226	Pergolide mesylate salt	66104-23-2	-39.5098	-39.5665	0.0567	4.0527
T1946	Felbamate	25451-15-4	-39.5085	-38.4922	-1.0163	6.1066
T2395L	Vortioxetine HBr	960203-27-4	-39.5055	-37.6002	-1.9053	4.6439
T0670	L-arginine monohydrochloride	1119-34-2	-39.4591	-29.9632	-9.4959	2.5998
T1060	Flumequine	42835-25-6	-39.4563	-31.0814	-8.375	0.6418
T0306	Undecenoic acid	112-38-9	-39.4275	-35.6689	-3.7585	6.1554
T0102	Naloxone hydrochloride	357-08-4	-39.4132	-30.008	-9.4052	5.615
T2965	Deoxycholic acid	83-44-3	-39.4061	-36.5439	-2.8622	19.4812
T2S2172	nerolidol	7212-44-4	-39.404	-39.2284	-0.1756	9.2794
T0853	Adenosine	58-61-7	-39.3959	-38.0346	-1.3614	4.4894
T1610	Metaxalone	1665-48-1	-39.393	-39.6347	0.2417	1.4122
T2558	Cladribine	4291-63-8	-39.3696	-38.3772	-0.9924	3.9699
T1582	Naproxen	22204-53-1	-39.3568	-30.9119	-8.445	5.5329
T0768	Oxolamine citrate	1949-20-8	-39.3343	-37.1121	-2.2222	11.902
T0888	4-Biphenylacetic acid	5728-52-9	-39.3264	-35.3814	-3.945	2.7977
T0747	Sulfathiazole	72-14-0	-39.3192	-38.9864	-0.3327	5.9902
T1454	Aciclovir	59277-89-3	-39.3028	-37.5334	-1.7695	1.8657
T0677	Chlorothiazide	58-94-6	-39.2951	-38.6211	-0.674	4.5322
T3732	Sebacic acid	111-20-6	-39.2853	-35.2063	-4.079	6.2771
T6446	Clevudine	163252-36-6	-39.282	-37.7141	-1.5678	3.9818
T6873	Lauric Acid	143-07-7	-39.2542	-34.7608	-4.4934	8.2035
T0297	Clofarabine	123318-82-1	-39.2422	-37.975	-1.2672	6.9438
T1043	Salifungin	3679-64-9	-39.2391	-38.0495	-1.1895	2.0817
T2520	Desloratadine	100643-71-8	-39.2387	-38.1757	-1.063	7.8626
T0855	(R)-Naproxen	23979-41-1	-39.2194	-35.3976	-3.8218	9.127
T2366	Tipiracil hydrochloride	183204-72-0	-39.2168	-37.9235	-1.2933	2.4014
T0724	Formononetin	485-72-3	-39.2116	-39.2109	-0.0007	5.9938
T7583	solriamfetol	178429-62-4	-39.1914	-35.2423	-3.9491	7.1595
T1568	Amoxapine	14028-44-5	-39.1842	-38.5354	-0.6488	1.5272
T2898	Andrographolide	5508-58-7	-39.1766	-37.4719	-1.7047	12.4878
T7116	Sodium gualenate	6223-35-4	-39.1689	-31.5462	-7.6227	10.9981
T2548	Diflorasone	2557-49-5	-39.1616	-35.9741	-3.1875	17.3409
T1182	Propionyl-L-carnitine hydrochloride	119793-66-7	-39.1614	-34.3478	-4.8136	8.4801
T0820	Triamcinolone acetonide	76-25-5	-39.1301	-37.3636	-1.7665	12.6295
T1144	Lidocaine hydrochloride	73-78-9	-39.1247	-38.2759	-0.8488	5.7326
T5325	Nifurtimox	23256-30-6	-39.1217	-36.9341	-2.1876	3.1592
T8395	Nomegestrol acetate	58652-20-3	-39.1074	-38.136	-0.9714	15.1294

T5940	Proxyphylline	603-00-9	-39.09	-38.3616	-0.7284	8.3549
T8396	Prednisone acetate	125-10-0	-39.0697	-37.1756	-1.894	11.1315
T0897	Furacilin	59-87-0	-39.062	-37.781	-1.281	0.5548
T2204	Diaveridine	5355-16-8	-39.0567	-38.4561	-0.6006	30.1161
T0476	Abbolactone	52-01-7	-39.0185	-39.6397	0.6213	6.1872
T1286	Vincamine	1617-90-9	-39.0176	-35.7659	-3.2517	13.3074
T0291	Flurbiprofen	5104-49-4	-39.0143	-35.5228	-3.4915	9.3745
T6372	Albendazole Oxide	54029-12-8	-39.0114	-38.6343	-0.3771	2.4412
T3S0364	L-Arginine	74-79-3	-38.9975	-31.4002	-7.5973	2.9488
T0796	Propitocaine hydrochloride	1786-81-8	-38.9935	-38.7181	-0.2755	3.2526
T4429	Rauwolscine hydrochloride	6211-32-1	-38.9623	-38.2763	-0.686	6.5822
T0169	Nefiracetam	77191-36-7	-38.9604	-38.7249	-0.2356	7.4315
T2968	Hyodeoxycholic acid	83-49-8	-38.9596	-39.9443	0.9847	13.3787
T0083	Eplerenone	107724-20-9	-38.9535	-38.6665	-0.287	7.9692
T0272	Nilutamide	63612-50-0	-38.9456	-39.0265	0.081	7.3429
T0035	Dienestrol	84-17-3	-38.9391	-37.2888	-1.6502	6.487
T2804	Panaxtriol	32791-84-7	-38.9388	-39.5297	0.5909	15.4103
T2210	Genipin	6902-77-8	-38.9369	-37.7879	-1.1489	4.9237
T0923	Busulfan	55-98-1	-38.9254	-37.5775	-1.3479	4.5402
T0516	Sodium dehydrocholate	145-41-5	-38.9142	-35.6816	-3.2325	9.7375
T2530	Levosimendan	141505-33-1	-38.8912	-38.7948	-0.0964	3.5218
T5S0662	Gelsemine	509-15-9	-38.8807	-37.2353	-1.6454	6.7673
T0707	Cyclophosphamide monohydrate	6055-19-2	-38.8778	-38.8627	-0.015	4.913
T0106	Methazolamide	554-57-4	-38.8731	-38.4049	-0.4682	1.4439
T3658	TAS102	733030-01-8	-38.8598	-37.6656	-1.1941	4.952
T1684	Nitisinone	104206-65-7	-38.8511	-37.6558	-1.1952	11.8893
T1298	Clofibrate	637-07-0	-38.8479	-37.4541	-1.3938	7.671
T0993	Methoxamine hydrochloride	61-16-5	-38.83	-34.4134	-4.4166	8.1608
T1529	Miglitol	72432-03-2	-38.8195	-35.035	-3.7845	3.2375
T5S1058	Triptonide	38647-11-9	-38.8093	-38.9551	0.1458	3.4048
T4504	Triclocarban	101-20-2	-38.7893	-37.8875	-0.9018	5.4365
T1513	Telbivudine	3424-98-4	-38.7534	-37.3389	-1.4146	9.1649
T3366	Cephalotaxine	24316-19-6	-38.7365	-39.7755	1.039	11.8078
T0838	Triclabendazole	68786-66-3	-38.7184	-37.8838	-0.8346	7.5795
T6253	Talazoparib	1207456-01-6	-38.7177	-38.8116	0.0939	9.234
T4570	Pipobroman	54-91-1	-38.7145	-38.77	0.0555	9.9239
T6648	Rotundine	483-14-7	-38.7078	-36.5165	-2.1913	5.1217
T2215	Ferulic Acid	1135-24-6	-38.6919	-33.5799	-5.112	3.0674
T1580	Desvenlafaxine	93413-62-8	-38.6916	-36.7669	-1.9247	11.049
T6355	(R)-baclofen	69308-37-8	-38.6902	-32.6662	-6.024	6.783
T0309	Ipriflavone	35212-22-7	-38.6814	-38.9677	0.2863	4.7372
T3357	17 alpha-propionate	19608-29-8	-38.6791	-38.4293	-0.2497	10.6599
T0867	Bufexamac	2438-72-4	-38.676	-33.2821	-5.3939	3.5991
T0018	Procainamide hydrochloride	614-39-1	-38.6712	-36.0001	-2.6711	6.4467
T6739	Zoledronic Acid	118072-93-8	-38.6703	-32.9213	-5.749	5.9014
TWP2911	Thymidine	50-89-5	-38.6646	-37.2934	-1.3711	4.5573
T7969	Dipyrithione	3696-28-4	-38.6586	-37.8386	-0.82	2.5485
T0016	Mexenone	1641-17-4	-38.6389	-39.4467	0.8077	3.6541

T3429	Protopine	130-86-9	-38.6385	-33.1715	-5.467	0
T6953	Prilocaine	721-50-6	-38.6258	-36.2209	-2.4049	4.9267
T1453	Phenformin hydrochloride	834-28-6	-38.6237	-34.4025	-4.2212	4.689
T0767	Sulfapyridine	144-83-2	-38.5868	-37.9647	-0.6221	10.9575
T1173	Tropisetron hydrochloride	105826-92-4	-38.5815	-37.3765	-1.2049	2.9184
T1199	Mitotan	53-19-0	-38.5374	-38.7117	0.1743	8.2666
T1425	Pemirolast potassium	100299-08-9	-38.5279	-34.1359	-4.3921	5.6894
T2843	Aloe-emodin	481-72-1	-38.5087	-36.1358	-2.3729	11.4464
T1167	Cyproterone acetate	427-51-0	-38.502	-36.8653	-1.6367	17.6449
T6835	Fenoldopam mesylate	67227-57-0	-38.4926	-36.643	-1.8495	2.8626
T1065	Baclofen	1134-47-0	-38.4916	-33.2235	-5.268	8.6594
T0129	Sanguinarine chloride	5578-73-4	-38.4868	-37.4729	-1.0138	0
T0468	Xylocaine	137-58-6	-38.4668	-39.4543	0.9876	5.6363
T1272	Cytarabine	147-94-4	-38.4623	-36.8657	-1.5966	7.2304
T2S0007	Sodium ferulic	24276-84-4	-38.4607	-33.1277	-5.3329	2.4968
T1506	Vidarabine	5536-17-4	-38.4512	-36.652	-1.7992	6.242
T2728	Limonin	1180-71-8	-38.4438	-37.8446	-0.5991	8.8846
T0909L	Nalidixic acid sodium salt	3374-05-8	-38.4272	-36.0848	-2.3424	7.7456
T2196	Fipronil	120068-37-3	-38.4231	-39.0081	0.585	5.8989
T0452	Dropropizine	17692-31-8	-38.4164	-36.7107	-1.7057	2.985
T0493	Tyrosine	60-18-4	-38.4124	-35.6359	-2.7765	4.4505
T0225	Pizotifen Malate	5189-11-7	-38.4027	-40.5439	2.1412	5.4188
T3039	Hederagenin	465-99-6	-38.3759	-37.4944	-0.8815	18.6947
T2223	Kinetin	525-79-1	-38.3672	-37.0144	-1.3528	3.3123
T3096	Potassium canrenoate	2181-04-6	-38.3588	-30.6404	-7.7184	10.2756
T2205	Theophylline-7-acetic acid	652-37-9	-38.3473	-35.9392	-2.4081	2.613
T0379	Risedronic Acid	105462-24-6	-38.3069	-31.9447	-6.3622	16.2657
T3369	Nuciferine	475-83-2	-38.2986	-38.3342	0.0355	6.3055
T2832	S-Isocorydine(+)	475-67-2	-38.28	-35.0526	-3.2274	10.1179
T0828	Bromhexine hydrochloride	611-75-6	-38.2781	-36.8944	-1.3837	6.9472
T1614	Hydrocortisone	50-23-7	-38.2708	-35.3711	-2.8997	17.7006
T0722	Ursolic acid	77-52-1	-38.2314	-33.8876	-4.3438	14.3809
T6652	Salbutamol Sulfate	51022-70-9	-38.227	-35.9187	-2.3083	5.7613
T4813	L-Citrulline	372-75-8	-38.2229	-32.5346	-5.6883	4.5944
T2917	Tetrahydropalmatine hydrochloride	6024-85-7	-38.222	-36.9693	-1.2527	9.0068
T1508	Decitabine	2353-33-5	-38.211	-35.3322	-2.8788	4.1838
T6623	Oxibendazole	20559-55-1	-38.2011	-38.1493	-0.0517	8.3233
T0736	Roxarsone	121-19-7	-38.1941	-14.8873	-23.3068	0.6212
T1152	Albendazole	54965-21-8	-38.1884	-38.4724	0.2839	4.0635
T0277	Orciprenaline sulfate	5874-97-5	-38.1838	-26.0841	-12.0997	1.6221
T2792	Glucosamine sulfate	29031-19-4	-38.1734	-19.3131	-18.8603	5.2478
T0943	Carbamazepine	298-46-4	-38.1732	-37.3088	-0.8645	7.4076
T0251	Gemcitabine	95058-81-4	-38.1541	-36.7076	-1.4465	4.6005
T0109	Molsidomine	25717-80-0	-38.153	-35.0315	-3.1215	1.2586
T6069	Gemcitabine HCl	122111-03-9	-38.1426	-36.6829	-1.4597	4.5585
T0080	Trapidil	15421-84-8	-38.1413	-38.493	0.3517	2.6954
T0909	Nalidixic acid	389-08-2	-38.114	-35.6357	-2.4783	7.634
T5963	Menadione sodium bisulfite	130-37-0	-38.0927	-34.8643	-3.2284	6.131

T1951	Asenapine Maleate	85650-56-2	-38.0889	-35.9059	-2.183	0
T0217	Levodropropizine	99291-25-5	-38.082	-36.3812	-1.7008	4.978
T1412	Loxapine succinate salt	27833-64-3	-38.0787	-37.7599	-0.3188	3.9145
T1479	Isosorbide dinitrate	87-33-2	-38.0773	-36.165	-1.9123	9.177
T0269	Dichlorisone Acetate	79-61-8	-38.0631	-38.0471	-0.016	9.4377
T1488	Procarbazine hydrochloride	366-70-1	-38.0615	-38.1612	0.0997	2.2718
T0721	Piroctone olamine	68890-66-4	-38.0579	-32.3727	-5.6853	11.4505
T0353	Osalmid	526-18-1	-38.044	-36.8378	-1.2062	2.2178
T1882	Methylisoindigotin	97207-47-1	-38.0436	-37.5588	-0.4848	72.9429
TCP2830	Neryl acetate	141-12-8	-38.019	-37.1015	-0.9175	24.5245
T0499	Oxybenzone	131-57-7	-38.0181	-35.684	-2.3341	5.3846
T8400	Pamidronic acid	40391-99-9	-38.0089	-23.5744	-14.4345	4.0606
T1584	Terbutaline Sulfate	23031-32-5	-38.0072	-34.5077	-3.4995	4.4046
T6469	Dichlorphenamide	120-97-8	-37.9931	-36.8137	-1.1794	5.0676
T0293	Metyrapone	54-36-4	-37.9928	-38.4573	0.4645	9.5661
T1156	Palonosetron hydrochloride	135729-62-3	-37.9756	-38.4533	0.4776	15.3778
T1018	Prednisone	53-03-2	-37.9749	-36.1757	-1.7992	10.3122
T1404	Stavudine	3056-17-5	-37.9599	-38.3208	0.3609	2.3427
T2148	Carbidopa Hydrate	38821-49-7	-37.9528	-33.2848	-4.668	7.4236
T1532	Pidotimod	121808-62-6	-37.9434	-35.6603	-2.2831	13.9113
T1737	Genistein	446-72-0	-37.9412	-35.4541	-2.4871	4.0612
T3061	Lorlatinib	1454846-35-5	-37.9393	-38.3911	0.4518	1.1457
T6814	Cyproheptadine HCl	969-33-5	-37.9369	-40.3457	2.4087	5.1072
T0610	Piceatannol	10083-24-6	-37.9365	-36.7211	-1.2154	3.3914
T0863	Dendrid	54-42-2	-37.9363	-36.6856	-1.2507	9.3887
T0110	Zalcitabine	7481-89-2	-37.9229	-36.155	-1.7679	8.1584
T5S0814	Berberine hydrogen sulphate	633-66-9	-37.9099	-38.4386	0.5287	4.0504
T0037	Halazone	80-13-7	-37.908	-35.9588	-1.9492	4.8211
T0600	10,11-Dihydrocarbamazepine	3564-73-6	-37.9018	-37.4697	-0.4321	9.6148
T1561	Clobetasol propionate	25122-46-7	-37.9001	-35.9524	-1.9477	17.9995
T0948	Adrenalone hydrochloride	62-13-5	-37.8995	-35.0457	-2.8538	1.3154
T0066	Dichlorophen	97-23-4	-37.8974	-37.2971	-0.6002	5.1201
T1595	Nevirapine	129618-40-2	-37.8964	-37.6771	-0.2192	3.9013
T5033	Olprinone	106730-54-5	-37.8957	-37.2257	-0.67	3.7446
T2852	Citropten	487-06-9	-37.8914	-36.1885	-1.7029	3.1216
T1071L	DL-Adrenaline Hydrochloride	329-63-5	-37.8888	-35.3381	-2.5507	1.2156
T1435	Praziquantel	55268-74-1	-37.8802	-37.6924	-0.1878	5.8215
T3246	Tabersonine	4429-63-4	-37.88	-37.5863	-0.2937	11.529
T2738	Dehydroandrographolide	134418-28-3	-37.8424	-36.963	-0.8794	14.3883
T1652	Betamethasone	378-44-9	-37.818	-36.1355	-1.6825	14.7992
T0177	Homatropine Bromide	51-56-9	-37.8173	-36.5094	-1.308	7.6978
T2570	Menadiol Diacetate	573-20-6	-37.8122	-37.7459	-0.0663	10.7102
T2575	Guanethidine Monosulfate	645-43-2	-37.8012	-34.3259	-3.4753	6.8115
T0427	Homatropine Methylbromide	80-49-9	-37.7686	-27.1964	-10.5722	7.5775
T3033	Deoxycorticosterone acetate	56-47-3	-37.7328	-37.1377	-0.5951	5.9285
T1431	Khellin	82-02-0	-37.7287	-36.8688	-0.86	6.5141
T1055	Ifosfamide	3778-73-2	-37.7232	-36.4508	-1.2724	2.8826
T0354	Canrenone	976-71-6	-37.7162	-38.7201	1.0038	0

T1183	Retinol	68-26-8	-37.7144	-37.7107	-0.0037	9.2948
T1480	Danazol	17230-88-5	-37.7143	-37.4342	-0.2801	3.5739
T2221	Uridine	58-96-8	-37.7053	-36.3079	-1.3974	3.551
T1428	Trifluridine	70-00-8	-37.6997	-36.6008	-1.0989	11.6007
T1567	Olanzapine	132539-06-1	-37.6784	-36.9129	-0.7655	1.7522
T0236	Azatadine Maleate	3978-86-7	-37.6684	-36.0261	-1.6423	5.5854
T1026	Mafenide hydrochloride	138-37-4	-37.6592	-31.9634	-5.6959	2.3487
T3602	D-Pantothenic acid sodium salt	867-81-2	-37.6573	-33.4811	-4.1761	6.9211
T1144L	Lidocaine Hydrochloride hydrate	6108-05-0	-37.6486	-38.3248	0.6762	8.6468
T0729	Benzyl benzoate	120-51-4	-37.6464	-36.9286	-0.7177	5.4349
T4590	L-KAWAIN	500-64-1	-37.6415	-37.1842	-0.4573	3.763
T1591	Cyclocytidine hydrochloride	10212-25-6	-37.6124	-36.2671	-1.3453	0.4626
TN1545	D-Tyrosine	556-02-5	-37.6116	-25.2508	-12.3608	5.996
T1556	Ornidazole	16773-42-5	-37.5997	-34.7879	-2.8118	3.9117
T4513	Vanillyl butyl ether	82654-98-6	-37.596	-36.9183	-0.6776	5.3779
T1071	L-Adrenaline	51-43-4	-37.5942	-35.0546	-2.5396	2.691
T1103	Aminoglutethimide	125-84-8	-37.5698	-37.8428	0.273	20.7836
T1339	5-Azacytidine	320-67-2	-37.5579	-36.3986	-1.1593	3.8201
T8462	Enoximone	77671-31-9	-37.5549	-37.9462	0.3913	2.1666
T5S2017	Ingenol	30220-46-3	-37.5505	-35.1664	-2.3842	7.2692
T0107	Halcinonide	3093-35-4	-37.55	-36.9307	-0.6194	15.9253
T8132	Iron sucrose	8047-67-4	-37.5476	-28.8344	-8.7132	4.679
T0174	Cyproheptadine hydrochloride	41354-29-4	-37.5323	-39.5231	1.9908	5.1223
T0730	Triacetin	102-76-1	-37.4682	-36.3084	-1.1598	7.3474
T2195	Epiandrosterone	481-29-8	-37.4625	-37.3479	-0.1145	0.0594
T0705	Triflusal	322-79-2	-37.4454	-37.6498	0.2045	5.0996
T3551	Calcium pantothenate	137-08-6	-37.4359	-30.6755	-6.7603	9.6002
T0949	Amstat	1197-18-8	-37.4331	-16.8108	-20.6223	2.917
T0558	Hydroferulic acid	1135-23-5	-37.4274	-35.3255	-2.1019	2.3879
T4993	Afloqualone	56287-74-2	-37.3951	-36.9425	-0.4526	5.9016
T0477	Zileuton	111406-87-2	-37.3908	-32.689	-4.7018	4.8876
T1476	Pramipexole	104632-26-0	-37.3891	-34.8771	-2.5119	6.5729
T0792	(-)-Sparteine sulfate pentahydrate	6160-12-9	-37.3828	-22.5062	-14.8766	0
T1238	Daidzein	486-66-8	-37.3632	-36.5696	-0.7935	5.0207
T1573	Flunisolide	3385-03-3	-37.3623	-35.2355	-2.1269	14.1016
T1430	Betamipron	3440-28-6	-37.3418	-32.2684	-5.0734	1.5471
T1721	2'-Deoxyuridine	951-78-0	-37.3364	-36.8698	-0.4667	8.6912
T0461	Berberine hydrochloride	633-65-8	-37.3355	-38.4627	1.1271	3.7819
T0456	Cinchophen	132-60-5	-37.328	-36.127	-1.201	5.8844
T1171	Methylprednisolone	83-43-2	-37.306	-37.1848	-0.1212	16.9
T0977	Medrysone	2668-66-8	-37.3002	-36.835	-0.4652	9.9783
T2179	Triptolide	38748-32-2	-37.2859	-38.4284	1.1425	7.7618
T2150	Guanfacine hydrochloride	29110-48-3	-37.2756	-35.7152	-1.5603	6.4661
T3729	Ethyl gallate	831-61-8	-37.2355	-33.1639	-4.0716	1.0322
T2220	2-Methoxyestradiol	362-07-2	-37.2354	-36.4487	-0.7867	2.6011
T6580	Meprednisone	1247-42-3	-37.2203	-35.7105	-1.5099	11.1765
T2397	Topiroxostat	577778-58-6	-37.2189	-36.4135	-0.8054	1.9351
T2907	Tanshinone I	568-73-0	-37.2173	-37.5734	0.3561	0

T8122	Mafenide Acetate	13009-99-9	-37.216	-32.7938	-4.4222	2.1415
T0739	Guaifenesin	93-14-1	-37.1899	-35.2872	-1.9027	5.4498
T1068	Anthralin	1143-38-0	-37.1875	-35.7012	-1.4864	11.3228
T3017	Trenbolone acetate	10161-34-9	-37.1605	-36.8456	-0.315	2.9678
T6188	Desvenlafaxine succinate hydrate	386750-22-7	-37.1452	-35.2322	-1.913	10.5625
T1666	Fludrocortisone acetate	514-36-3	-37.1209	-35.9801	-1.1408	15.6606
T0201	Meptazinol hydrochloride	59263-76-2	-37.118	-36.8624	-0.2557	7.6391
T0505	L-(-)-??-Methyldopa	555-30-6	-37.1058	-19.5182	-17.5875	4.5637
T4987	Calcium ??-D-heptagluconate hydrate	17140-60-2	-37.0641	-36.3397	-0.7244	8.6471
T1481	Fudosteine	13189-98-5	-37.035	-32.4911	-4.5438	1.7783
T2793	Tetrahydropalmatine	2934-97-6	-37.0325	-30.7486	-6.2839	6.3784
T2865	Oleanolic Acid	508-02-1	-37.0225	-36.9539	-0.0687	14.1292
T3264	Etilefrine hydrochloride	943-17-9	-37.0217	-24.2585	-12.7633	2.2295
T3616	Carbazochrome	69-81-8	-37.0172	-33.3698	-3.6474	2.471
T6S1256	Ruscogenin	472-11-7	-37.0029	-36.2269	-0.776	4.2831
T2137	Ibudilast	50847-11-5	-36.999	-36.5776	-0.4214	7.362
T12478	Pinacidil monohydrate	85371-64-8	-36.9921	-36.4124	-0.5797	12.4846
T0851	Pregnenolone	145-13-1	-36.9915	-36.2954	-0.696	7.6556
T1337	Pamidronate disodium salt	57248-88-1	-36.9914	-22.8512	-14.1402	3.4656
T2750	Ginkgolide C	15291-76-6	-36.9575	-37.6536	0.6961	28.303
T2800	L-Theanine	3081-61-6	-36.9517	-31.4007	-5.551	3.9571
T6576	Malotilate	59937-28-9	-36.9415	-37.1475	0.206	13.9284
T2869	Emodin	518-82-1	-36.9242	-33.6897	-3.2346	11.256
T4498	Stiripentol	49763-96-4	-36.9215	-36.793	-0.1285	8.6007
T3020	Sclareol	515-03-7	-36.9108	-36.5288	-0.382	18.0321
T0071	Homosalate	118-56-9	-36.9094	-36.9746	0.0652	2.1972
T1642	Lenalidomide	191732-72-6	-36.9051	-36.1874	-0.7177	4.7156
T4S0797	Berberine	2086-83-1	-36.9011	-37.8506	0.9495	3.992
T1262	Tazobactam acid	89786-04-9	-36.8994	-31.316	-5.5835	9.3519
T1159	Leflunomide	75706-12-6	-36.8762	-37.0373	0.161	3.3522
T0996	Ethynodiol diacetate	297-76-7	-36.8745	-36.8268	-0.0477	13.092
T0411	Calcium gluconate	299-28-5	-36.8727	-32.4229	-4.4498	2.9151
T3948	Hendecanoic acid	112-37-8	-36.8638	-30.8187	-6.0451	5.4495
T0808	Diflunisal	22494-42-4	-36.8515	-33.1539	-3.6976	5.1979
T0751	Furazolidone	67-45-8	-36.8375	-36.2889	-0.5487	1.7102
T0891	4,4'-Diaminodiphenylsulfone	80-08-0	-36.8101	-37.6487	0.8385	3.4982
T0200	Thioctic acid	1077-28-7	-36.7942	-30.523	-6.2712	2.4233
T5S0045	Isofraxidin	486-21-5	-36.7817	-35.0106	-1.7711	3.0233
T0160	Anagrelide	68475-42-3	-36.746	-34.4326	-2.3134	0
T4507	Chlorphenesin	104-29-0	-36.7357	-34.117	-2.6187	1.3709
T0848	Levodopa	59-92-7	-36.7352	-34.62	-2.1152	6.6047
T0439	L-Tryptophan	73-22-3	-36.7333	-31.7127	-5.0206	3.1823
T7650	Resazurin sodium salt	62758-13-8	-36.7314	-27.796	-8.9354	0
T2847	Indigo	482-89-3	-36.7087	-36.1425	-0.5662	97.3022
T3227	Danshensu	76822-21-4	-36.6964	-31.7908	-4.9056	2.4032
T1243	Hydrocortisone acetate	50-03-3	-36.6902	-34.9489	-1.7412	17.3953
T0798	Triamcinolone	124-94-7	-36.6889	-36.1574	-0.5315	13.0699
T1041	Risedronate Sodium	115436-72-1	-36.6653	-27.7109	-8.9544	6.6398

T7853	Pachycarpine	492-08-0	-36.6557	-33.087	-3.5688	0
T0446	Naphazoline hydrochloride	550-99-2	-36.6393	-35.7522	-0.8872	3.6274
T0753	Salicylanilide	87-17-2	-36.635	-34.6592	-1.9758	1.8802
T2563	O-Acetyl-L-carnitine hydrochloride	5080-50-2	-36.6204	-33.1573	-3.4631	9.6847
T7534	Teriflunomide	163451-81-8	-36.5984	-28.546	-8.0524	7.3304
T1686	Hematoxylin	517-28-2	-36.5976	-35.6124	-0.9852	1.5538
T0843	Ademine	396-01-0	-36.5968	-36.9005	0.3037	1.8783
T1150	Bupropion hydrochloride	31677-93-7	-36.5908	-35.4322	-1.1585	6.8375
T0314	Hexylresorcinol	136-77-6	-36.5684	-36.3008	-0.2675	7.4373
T3124	Carbendazim	10605-21-7	-36.5292	-35.8775	-0.6517	5.7537
T0134	Imiquimod	99011-02-6	-36.5124	-35.8908	-0.6216	4.5449
T3270	Dihydralazine sulphate	7327-87-9	-36.5092	-35.4326	-1.0765	3.4743
T2889	Isoferulic acid	537-73-5	-36.5074	-28.7466	-7.7608	1.8207
T6390	Anagrelide HCl	58579-51-4	-36.5047	-35.0889	-1.4159	0
T1612	Hodostin	51-60-5	-36.4903	-34.8785	-1.6119	10.9119
T0448	Dexibuprofen	51146-56-6	-36.4853	-30.2405	-6.2448	6.5711
T1440	Ethacridine lactate monohydrate	6402-23-9	-36.4579	-34.4211	-2.0368	1.7714
T6216	Abiraterone	154229-19-3	-36.4477	-36.3798	-0.0679	14.8656
T6951	Pramipexole 2HCl Monohydrate	191217-81-9	-36.4453	-35.7793	-0.666	1.8523
T0946	Mepivacaine hydrochloride	1722-62-9	-36.4345	-35.2745	-1.16	5.9983
T0927	Butylparaben	94-26-8	-36.4331	-35.9655	-0.4676	3.3793
T0290	Tizanidine hydrochloride	64461-82-1	-36.4054	-34.9331	-1.4722	3.3066
T1009	Aquacrine	53-16-7	-36.3993	-36.2409	-0.1584	0.0278
T0416	Sodium gluconate	527-07-1	-36.3968	-34.2104	-2.1864	4.2781
T0682	Lamivudine	134678-17-4	-36.3856	-36.3189	-0.0666	8.0336
T6488	Epinastine HCl	108929-04-0	-36.3802	-35.3048	-1.0754	0
T0287	Hexamethonium Bromide	55-97-0	-36.3475	-32.7606	-3.587	5.733
T0137	Mirtazapine	85650-52-8	-36.3275	-35.6019	-0.7257	0
T3044	Teriflunomide	108605-62-5	-36.3183	-28.7	-7.6183	4.4323
T1624	Neostigmine bromide	114-80-7	-36.3126	-35.0656	-1.247	9.7607
T0829	Etofylline	519-37-9	-36.2772	-35.8803	-0.3969	3.1781
T2384	Pomalidomide	19171-19-8	-36.2634	-35.9271	-0.3362	5.4059
T2944	Sodium Danshensu	67920-52-9	-36.2578	-33.1147	-3.1431	1.4573
T4682	Allopregnanolone	516-54-1	-36.2435	-35.7121	-0.5314	3.8971
T6507	Gabapentin HCl	60142-95-2	-36.2431	-27.7184	-8.5247	8.8841
T2995	Chrysophanic Acid	481-74-3	-36.2281	-36.0129	-0.2151	10.8188
T0926	Azelaic acid	123-99-9	-36.2194	-26.6485	-9.571	2.9452
T5967	Ethacridine lactate	1837-57-6	-36.1818	-33.9544	-2.2274	1.5688
T1482	Ciclopirox	29342-05-0	-36.1429	-29.8691	-6.2738	3.9473
T5039	Sodium 4-aminohippurate Hydrate	94-16-6	-36.1099	-32.0479	-4.062	1.1085
T1548	8-Methoxypsoralen	298-81-7	-36.0998	-34.9093	-1.1906	1.1973
T5607	Geranyl acetate	105-87-3	-36.0977	-36.2052	0.1075	8.9785
T2803	Monocrotaline	315-22-0	-36.0927	-34.3899	-1.7028	61.6785
T6169	Indirubin	479-41-4	-36.0894	-36.014	-0.0753	42.7334
T19288	DL-Arginine	7200-25-1	-36.084	-30.5828	-5.5012	4.3481
T0689	D-Phenylalanine	673-06-3	-36.0676	-31.3003	-4.7673	2.4834
T3S1866	Propyl gallate	121-79-9	-36.0675	-34.2696	-1.7979	3.746
T5027	Dimemorfan phosphate	36304-84-4	-36.0584	-34.5253	-1.5331	0

T6006	Romidepsin	128517-07-7	-36.0476	-35.5452	-0.5024	23.0123
T0233	Ethamivan	304-84-7	-36.0382	-35.3032	-0.735	10.4302
T1436	4-Aminohippuric Acid	61-78-9	-36.0316	-34.7161	-1.3155	1.9954
T5012	Ethoxzolamide	452-35-7	-36.0146	-34.6229	-1.3917	0.4637
T0376	Crotamiton	483-63-6	-36.0135	-35.4026	-0.6109	10.4296
T0556	6-Methoxy-2-naphthoic acid	2471-70-7	-35.9995	-34.465	-1.5345	3.4272
T3091	Carmustine	154-93-8	-35.9991	-35.7289	-0.2702	5.7322
T1442	Pizotifen	15574-96-6	-35.9794	-34.3636	-1.6158	4.4436
T2941	Glucosamine hydrochloride	66-84-2	-35.978	-18.6026	-17.3754	0.5835
T0429	Glucosamine	3416-24-8	-35.9703	-18.2222	-17.7481	0.5041
T0936	Aminophenazone	58-15-1	-35.9672	-36.0017	0.0345	5.8123
T0964	Floxuridine	50-91-9	-35.9608	-34.3079	-1.6529	8.1608
T4549	Tibolone	5630-53-5	-35.937	-35.732	-0.205	3.077
T1249	Beclamide	501-68-8	-35.9327	-35.8104	-0.1223	7.2298
T0755	Carbaglu	1188-38-1	-35.932	-23.078	-12.8539	2.8651
T1076	Dexamethasone	50-02-2	-35.9262	-34.4477	-1.4785	13.9389
T0267	Zonisamide	68291-97-4	-35.9172	-34.6707	-1.2465	1.4133
T1028	Moxonidine	75438-57-2	-35.8859	-34.7199	-1.1659	3.1293
T4693	2-APB	524-95-8	-35.882	-31.5937	-4.2883	0.6251
T1241	Altretamine	645-05-6	-35.8768	-35.9538	0.077	3.2494
T6214	Emtricitabine	143491-57-0	-35.8631	-35.1926	-0.6705	2.3586
T1314	Triclosan	3380-34-5	-35.8324	-35.5093	-0.3231	2.069
T0856L	Dehydroepiandrosterone	53-43-0	-35.8304	-35.7169	-0.1135	0.0362
T5040	Medroxyprogesterone	520-85-4	-35.8253	-34.1982	-1.6271	9.5261
T8057	Citronellyl acetate	150-84-5	-35.8126	-35.9413	0.1287	5.7113
T1114	Ciclopirox ethanolamine	41621-49-2	-35.7907	-29.1482	-6.6425	3.9352
T7216	Mirogabalin	1138245-13-2	-35.7695	-22.2685	-13.501	7.7829
T6491	Equol	531-95-3	-35.7651	-34.0936	-1.6716	4.1768
T4550	Ajmaline	4360-12-7	-35.7579	-30.7917	-4.9662	5.8682
T0383	Fenspiride hydrochloride	5053-08-7	-35.7436	-28.1841	-7.5594	1.4941
T6S0107	Peimine	23496-41-5	-35.7398	-36.2573	0.5175	2.2856
T0301	Ethisterone	434-03-7	-35.737	-35.4949	-0.2422	2.7563
T1541	Drospirenone	67392-87-4	-35.7313	-36.3492	0.6179	0
T0284	Sulfogaiacol	1321-14-8	-35.7222	-33.831	-1.8912	2.5401
T0360	chlophenadione	1146-99-2	-35.722	-35.9545	0.2325	5.8915
T2718	Palmatine chloride	10605-02-4	-35.7116	-33.4615	-2.2501	6.2299
T1216	Diethylstilbestrol	56-53-1	-35.7064	-35.8216	0.1152	10.2245
T0332	D-Sorbitol	50-70-4	-35.7016	-32.8743	-2.8273	4.6224
T0948L	Corticosterone	50-22-6	-35.6992	-33.5203	-2.1788	10.1422
T2577	Dienogest	65928-58-7	-35.6911	-36.2216	0.5305	5.6423
T2A2498	lysine	56-87-1	-35.6742	-29.8999	-5.7743	3.7699
T1120	Dacarbazine	4342-03-4	-35.667	-35.0605	-0.6065	2.5486
T4823	D-(-)-Fructose	57-48-7	-35.6352	-32.9197	-2.7155	3.0946
T5323	MD 69276	29218-27-7	-35.635	-35.1799	-0.4551	0.9437
T0451	Minoxidil	38304-91-5	-35.633	-34.7301	-0.903	2.4566
T0744	Zinc undecylenate	557-08-4	-35.6197	-29.6768	-5.9429	8.0055
T0275	Meticrane	1084-65-7	-35.5981	-34.7602	-0.8379	5.7763
T7063	Oxymetholone	434-07-1	-35.5848	-26.8456	-8.7392	2.9992

T1571	Estriol	50-27-1	-35.5743	-35.173	-0.4013	0.76
T0414	D-Mannitol	69-65-8	-35.5549	-32.2305	-3.3244	3.6818
T0382	Eprodisate disodium	36589-58-9	-35.5532	-27.7429	-7.8103	3.6126
T4000	(??)-Equol	94105-90-5	-35.5509	-33.8309	-1.72	4.0774
T8071	L-Lysine hydrochloride	657-27-2	-35.5332	-29.6716	-5.8616	1.9065
T4S0647	Butylphthalide	6066-49-5	-35.5309	-34.4553	-1.0755	5.9299
T3746	Dimethyl phthalate	131-11-3	-35.5197	-35.2938	-0.2258	18.4213
T6863	Isosorbide Mononitrate	16051-77-7	-35.4979	-33.5705	-1.9273	1.6182
T2729	Scoparone	120-08-1	-35.4945	-35.1301	-0.3644	2.34
T3277	Tulobuterol hydrochloride	56776-01-3	-35.4511	-33.1969	-2.2543	4.2873
T2567	Etonogestrel	54048-10-1	-35.4155	-34.2608	-1.1547	12.6515
T0783	Mianserin hydrochloride	21535-47-7	-35.4062	-34.0263	-1.3799	0
T1261	Medroxyprogesterone Acetate	71-58-9	-35.4047	-34.7624	-0.6423	10.0528
T6466	Dexmedetomidine HCl	145108-58-3	-35.3968	-35.1489	-0.2479	6.1232
T0804	(+)-Pilocarpine hydrochloride	54-71-7	-35.3954	-34.8298	-0.5656	13.6315
T6888	Methylene Blue	61-73-4	-35.3947	-33.4715	-1.9231	4.6434
T0390	Alkomide	3011-89-0	-35.3874	-34.4294	-0.958	1.3733
T1178	Temozolomide	85622-93-1	-35.3577	-34.9317	-0.426	0.3835
T0800	Antrapurol	117-10-2	-35.3494	-34.2858	-1.0636	10.7698
T6503	Fluorometholone Acetate	3801-06-7	-35.3051	-35.3129	0.0078	17.8923
T0310	Actarit	18699-02-0	-35.2694	-28.1872	-7.0822	3.5906
T2909	Fraxetin	574-84-5	-35.2638	-33.8275	-1.4362	1.9172
T3316	Fluoromethalone	426-13-1	-35.2575	-34.2555	-1.002	11.5827
T1231	Pilocarpine nitrate	148-72-1	-35.2524	-35.2844	0.0319	6.1089
T1047	Epinephrine bitartrate	51-42-3	-35.2349	-33.6933	-1.5416	1.0499
T1558	Resveratrol	501-36-0	-35.2283	-34.7951	-0.4332	2.86
T3121	Betulin	473-98-3	-35.2109	-35.1724	-0.0385	12.6755
T2984	Scopoletin	92-61-5	-35.2065	-34.7005	-0.5061	1.0337
T2597	Norgestrel	6533-00-2	-35.1946	-35.6445	0.4499	12.7907
T7223	Gestrinone	16320-04-0	-35.1936	-34.8694	-0.3241	9.9426
T2143	Bergapten	484-20-8	-35.1701	-34.8656	-0.3045	2.0079
T0061	Clofibric acid	882-09-7	-35.1437	-30.6249	-4.5188	7.5917
T0195	Secnidazole	3366-95-8	-35.1172	-34.335	-0.7822	5.608
T3377	phenylalanine	63-91-2	-35.1077	-32.1184	-2.9893	4.4094
T0849	Pyrimethamine	58-14-0	-35.0813	-35.1492	0.0679	6.4913
T0959	Ethopabate	59-06-3	-35.0801	-33.7304	-1.3497	4.5593
T1473	Trilostane	13647-35-3	-35.0667	-33.9789	-1.0878	0.9067
T4826	L-Histidine monohydrochloride monohydrat	5934-29-2	-35.063	-24.3031	-10.7599	4.5433
T1437	Dexpanthenol	81-13-0	-35.0474	-33.9628	-1.0845	8.461
T0086	Galanthamine HBr	1953-04-4	-35.0332	-34.6391	-0.3941	1.1585
T0525	Flopropione	2295-58-1	-35.0325	-31.2772	-3.7553	1.8919
T0489	Flutamide	13311-84-7	-35.028	-35.4395	0.4115	9.343
T6422	Brimonidine Tartrate	70359-46-5	-34.9991	-33.5285	-1.4706	1.7996
T1329	Chlorophos	52-68-6	-34.9561	-34.4005	-0.5555	4.4649
T0241	Sulfanilylurea	547-44-4	-34.9178	-36.1994	1.2816	2.7745
T0362	Ticlopidine hydrochloride	53885-35-1	-34.8834	-33.1683	-1.715	1.9918
T3718	??-santonin	481-06-1	-34.8588	-35.4776	0.6188	0
T5010	Propyphenazone (4-Isopropylantipyrine)	479-92-5	-34.8499	-34.5973	-0.2527	11.6489

T0006	Methylene Blue trihydrate	7220-79-3	-34.8468	-33.2897	-1.5571	6.7335
T0087L	Sulfacetamide sodium	127-56-0	-34.8413	-32.3572	-2.4841	9.978
T0279	Metaraminol bitartrate	33402-03-8	-34.8315	-29.6593	-5.1723	3.2604
T5557	Propylparaben	94-13-3	-34.8249	-34.1193	-0.7056	2.5989
T1600	Doxifluridine	3094-09-5	-34.8165	-33.5164	-1.3001	2.6057
T1424	Ethynyl estradiol	57-63-6	-34.8047	-34.8075	0.0029	2.9204
T0769	Aceglutamide	2490-97-3	-34.7915	-34.2984	-0.493	3.2076
T0248	Clorprenaline hydrochloride	6933-90-0	-34.7733	-31.401	-3.3724	4.2243
T5623	Bisphenol A	80-05-7	-34.7598	-31.346	-3.4138	11.5273
T5174	Nestoron	7759-35-5	-34.7516	-34.7397	-0.0119	14.9347
T0398	Carzenide	138-41-0	-34.7475	-34.9299	0.1824	1.5824
T3744	Veratric acid	93-07-2	-34.7468	-33.2083	-1.5385	2.8047
T6641	Rilmenidine Phosphate	85409-38-7	-34.7401	-34.921	0.1809	6.4726
T0042	Xylometazoline hydrochloride	1218-35-5	-34.694	-33.3679	-1.3262	12.3333
T0213	Thalidomide	50-35-1	-34.6892	-34.7708	0.0816	8.5465
T2884	Cortisone	53-06-5	-34.6858	-33.4739	-1.2119	5.6881
T7160	Lofexidine	31036-80-3	-34.6717	-33.8244	-0.8473	6.3536
T2524	Dexmedetomidine	113775-47-6	-34.6663	-34.805	0.1387	7.5968
T6579	Medetomidine HCl	86347-15-1	-34.6352	-34.4763	-0.1589	6.75
T1096	Milrinone	78415-72-2	-34.6267	-34.0432	-0.5835	4.2398
T1507	Streptozocin	18883-66-4	-34.5985	-34.3923	-0.2062	3.5267
T0916	Butyl 4-aminobenzoate	94-25-7	-34.5819	-34.5183	-0.0636	1.9295
T3339	Sophoridine	6882-68-4	-34.5797	-32.4052	-2.1745	0
T0015	Mephenesin	59-47-2	-34.5717	-32.09	-2.4816	2.4953
T0870	Altrenogest	850-52-2	-34.5643	-34.0787	-0.4856	7.9651
T2559	Allylestrenol	432-60-0	-34.5543	-34.8163	0.262	6.633
T21066	Norepinephrine bitartrate salt	3414-63-9	-34.5314	-28.9118	-5.6196	2.9884
T5886	Benzenebutyric acid	1821-12-1	-34.5207	-32.8864	-1.6342	2.8842
T0333	Aniracetam	72432-10-1	-34.484	-33.2843	-1.1997	4.5805
T6548	Irsogladine	57381-26-7	-34.468	-34.2205	-0.2474	1.6109
T6688	Synephrine HCl	5985-28-4	-34.4632	-32.73	-1.7332	1.1043
T2A2532	histidine	71-00-1	-34.4513	-24.8873	-9.564	3.3021
T5327	Amezinium (methylsulfate)	30578-37-1	-34.4342	-32.9713	-1.4629	30.4542
T1263	Levonorgestrel	797-63-7	-34.4253	-34.3021	-0.1232	12.7903
T3736	Olivetol	500-66-3	-34.408	-33.7188	-0.6892	7.5407
T4815	Hippuric acid	495-69-2	-34.3914	-33.2284	-1.163	1.8115
T2876	Flavone	525-82-6	-34.3867	-33.5746	-0.8121	0.8953
T8365	Methenamine Hippurate	5714-73-8	-34.3827	-33.0596	-1.323	1.6509
T4S1725	Galanthamine	357-70-0	-34.3826	-33.8432	-0.5394	38.9924
T7044	Norepinephrine	51-41-2	-34.3766	-29.0513	-5.3253	3.0651
T7197	Fadrozole	102676-47-1	-34.3683	-33.851	-0.5173	2.1046
T0823	Thioctamide	3206-73-3	-34.3588	-35.6238	1.265	4.6399
T2A2497	glutamic acid	56-86-0	-34.3147	-21.591	-12.7237	4.3853
T0478	Progesterone	57-83-0	-34.2919	-33.9368	-0.355	4.3131
T1560	Prednisolone	50-24-8	-34.2717	-34.3839	0.1122	15.0714
T0453	Phenylephrine hydrochloride	61-76-7	-34.2693	-29.6601	-4.6092	1.0925
T2807	Caffeic Acid	331-39-5	-34.2527	-33.109	-1.1437	2.6657
T7556	Fadrozole hydrochloride	102676-31-3	-34.2294	-33.6176	-0.6119	1.8509

T0087	Sulfacetamide sodium salt hydrate	6209-17-2	-34.217	-31.9162	-2.3008	7.1134
T0825	Ebselen	60940-34-3	-34.1941	-34.1941	0	2.4336
T2209	Serotonin hydrochloride	153-98-0	-34.1863	-30.8151	-3.3713	1.7617
T0821	Iproniazid Phosphate	305-33-9	-34.1736	-33.764	-0.4096	2.0098
T5559	Carboxine	5234-68-4	-34.1713	-34.2522	0.0809	2.0224
T1048	Estradiol	50-28-2	-34.1314	-33.9492	-0.1822	0.8984
T6871	L-Glutamic acid monosodium salt	142-47-2	-34.1304	-19.9142	-14.2162	3.3614
T1386	Phenazopyridine hydrochloride	136-40-3	-34.1072	-33.7296	-0.3776	1.1754
T0400	Nitroxoline	4008-48-4	-34.0831	-32.6305	-1.4526	2.0302
T0594	Diphemanil mesylate	62-97-5	-34.068	-35.6881	1.6201	4.4064
T0856	Dehydroepiandrosterone acetate	853-23-6	-34.062	-33.9808	-0.0811	3.9765
T2811	Harmine hydrochloride	343-27-1	-34.0253	-33.0655	-0.9597	1.2461
T2736	Sodium Demethylcantharidate	13114-29-9	-34.0241	-26.9117	-7.1124	10.8002
T1601	Lomustine	13010-47-4	-34.0042	-33.9594	-0.0448	5.8219
T1490	Gestodene	60282-87-3	-34.0039	-33.8767	-0.1272	12.3905
T1579	Mestranol	72-33-3	-33.9975	-34.1055	0.108	4.4022
T5471	GUANABENZ	5051-62-7	-33.9793	-30.9896	-2.9897	2.6482
T1046	Mexiletine hydrochloride	5370-01-4	-33.9755	-28.8276	-5.1479	9.3191
T2207	Adrenosterone	382-45-6	-33.9744	-33.8723	-0.1022	0
T0790	Carbaryl	63-25-2	-33.9729	-33.9067	-0.0663	7.4898
T1587	Exemestane	107868-30-4	-33.9296	-33.9178	-0.0118	0
T0813	Acetazolamide	59-66-5	-33.9179	-33.3419	-0.576	3.9063
T3S2100	Securinine	5610-40-2	-33.9172	-35.1431	1.226	0
T1535	Sodium Phenylbutyrate	1716-12-7	-33.9155	-32.2301	-1.6854	3.5204
T0259	Chromocarbe	4940-39-0	-33.908	-31.2925	-2.6155	0.2337
T5264	Androsterone	53-41-8	-33.8927	-33.6225	-0.2702	0.0549
TJO2758	D-Glucuronic acid	6556-12-3	-33.8821	-30.0878	-3.7943	1.3541
T3S2019	Lathyrol	34420-19-4	-33.8586	-33.2166	-0.642	18.7977
T1285	Anethole trithione	532-11-6	-33.8186	-33.418	-0.4006	18.1978
T0048	Nikethamide	59-26-7	-33.808	-32.9742	-0.8338	13.0023
T0871	Noradrenaline bitartrate	69815-49-2	-33.7675	-28.5468	-5.2207	2.7311
T1064	Noradrenaline bitartrate monohydrate	108341-18-0	-33.7675	-28.5468	-5.2207	2.7311
T1390	Panthenol	16485-10-2	-33.7633	-32.3768	-1.3865	8.572
T3327	Arteether	75887-54-6	-33.758	-32.0337	-1.7243	4.7083
T1514	Norethindrone	68-22-4	-33.7435	-33.5671	-0.1764	2.967
T0300	Pargyline	555-57-7	-33.7199	-31.1359	-2.584	3.032
T1673	Acamprosate calcium	77337-73-6	-33.6936	-23.6344	-10.0592	2.529
T1364	Cefalexine EP IMpurity B	22252-43-3	-33.6875	-31.8285	-1.859	2.9138
T1079	Metronidazole	443-48-1	-33.6801	-32.9906	-0.6895	2.5762
TJS0314	4-Methyllimetol	2107-76-8	-33.6788	-32.9363	-0.7425	4.5632
T5271	5-Methoxytryptamine	608-07-1	-33.6621	-31.7971	-1.865	2.0078
T0262	Dextromethorphan HBr monohydrate	6700-34-1	-33.6592	-33.2247	-0.4345	1.7916
T0164	Oxaliplatin	61825-94-3	-33.6438	-11.6588	-21.985	0
T0126	Diazoxide	364-98-7	-33.6421	-34.5402	0.8981	0
T3947	Nonanoic acid	112-05-0	-33.6239	-28.9918	-4.6321	3.6492
T0256	Lithium citrate tribasic tetrahydrate	6080-58-6	-33.6083	-30.6329	-2.9754	10.0047
T3605	Gastrodenol	57644-54-9	-33.6083	-30.6329	-2.9754	10.0047
T1108	Nithiamide	140-40-9	-33.6047	-32.5449	-1.0598	1.3637

T1083L	Theophylline monohydrate	5967-84-0	-33.5831	-33.0476	-0.5355	0
T1322	Bendazol	621-72-7	-33.5699	-32.8058	-0.7641	3.6159
T2203	L-Carnitine hydrochloride	10017-44-4	-33.545	-27.2073	-6.3377	2.6887
T1681	Aminophylline	317-34-0	-33.5163	-32.9105	-0.6058	0
T1124	Flumethasone	2135-17-3	-33.5087	-33.1451	-0.3636	12.4915
T5653	Cinnamyl acetate	103-54-8	-33.5046	-33.5319	0.0273	5.3194
T1287	Synephrine	94-07-5	-33.5039	-29.3336	-4.1703	2.3527
T0050	4-Aminoantipyrine	83-07-8	-33.496	-33.564	0.068	2.4681
T2735	5-Acetylsalicylic acid	13110-96-8	-33.4508	-32.3132	-1.1375	2.7923
T3089	Thioguanine	154-42-7	-33.4021	-29.497	-3.9051	0.0642
T5002	Methyl Aminolevulinate Hydrochloride	79416-27-6	-33.4015	-28.46	-4.9415	4.9303
T0479	Cytisine	485-35-8	-33.3994	-31.2006	-2.1988	0
T1265	Amrinone	60719-84-8	-33.3955	-32.65	-0.7454	1.5315
T5050	Selegiline hydrochloride	14611-52-0	-33.3925	-31.2238	-2.1687	8.8456
T2900	Paeonol	552-41-0	-33.3912	-31.5903	-1.8009	4.1517
T3378	17??-Estradiol	57-91-0	-33.3602	-32.9598	-0.4004	0.9431
T5035	Methoxyphenamine Hydrochloride	5588-10-3	-33.3518	-24.8428	-8.509	10.5313
T2225	4-Hydroxyantipyrine	1672-63-5	-33.2509	-32.5954	-0.6554	3.5456
T2O2701	L(+)-Ornithine hydrochloride	3184-13-2	-33.2466	-20.9898	-12.2568	1.3466
T1394	Ibuprofen	15687-27-1	-33.237	-27.7518	-5.4852	8.1425
T2191	Tacrine hydrochloride hydrate	206658-92-6	-33.232	-29.7396	-3.4923	0
T1462	Captopril	62571-86-2	-33.2247	-30.0002	-3.2246	7.2047
T0024	Primidone	125-33-7	-33.1921	-32.7613	-0.4308	9.9141
T3154	Citral	5392-40-5	-33.1783	-33.6466	0.4683	4.6873
T7386	Bretylium tosylate	61-75-6	-33.1678	-31.7269	-1.4409	15.1288
T1119	Rasagiline	136236-51-6	-33.156	-32.2719	-0.8841	2.235
T1252	PAMBA	56-91-7	-33.1453	-29.4801	-3.6652	3.6201
T5S0636	Citric acid	77-92-9	-33.1291	-33.3734	0.2443	12.3162
T0514	Phenylindione	83-12-5	-33.1224	-33.3667	0.2443	6.3172
T7974	Lynestrenol	52-76-6	-33.1211	-33.0626	-0.0584	3.3182
T7049	Herniarin	531-59-9	-33.1185	-32.8928	-0.2256	0.3912
T1247	Clonidine hydrochloride	4205-91-8	-33.1088	-33.8134	0.7046	4.1046
T0397	Tetryzoline	84-22-0	-33.1046	-32.9645	-0.1401	2.4389
T1680	Tolazoline hydrochloride	59-97-2	-33.0783	-32.0807	-0.9976	0.9844
T1578	Pargyline hydrochloride	306-07-0	-33.0641	-30.4893	-2.5749	2.773
T6962	Rasagiline Mesylate	161735-79-1	-33.0496	-32.1887	-0.8609	2.167
T6522	Guanabenz Acetate	23256-50-0	-33.0383	-31.1189	-1.9194	2.5278
T1099	Formestane	566-48-3	-33.0014	-32.8936	-0.1077	0.7956
T6703	Tetrahydrozoline HCl	522-48-5	-32.9781	-30.4734	-2.5047	7.93
T9115	Vanillic Acid	121-34-6	-32.972	-25.1724	-7.7996	3.5809
T1230	Pamabrom	606-04-2	-32.9707	-32.9701	-0.0005	0
T1083	Theophylline	58-55-9	-32.9641	-32.5587	-0.4054	0
T1644	Dopamine hydrochloride	62-31-7	-32.9429	-21.711	-11.2319	1.9038
T6819	Desogestrel	54024-22-5	-32.9209	-33.1423	0.2213	11.1779
T4434	Tirapazamine	27314-97-2	-32.9135	-32.7329	-0.1806	0.0619
T0731	N,N-Diethyl-m-toluamide	134-62-3	-32.9125	-33.6171	0.7046	8.3488
T0928	L(+)-Ascorbic acid	50-81-7	-32.9088	-26.5162	-6.3926	0.5701
T1657	Varenicline Tartrate	375815-87-5	-32.9074	-31.2132	-1.6943	0

T0743	DL-Carnitine	461-06-3	-32.9053	-29.9119	-2.9934	5.8624
T6674	Sodium ascorbate	134-03-2	-32.9022	-28.7468	-4.1555	1.7054
T4246	Varenicline	866823-63-4	-32.8859	-31.3897	-1.4962	0
T1429	Hydroxyprogesterone	68-96-2	-32.8336	-32.5136	-0.3199	9.1585
T8031	Terephthalic acid	100-21-0	-32.8292	-21.9931	-10.8361	0.8215
T0349	Riluzole	1744-22-5	-32.8225	-32.5192	-0.3033	1.64
T0759	Monobenzone	103-16-2	-32.7887	-31.0663	-1.7224	2.5749
T0002	Ethamsylate	2624-44-4	-32.7763	-30.8678	-1.9085	2.904
T1711	Harmine	442-51-3	-32.7614	-31.7034	-1.058	4.2132
T0669	Chloramine-T	127-65-1	-32.7591	-32.628	-0.1311	1.8955
T6765	Atipamezole hydrochloride	104075-48-1	-32.7477	-32.3004	-0.4473	5.8523
T2140	Parthenolide	20554-84-1	-32.7306	-32.9674	0.2368	0
T4277	Osilodrostat	928134-65-0	-32.7159	-32.3173	-0.3986	22.8934
T0877	Gallic acid	149-91-7	-32.6762	-28.8371	-3.8391	0.5298
T1210	Sodium etidronate	7414-83-7	-32.6599	-27.2303	-5.4296	1.3258
T5665	Geraniol	106-24-1	-32.6482	-32.4243	-0.2239	4.4163
T1631	Sulbactam	68373-14-8	-32.6304	-25.7599	-6.8705	4.6312
T0846	L(-)-Carnitine	541-15-1	-32.6124	-29.2718	-3.3406	6.4039
T0192	Levetiracetam	102767-28-2	-32.555	-31.6947	-0.8604	5.208
T0734	Antipyrine	60-80-0	-32.5109	-32.1278	-0.3831	5.2542
T8056	Boldenone	846-48-0	-32.4855	-32.593	0.1075	0.7507
T0780	Mestinon	101-26-8	-32.4719	-30.3535	-2.1184	5.437
T0326L	L-Glutamine	56-85-9	-32.4592	-23.7175	-8.7417	3.9032
T6475	DL-Carnitine HCl	461-05-2	-32.4459	-28.491	-3.9549	3.2252
T0944	Levamisole hydrochloride	16595-80-5	-32.4387	-32.5709	0.1321	3.9499
T7146	Piperazine adipate	142-88-1	-32.3992	-20.9208	-11.4784	4.8256
T4557	Amylmetacresol	1300-94-3	-32.389	-32.685	0.296	7.0182
T0090	Moroxydine hydrochloride	3160-91-6	-32.3886	-27.6639	-4.7247	6.5759
T4558	Trioxsalen	3902-71-4	-32.3723	-31.6419	-0.7304	0
T1323	Artemether	71963-77-4	-32.3353	-30.6228	-1.7125	4.4011
T5603	Phthalic acid	88-99-3	-32.3301	-23.6424	-8.6877	3.4009
T0105	Carbachol	51-83-2	-32.3237	-30.5589	-1.7648	3.7131
T4514	N-Acetyl-D-Glucosamine	7512-17-6	-32.3112	-32.942	0.6308	4.5728
T5286	Glycyl-glycine	556-50-3	-32.3055	-22.5551	-9.7504	1.755
T0935	BHT FCC/NF	128-37-0	-32.2874	-32.5433	0.2559	26.0823
T0043	2-Amino-6-methylheptane	543-82-8	-32.2628	-16.6467	-15.6161	5.2932
T3608	Carbasalate?Calcium	5749-67-7	-32.2572	-31.232	-1.0252	3.894
T0058	2-Ethoxybenzamide	938-73-8	-32.2537	-32.0616	-0.1921	1.7236
T5939	Calcium dobesilate	20123-80-2	-32.2305	-30.6174	-1.6131	3.7142
T2863	p-Coumaric acid	501-98-4	-32.2147	-31.882	-0.3327	2.6876
T0469	Octopamine hydrochloride	770-05-8	-32.2037	-27.026	-5.1777	2.6479
T0887	D(+)-Glucose	50-99-7	-32.2031	-29.8097	-2.3934	0.5404
T4285	Crisaborole	906673-24-3	-32.1872	-32.1872	0	4.5245
T2754	Oxymatrine	16837-52-8	-32.1668	-31.8357	-0.3311	0
T0005	Aspirin	50-78-2	-32.1585	-31.0915	-1.067	4.2403
T0424	Bismuth subsalicylat	14882-18-9	-32.1491	-19.1113	-13.0379	0.6743
T3159	Guaiazulene	489-84-9	-32.1276	-31.78	-0.3476	2.346
T3268	Mildronate	76144-81-5	-32.1226	-26.771	-5.3517	2.2556

T0289	Aminacrine	90-45-9	-32.1037	-29.5508	-2.5529	0
T0513	Iodoquinol	83-73-8	-32.0913	-31.5601	-0.5312	0.0844
T0857	Fenipentol	583-03-9	-32.0801	-31.5133	-0.5668	3.28
T0045	Heptaminol hydrochloride	543-15-7	-32.0238	-27.0085	-5.0153	7.7649
T3126	Bethanechol chloride	590-63-6	-31.9811	-30.0069	-1.9742	7.2155
T0521	Thimerosal	54-64-8	-31.9634	-29.7884	-2.175	2.3563
T0702	Gabapentin	60142-96-3	-31.9601	-24.4159	-7.5442	13.329
T1221	Acetylcholine chloride	60-31-1	-31.9438	-30.1959	-1.748	5.7733
T8047	(1R)-(-)-Menthyl acetate	2623-23-6	-31.9017	-31.6125	-0.2891	7.0832
T2386	Pirfenidone	53179-13-8	-31.8995	-31.8664	-0.0332	4.6075
T0834	Ethylparaben	120-47-8	-31.8894	-31.3329	-0.5564	2.8028
T2798	Esculetin	305-01-1	-31.8799	-31.5695	-0.3104	0.0596
T0607	Dihydroartemisinin	71939-50-9	-31.8748	-28.5902	-3.2846	1.2731
T6519	Gramine	87-52-5	-31.8729	-28.3766	-3.4963	2.5573
T2228	Adipic acid	124-04-9	-31.842	-23.774	-8.068	4.1314
T2797	Harmaline	304-21-2	-31.8284	-31.9981	0.1697	18.0885
T2851	Daphnetin	486-35-1	-31.8072	-29.7674	-2.0398	0.0811
T6451	Clodronate Disodium	22560-50-5	-31.7666	-27.6331	-4.1335	0.1338
T4812	D-Mannose	3458-28-4	-31.7145	-30.033	-1.6816	1.2027
T0881	6-Aminocaproic acid	60-32-2	-31.7029	-18.7507	-12.9522	2.8849
T0436	Acexamic Acid	57-08-9	-31.6977	-26.3347	-5.3631	3.7294
T1297	Eugenol	97-53-0	-31.6232	-30.6978	-0.9254	1.9953
T1391	4-Methylumbelliferone	90-33-5	-31.6151	-31.7229	0.1078	0.027
T0591	Alpha-D-galactose	59-23-4	-31.6088	-30.6834	-0.9254	1.5994
T2875	Isopsoralen	523-50-2	-31.6046	-31.9145	0.3099	0
T0551	D-Ornithine monohydrochloride	16682-12-5	-31.571	-19.5013	-12.0697	2.3187
T0359	2-Aminoheptane	123-82-0	-31.5647	-17.7518	-13.813	4.1112
T5552	Methyl cinnamate	103-26-4	-31.5632	-31.13	-0.4332	10.715
T3269	Metadoxine	74536-44-0	-31.5486	-29.0735	-2.4751	3.6876
T0326	DL-Glutamine	6899-04-3	-31.5269	-21.8507	-9.6762	3.4342
T1161	Protionamide	14222-60-7	-31.5036	-30.9817	-0.522	3.9776
T2942	Psoralen	66-97-7	-31.4973	-31.9223	0.425	0
T0908	Chloquinan	72-80-0	-31.489	-30.9089	-0.5801	0.0713
T0933	Delta-Gluconolactone	90-80-2	-31.4748	-29.7862	-1.6886	1.0907
T0395	Pyrantel tartrate	33401-94-4	-31.4684	-31.1544	-0.314	2.652
T1693	7-Hydroxycoumarin	93-35-6	-31.4591	-31.4564	-0.0027	0.0284
T5855	Edrophonium chloride	116-38-1	-31.4331	-27.9612	-3.4719	10.7868
T0924	Benzocaine	94-09-7	-31.4261	-31.5097	0.0836	1.5065
T0494	Tetramisole hydrochloride	5086-74-8	-31.4234	-31.0663	-0.3571	1.8237
T1378	Tegafur	17902-23-7	-31.4218	-31.2889	-0.1329	8.4725
T0973	Pyridoxine	65-23-6	-31.4064	-29.3462	-2.0602	3.7104
T0973L	Pyridoxine hydrochloride	58-56-0	-31.3816	-28.899	-2.4827	3.7156
T4786	D-(-)-Ribose	50-69-1	-31.3724	-29.5766	-1.7958	2.2825
T0845	Cyromazine	66215-27-8	-31.3552	-31.0902	-0.265	0.9102
T1613	Hydralazine hydrochloride	304-20-1	-31.3491	-30.693	-0.6561	0.6164
T0701	Broxyquinoline	521-74-4	-31.3353	-30.8555	-0.4799	0.0824
T1162	Acipimox	51037-30-0	-31.3347	-29.9845	-1.3502	0.1256
T6751	Acetylcholine iodide	2260-50-6	-31.3195	-29.7779	-1.5416	16.2062

T6782	Benzocaine hydrochloride	23239-88-5	-31.3061	-31.2518	-0.0543	1.5595
T0021	Vanatone	70-70-2	-31.2907	-30.097	-1.1936	2.2472
T0123	Sulfanilamide	63-74-1	-31.2826	-30.7864	-0.4962	3.2885
T1309	Propylthiouracil	51-52-5	-31.2614	-31.2029	-0.0585	1.4582
T5059	L(+)-Tartaric acid	87-69-4	-31.2533	-22.5649	-8.6884	6.0983
T2740	Cinnamic acid	140-10-3	-31.2121	-26.9449	-4.2672	1.7339
T0925	Allantoin	97-59-6	-31.1967	-29.5729	-1.6238	0.9031
T0756	Ethoxyquin	91-53-2	-31.1593	-31.8618	0.7025	3.1003
T5984	Indole-3-carboxylic acid	771-50-6	-31.1506	-30.7075	-0.443	0.4481
T4844	L-Methionine	63-68-3	-31.1364	-22.2275	-8.909	4.0129
T0938	Thiabendazole	148-79-8	-31.1313	-30.3019	-0.8294	0.4317
T4502	BISMUTH SUBGALLATE	99-26-3	-31.1306	-29.0311	-2.0995	1.9374
T0407	Edaravone	89-25-8	-31.0852	-30.9538	-0.1313	1.037
T1682	Methacholine Chloride	62-51-1	-31.0455	-28.4172	-2.6283	6.3988
T2198	Arecoline HBr	300-08-3	-31.0113	-30.222	-0.7893	1.2346
T2552	Succimer	304-55-2	-31.0109	-22.519	-8.4919	2.2329
T0680	Lamotrigine	84057-84-1	-30.9917	-28.8454	-2.1463	1.9317
T3280	Disodium clodronate tetrahydrate	88416-50-6	-30.9914	-26.7605	-4.2309	0.1896
T8138	Phenazine methosulfate	299-11-6	-30.9902	-30.4004	-0.5897	0
T5591	Dihydrotestosterone(DHT)	521-18-6	-30.9669	-31.1545	0.1876	1.0026
T0625	Theobromine	83-67-0	-30.9238	-30.9319	0.0081	0
T0392	Artemisinin	63968-64-9	-30.8328	-32.1528	1.32	0
T0876	Clioquinol	130-26-7	-30.8259	-30.2062	-0.6197	0.0818
T0874	Dimetridazole	551-92-8	-30.8153	-29.9225	-0.8928	2.6096
T5029	Oxaceprol	33996-33-7	-30.8093	-27.5442	-3.2651	4.3132
T4195	Betahistine	5638-76-6	-30.7882	-27.2764	-3.5117	0.3504
T5034	Nicotine Ditartrate	65-31-6	-30.7706	-29.6934	-1.0773	3.5998
T0246	Betahistine dihydrochloride	5579-84-0	-30.7681	-27.2993	-3.4688	0.5127
T1387	Acetylleucine	99-15-0	-30.7607	-26.5044	-4.2563	7.5515
T0246L	Betahistine mesylate	54856-23-4	-30.6923	-26.9524	-3.7398	0.4997
T2O2731	L-Aspartic acid	56-84-8	-30.6867	-20.8473	-9.8394	1.5947
T1417	Mechlorethamine hydrochloride	55-86-7	-30.6649	-28.1373	-2.5276	2.2429
T0778	Phenacetin	62-44-2	-30.5941	-29.3244	-1.2697	2.6939
T0032	DL-Xylose	25990-60-7	-30.585	-30.0065	-0.5785	1.3892
T1662	5-Aminolevulinic acid hydrochloride	5451-09-2	-30.5627	-21.4542	-9.1085	2.4404
T0355	Valpramide	2430-27-5	-30.557	-30.4369	-0.1201	3.8946
T0235	Modaline sulfate	2856-75-9	-30.5504	-30.7604	0.21	2.6254
T0983L	DL-Penicillamine	52-66-4	-30.5452	-18.7027	-11.8425	5.0088
T0589	Xylitol	87-99-0	-30.5406	-29.4636	-1.077	1.8192
T2187	2-Aminobenzenesulfonamide	3306-62-5	-30.462	-29.9063	-0.5558	3.2042
T0562	Protocatechuic acid	99-50-3	-30.4419	-27.5507	-2.8912	0.4179
T6742	2-Deoxy-D-glucose	154-17-6	-30.4326	-27.9669	-2.4657	0.647
T0515	Tioxolone	4991-65-5	-30.4006	-29.3466	-1.054	0.0303
T0952	Carbimazole	22232-54-8	-30.3837	-29.6125	-0.7712	7.4998
T0889	Saccharin	81-07-2	-30.367	-32.5766	2.2096	0
T0308	Etidronate	2809-21-4	-30.3632	-22.891	-7.4722	1.2751
T1500	Xylazine hydrochloride	23076-35-9	-30.3014	-30.3215	0.0202	2.8892
T1300	Propofol	2078-54-8	-30.2918	-30.1015	-0.1902	12.692

T0227	Bromisoval	496-67-3	-30.2399	-30.5096	0.2697	8.5233
T1128	Oxiracetam	62613-82-5	-30.2385	-29.3532	-0.8853	2.3202
T7465	4-Methylbenzylidene camphor	36861-47-9	-30.2248	-29.0773	-1.1475	3.5454
T2S2264	Linalool	78-70-6	-30.2181	-30.321	0.1029	5.4548
T2S0493	Isoeugenol	97-54-1	-30.2046	-29.4025	-0.8021	7.0524
TJO2742	methionine	59-51-8	-30.1912	-25.1195	-5.0717	1.5862
T0500	Capitrol	773-76-2	-30.1885	-29.9373	-0.2511	0.0806
T8025	Phenylglyoxylic acid	611-73-4	-30.1722	-29.2142	-0.9581	1.7784
T0598	Iminostilbene	256-96-2	-30.1552	-29.5825	-0.5727	0
T6717	Vanillin	121-33-5	-30.1424	-28.525	-1.6174	0.6622
T0065	Acetaminophen	103-90-2	-30.121	-30.0046	-0.1164	0.8461
T0883	Homotaurine	3687-18-1	-30.079	-18.0095	-12.0694	0.9875
T1658	Selenomethionine	1464-42-2	-30.0572	-28.3596	-1.6977	2.4863
T0665	2,6-Dihydroxypurine	69-89-6	-30.0509	-27.8995	-2.1514	0
T0438	Proflavine Hemisulfate	1811-28-5	-30.0493	-29.264	-0.7853	0
T0875	Acetylcysteine	616-91-1	-30.0365	-25.6529	-4.3837	1.1245
T0646	Mesalamine	89-57-6	-30.0178	-27.4009	-2.617	1.118
T8061	Citronellal	106-23-0	-30.0156	-29.6587	-0.357	7.2299
T5889	Kojic acid	501-30-4	-29.9719	-28.1626	-1.8093	1.8923
T8526	Metformin	657-24-9	-29.9446	-25.231	-4.7136	1.8699
T3719	Daminozide	1596-84-5	-29.9204	-23.9905	-5.9299	0.9564
T2S0122	Mandelic acid	611-71-2	-29.8902	-26.166	-3.7242	1.0894
T3946	Caprylic acid	124-07-2	-29.8676	-25.1697	-4.6979	5.7788
T1165	Ethinamide	536-33-4	-29.8143	-29.4236	-0.3907	2.5459
T2927	Trigonelline hydrochloride	6138-41-6	-29.8119	-26.8768	-2.9352	0.3546
T7449	1, 10-Phenanthroline monohydrate	5144-89-8	-29.7675	-29.9355	0.1679	0
T1044	Clopidol	2971-90-6	-29.736	-29.1964	-0.5396	0
T0961	Trometamol	77-86-1	-29.7324	-10.4151	-19.3173	3.2315
T2833	Dehydrocostus Lactone	477-43-0	-29.7251	-30.5235	0.7984	0
T1650	Chlorzoxazone	95-25-0	-29.6539	-29.4587	-0.1952	0
T7910	Anisic aldehyde	123-11-5	-29.6481	-28.9226	-0.7255	1.8927
T2969	Lawsone	83-72-7	-29.6272	-26.7955	-2.8317	0
T0307	Tyloxapol	25301-02-4	-29.5877	-27.1413	-2.4464	0.9711
T0762	Trans-Anethole	4180-23-8	-29.5772	-28.9053	-0.672	5.5527
T3095	Tyramine	51-67-2	-29.5675	-25.1988	-4.3686	1.7191
T0746	Orotic acid	65-86-1	-29.5426	-26.8009	-2.7417	0.2256
T8599	Betazole hydrochloride	138-92-1	-29.5262	-21.6774	-7.8488	0.2431
T0441	Pasiniazid	2066-89-9	-29.4696	-27.3628	-2.1068	1.1248
T1250	Triethylenethiophosphoramide	52-24-4	-29.4496	-29.1853	-0.2644	6.5598
T2O2774	L-Asparagine	70-47-3	-29.4283	-22.0706	-7.3578	1.2631
T0965	Histamine	51-45-6	-29.4228	-20.9936	-8.4292	0.1403
T0014	cis-Anethol	104-46-1	-29.4092	-28.7478	-0.6614	3.381
T5983	o-Phenanthroline	66-71-7	-29.4037	-29.8404	0.4367	0
T0399	Methyl salicylate	119-36-8	-29.3888	-26.8688	-2.52	10.8986
T2954	Fructose	7660-25-5	-29.3826	-27.3049	-2.0778	2.0102
T3728	Methylparaben	99-76-3	-29.3791	-29.2368	-0.1423	0.8029
T0020	Sodium 4-aminosalicylate dihydrate	6018-19-5	-29.3632	-26.5943	-2.7689	1.3769
T4723	D-(-)-Tagatose	87-81-0	-29.3364	-27.3247	-2.0117	3.4417

T2928	L-Rhamnose monohydrate	6155-35-7	-29.3242	-27.5609	-1.7632	1.6538
T8392	L(+)-Asparagine monohydrate	5794-13-8	-29.316	-21.8128	-7.5032	1.8849
T1296	Aminopar	65-49-6	-29.2847	-26.5671	-2.7176	6.8645
T1602	Valproic acid sodium salt	1069-66-5	-29.2788	-24.4648	-4.814	7.6212
T2902	Costunolide	553-21-9	-29.2348	-29.7574	0.5227	0
T6474	Divalproex Sodium	76584-70-8	-29.2281	-24.4534	-4.7747	3.3662
T6535	Histamine Phosphate	51-74-1	-29.2242	-20.6164	-8.6078	0.2373
T2724	Caryophyllene oxide	1139-30-6	-29.2165	-28.8345	-0.382	0
T1630	Tiopronin	1953-02-2	-29.1877	-23.2664	-5.9213	3.1866
T6833	Favipiravir	259793-96-9	-29.1312	-27.4761	-1.6551	0.6943
T2390	Cevimeline hydrochloride hemihydrate	153504-70-2	-29.1169	-26.5487	-2.5682	0
T7459	Abametapir	1762-34-1	-29.0719	-28.6241	-0.4478	2.4715
T1598	Rimantadine hydrochloride	1501-84-4	-29.0543	-25.6521	-3.4022	9.1592
T0426	Piracetam	7491-74-9	-29.028	-28.2174	-0.8105	1.7524
T1111	Pralidoxime Chloride	51-15-0	-29.0114	-26.2347	-2.7766	2.5247
T6805	Choline bitartrate	87-67-2	-28.9831	-22.2462	-6.7369	1.1595
T6781	Benzenesulfonamide	98-10-2	-28.9802	-28.3345	-0.6457	1.2843
T2916	Patchouli alcohol	5986-55-0	-28.9316	-27.5779	-1.3537	8.5226
T1025	Tranylcypromine (2-PCPA) hydrochloride	1986-47-6	-28.8852	-15.478	-13.4073	0.7651
T0434	Dicurone	32449-92-6	-28.8769	-27.7556	-1.1213	1.838
T3S0717	Thymol	89-83-8	-28.8462	-27.8139	-1.0323	3.6104
T7064	Valproic Acid	99-66-1	-28.797	-24.0179	-4.7791	8.6836
T0449	Menadione	58-27-5	-28.7961	-28.9034	0.1074	0
T8127	Disodium Phosphate	7558-79-4	-28.7849	-1.1115	-27.6734	0
T0899	Dehydroacetic acid	520-45-6	-28.7621	-27.9491	-0.813	2.3083
T0728	Ethosuximide	77-67-8	-28.7354	-28.4968	-0.2386	7.3867
T0972	Isoniazid	54-85-3	-28.7162	-27.5365	-1.1797	1.0651
T2989	4-Hydroxybenzoic acid	99-96-7	-28.7045	-21.6451	-7.0594	1.8688
T4883	Creatine	57-00-1	-28.6468	-22.96	-5.6868	1.7546
T0775	Coumarin	91-64-5	-28.6277	-28.814	0.1863	0
T2945	Germacrone	6902-91-6	-28.5871	-28.9206	0.3335	9.4854
T2861	Tyrosol	501-94-0	-28.5525	-26.9091	-1.6435	0.8229
T0022	Taurine	107-35-7	-28.4963	-10.7162	-17.7801	0.2104
T0063	isoleucine	73-32-5	-28.4347	-21.8156	-6.6191	5.4634
T2S0850	Malic acid	6915-15-7	-28.4221	-17.8478	-10.5744	4.71
T5299	4-Nitrophenol	100-02-7	-28.3906	-27.6301	-0.7605	1.1221
T3114	Povidone-iodine	25655-41-8	-28.3863	-28.5563	0.17	4.1211
T0403	Antepan	110-85-0	-28.2929	-7.9663	-20.3266	0
T6598	MPEP	96206-92-7	-28.2916	-27.9021	-0.3895	0
T0067	Nicotinic acid N-oxide	2398-81-4	-28.2757	-23.0205	-5.2552	0.238
T2979	DL-Menthol	89-78-1	-28.2329	-28.0135	-0.2195	5.02
T6534	Histamine 2HCl	56-92-8	-28.2316	-21.2084	-7.0232	0.1801
T0740	Metformin hydrochloride	1115-70-4	-28.222	-25.581	-2.641	2.9047
T4S1990	Carvacrol	499-75-2	-28.2218	-28.4846	0.2628	2.2269
T8080	2-Methylhexanoic acid	4536-23-6	-28.2099	-23.4354	-4.7745	6.4321
T5562	Methyl nicotinate	93-60-7	-28.2077	-28.018	-0.1897	1.227
T2201	6-Mercaptopurine monohydrate	6112-76-1	-28.1617	-26.5503	-1.6113	0
T2719	Succinic acid	110-15-6	-28.1125	-18.6133	-9.4992	2.2905

T0983	d-penicillamin	52-67-5	-28.0956	-25.2511	-2.8445	4.7595
T0910	Terpin Monohydrate	2451-01-6	-28.0697	-27.7175	-0.3522	7.1702
T1058	Carboplatin	41575-94-4	-28.0588	-19.5843	-8.4745	3.495
T5723	DL-Menthol	1490-04-6	-28.0578	-27.8586	-0.1992	5.0266
T2A2476	L-Hydroxyprolin	51-35-4	-28.0362	-23.0524	-4.9838	1.1262
T7060	1-Adamantylamine	768-94-5	-28.0289	-15.0863	-12.9425	0
T0004	Salicylamide	65-45-2	-27.9977	-26.7907	-1.207	2.6447
T0509	Dermofongin	130-16-5	-27.9714	-26.7678	-1.2037	0.0673
T0010	Mercaptopurine	50-44-2	-27.9439	-26.4288	-1.5151	0
T2953	Ligustrazine hydrochloride	76494-51-4	-27.926	-28.0297	0.1037	0
T1406	Amantadine hydrochloride	665-66-7	-27.908	-15.2061	-12.7019	0
T3S1553	Cinnamic aldehyde	14371-10-9	-27.8642	-26.9631	-0.9011	1.1487
T8048	Diphenylamine Hydrochloride	537-67-7	-27.8522	-27.4028	-0.4494	2.994
T6862	Isatin	91-56-5	-27.8442	-27.6818	-0.1624	0
T0880	Bronopol	52-51-7	-27.8223	-26.6348	-1.1875	0.9849
T1082	Benzyl isothiocyanate	622-78-6	-27.7623	-27.5051	-0.2573	1.1841
TJO2691	L-(-)-Fucose	2438-80-4	-27.7434	-26.8805	-0.8629	2.2804
T7468	??-Terpineol	10482-56-1	-27.7278	-26.7831	-0.9447	6.0683
T0257	Mepiroxol	6968-72-5	-27.6744	-24.443	-3.2314	0.3332
T2943	5-Hydroxymethyl-2-furaldehyde	67-47-0	-27.6633	-26.9888	-0.6745	0.1843
T3245	(S)-(+)-Carvone	2244-16-8	-27.663	-26.8535	-0.8094	2.5676
T6551	Isosorbide	652-67-5	-27.6591	-26.3517	-1.3075	0.0371
T0508	4-Aminobutyric acid	56-12-2	-27.6428	-18.8858	-8.7569	0.6252
T0649	Salicylic acid	69-72-7	-27.5101	-21.0509	-6.4593	1.2832
T0987	Gimeracil	103766-25-2	-27.4994	-24.7834	-2.716	0
T0587	L(+)-Arabinose	87-72-9	-27.4989	-26.4259	-1.0731	0.3718
T9114	??-Caryophyllene	87-44-5	-27.4942	-27.4015	-0.0927	0
T0406	Acetanilide	103-84-4	-27.4689	-27.7052	0.2363	1.7075
T0421	Inositol	87-89-8	-27.4382	-26.3787	-1.0594	3.6198
T8073	Sodium succinate	150-90-3	-27.4102	-19.0912	-8.3191	1.789
T0940	Acesulfame Potassium	55589-62-3	-27.3513	-28.4616	1.1103	0
T4825	D(+)-Xylose	58-86-6	-27.3282	-25.8422	-1.486	0.1251
T0564	1-Indanone	83-33-0	-27.22	-27.0984	-0.1216	0
T1565	Deferidone	30652-11-0	-27.2149	-26.2906	-0.9243	0.6323
T3O2749	L-Leucine	61-90-5	-27.1737	-21.2149	-5.9588	3.0977
T1362	6-Hydroxypurine	68-94-0	-27.1721	-25.9255	-1.2467	0
T8362	Benzamidine HCl	1670-14-0	-27.1375	-22.9003	-4.2372	0.2283
T0316	Citiolone	17896-21-8	-27.1043	-26.1018	-1.0025	1.759
T0335	Sodium salicylate	54-21-7	-27.0929	-17.9585	-9.1344	1.7638
T8361	Tetraethylammonium bromide	71-91-0	-27.0439	-26.0476	-0.9963	9.3527
T1311	4-aminobenzoic acid	150-13-0	-27.0162	-25.114	-1.9022	0.3325
T8068	o-Toluic acid	118-90-1	-27.0063	-24.448	-2.5584	0.9642
T0221	Thioproline	444-27-9	-26.9724	-21.8515	-5.1208	0.5844
TN2264	Terpinine-4-ol	562-74-3	-26.9647	-26.8411	-0.1236	7.521
T1443	Memantine hydrochloride	41100-52-1	-26.9181	-16.6713	-10.2468	0
T0692	Allopurinol	315-30-0	-26.8801	-25.6725	-1.2077	0.0933
T4913	Maleic acid	110-16-7	-26.7836	-14.4249	-12.3588	1.7066
T2O2683	proline	147-85-3	-26.777	-21.9152	-4.8618	0.5651

T2O2776	L-Valine	72-18-4	-26.6821	-22.5525	-4.1296	3.9597
T0064	Adenine	73-24-5	-26.6523	-26.1588	-0.4935	0
T1396	Bemegrid	64-65-3	-26.574	-26.8031	0.2291	6.5496
TWO2759	Thymine	65-71-4	-26.5656	-26.2325	-0.3331	0
T6369	Adenine sulfate	321-30-2	-26.5458	-26.1072	-0.4386	0
T5543	TriacetonaMine	826-36-8	-26.5454	-24.5156	-2.0297	0
T0583	Erythritol	149-32-6	-26.5379	-24.4803	-2.0575	0.7873
T0492	Dimethyl fumarate	624-49-7	-26.519	-25.6316	-0.8874	7.1264
T2P2978	P-Cymene	99-87-6	-26.5184	-26.715	0.1966	2.4783
T3763	Fumaric acid	110-17-8	-26.4853	-15.1699	-11.3154	1.124
T2O2777	threonine	72-19-5	-26.3199	-20.908	-5.4119	5.8124
T8026	(S)-(?)-Limonene	5989-54-8	-26.2413	-26.0103	-0.2311	5.7558
T1426	Pyrazinamide	98-96-4	-26.1743	-25.5309	-0.6434	0.1267
T0671	2-Naphthol	135-19-3	-26.1639	-25.0484	-1.1155	0.0323
T4575	Chromium picolinate	14639-25-9	-26.1456	-24.4803	-1.6653	0.9885
T0404	Hydroxyquinoline	148-24-3	-26.1221	-25.127	-0.9951	0.0724
T0220	Foscarnet sodium	63585-09-1	-26.0606	-18.1603	-7.9003	0
T2O2721	cysteine	52-90-4	-25.9158	-19.1037	-6.8121	1.5178
T0001	Ferrous fumarate	141-01-5	-25.8978	-18.0189	-7.8789	0.4319
T1589	D-Cycloserine	68-41-7	-25.8316	-10.3138	-15.5178	0
T0898	Chloroxylenol	88-04-0	-25.8168	-25.2298	-0.587	0.0369
T0934	Nicotinamide	98-92-0	-25.7805	-25.206	-0.5745	0.1755
T0304	Methylthiouracil	56-04-2	-25.6747	-25.5465	-0.1282	0
T5545	Maltol	118-71-8	-25.6632	-24.6058	-1.0574	0.4173
T0283	Trimethadione	127-48-0	-25.5667	-25.1168	-0.45	0
T7446	Acetophenone	98-86-2	-25.5623	-25.2126	-0.3497	2.5364
T2O2730	serine	56-45-1	-25.5464	-19.0445	-6.5019	0.7032
TCS0102	Pulegone	89-82-7	-25.5363	-25.7805	0.2442	6.6817
T3088	N-Ethylmaleimide	128-53-0	-25.5084	-25.1845	-0.3239	0.9182
T1246	Bornyl acetate	76-49-3	-25.4627	-24.3248	-1.1379	5.7043
T0011	Phenethyl alcohol	60-12-8	-25.4612	-23.8041	-1.6572	0.7976
T0501	4-Methoxyphenol	150-76-5	-25.4444	-24.2505	-1.1939	1.3858
T3243	Betaine	107-43-7	-25.4122	-22.8387	-2.5736	5.5547
T1407	L-Menthol	2216-51-5	-25.3469	-24.9722	-0.3747	4.7468
T8019	Sorbic acid	110-44-1	-25.3046	-23.7041	-1.6004	4.0807
T8734	Choline	123-41-1	-25.2293	-22.8505	-2.3789	1.4135
T5551	Levulinic acid	123-76-2	-25.2276	-20.6102	-4.6174	2.9101
T1007	Lupetidin	504-03-0	-25.1451	-23.1305	-2.0146	0
T8141	L-Cysteine HCl	52-89-1	-25.1414	-17.0702	-8.0712	1.2642
T1039	Choline chloride	67-48-1	-25.1294	-22.8149	-2.3145	1.3081
T3349	Tavaborole	174671-46-6	-25.0872	-25.0872	0	0
T0763	Diolane	107-41-5	-25.0544	-23.1494	-1.905	4.0921
T0879	Nicotinic acid	59-67-6	-25.0478	-23.1168	-1.931	0.2477
T0019	Betaine hydrochloride	590-46-5	-24.9708	-23.7136	-1.2572	6.6159
T5549	Melamine	108-78-1	-24.9185	-24.6676	-0.2509	0
T0833	Benzoic acid	65-85-0	-24.854	-22.6113	-2.2428	0.2291
T0496	Creatinine	60-27-5	-24.8505	-22.7315	-2.119	0.2382
T3748	orcinol	504-15-4	-24.8105	-23.8147	-0.9958	0.0673

T0311	2-Hydroxybenzyl alcohol	90-01-7	-24.7493	-24.0043	-0.745	1.3531
T8124	Sodium benzoate	532-32-1	-24.7349	-22.3193	-2.4157	0.3235
T0710	L-Cycloserine	339-72-0	-24.6687	-10.9663	-13.7025	0
T1234	5,5-Dimethyloxazolidine-2,4-dione	695-53-4	-24.4511	-20.9726	-3.4785	0
T3267	Potassium guaiacolsulfonate	78247-49-1	-24.3906	-23.9448	-0.4458	1.4994
T0774	Aptal	59-50-7	-24.3233	-24.0265	-0.2968	0.0331
T1414	Mesna	19767-45-4	-24.3065	-18.2495	-6.057	2.7283
T0418	Allylthiourea	109-57-9	-24.2649	-24.2771	0.0122	2.067
T4746	Purine	120-73-0	-24.2254	-23.8004	-0.425	0
T1310	Deracil	141-90-2	-24.2093	-23.7144	-0.4949	0
T1365	Thioisonicotinamide	2196-13-6	-24.2042	-23.6421	-0.5621	0.5415
T8363	DL-Serine	302-84-1	-24.1836	-15.7558	-8.4278	1.6502
T8394	(R)-Serine	312-84-5	-24.1836	-15.7558	-8.4278	1.6502
T1301	Guaiacol	90-05-1	-24.1331	-22.6366	-1.4965	0.8883
T3749	Pyrogallol	87-66-1	-24.072	-22.8398	-1.2322	0.0871
T0627	beta-Alanine	107-95-9	-24.0315	-16.9241	-7.1074	0.3633
T0986	Fluorocytosine	2022-85-7	-24.0021	-23.8761	-0.126	0.0572
T4497	Amifampridine	54-96-6	-23.9647	-11.3363	-12.6284	0
T0984	Fluorouracil	51-21-8	-23.8284	-22.8615	-0.9669	0
T0901	Chlorobutanol	57-15-8	-23.5275	-22.9936	-0.534	4.0078
T5548	(-)-Verbenone	1196-01-6	-23.4649	-23.7354	0.2705	0
T5617	Furfural	98-01-1	-23.3908	-22.6841	-0.7067	0.0614
T1299	Roniacol	100-55-0	-23.3101	-22.5752	-0.7348	0.2439
T0069	Uracil	66-22-8	-23.2918	-23.1623	-0.1295	0
T1714	Eucalyptol	470-82-6	-23.2773	-22.3539	-0.9234	0
T4832	Cytosine	71-30-7	-23.2015	-23.0144	-0.1871	0.0595
T0296	Pempidine	79-55-0	-23.0904	-21.8497	-1.2406	0
T5882	3-Bromopyruvic acid	1113-59-3	-23.0038	-21.9399	-1.0639	1.3659
T6460	Cysteamine HCl	156-57-0	-22.9245	-7.0345	-15.89	0.0021
T4799	Indole	120-72-9	-22.8905	-22.5936	-0.2969	0
T0704	Cyanoacetohydrazide	140-87-4	-22.8642	-20.296	-2.5682	0.1103
TJO2729	alanine	56-41-7	-22.843	-14.9863	-7.8567	0.8827
T0781	Dithiopropanol	59-52-9	-22.8406	-22.3981	-0.4425	1.5006
T4989	Fosfomycin Tromethamine	78964-85-9	-22.83	-18.2555	-4.5745	3.0164
T1588	Kemantane	20098-14-0	-22.8109	-21.6765	-1.1344	0.0829
T0732	Benzyl alcohol	100-51-6	-22.435	-21.4831	-0.952	0.2851
T5304	p-Cresol	106-44-5	-22.3985	-21.9934	-0.4051	0.0283
T0041	Cresol	1319-77-3	-22.3448	-21.9235	-0.4212	0.0283
T5610	Anisole	100-66-3	-22.294	-21.3191	-0.975	11.8529
T4505	Ferrous Bisglycinate	20150-34-9	-22.2739	-15.6026	-6.6714	0.1906
T4512	Magnesium glycinate	14783-68-7	-22.2739	-15.6026	-6.6714	0.1906
T5604	4-Methyl-2-pentanone	108-10-1	-22.2195	-22.1792	-0.0403	9.4526
T4847	Pyrocatechol	120-80-9	-22.1295	-21.2707	-0.8588	0.0574
T0673	Hydroquinone	123-31-9	-22.0427	-21.0203	-1.0224	0.0593
T0745	Disodium monofluorophosphate	10163-15-2	-21.9798	-8.4217	-13.558	0
T0931	Resorcine	108-46-3	-21.9473	-21.1374	-0.8099	0.0596
T1080	2-Aminoethanethiol	60-23-1	-21.914	-6.6949	-15.2191	0.0008
T0648	4-Aminophenol	123-30-8	-21.8888	-21.0827	-0.8061	0.028

TMS1986	Camphene	79-92-5	-21.8719	-21.9236	0.0517	0
T0840	Methimazole	60-56-0	-21.7747	-21.6548	-0.1199	0
T3741	(-)-borneol	464-45-9	-21.7493	-21.2795	-0.4698	5.4651
T2O2680	Oxalic acid	144-62-7	-21.6531	-13.3259	-8.3272	0
T1393	Sodium butanoate	156-54-7	-21.525	-16.4292	-5.0957	0.4943
T2410	Nedaplatin	95734-82-0	-21.5177	-12.1237	-9.394	0
T3604	Sodium?Dichloroacetate	2156-56-1	-21.3692	-15.9124	-5.4568	0
T5577	p-Benzoquinone	106-51-4	-21.3212	-20.7881	-0.5331	0
T4776	Glycerol	56-81-5	-21.1537	-19.0226	-2.1311	0.4704
T3038	Borneol	507-70-0	-21.0681	-21.1969	0.1288	1.4683
T0882	D-Camphor	464-49-3	-20.9871	-20.8348	-0.1523	0
T2952	Camphor	76-22-2	-20.7619	-20.2599	-0.502	0
T8360	(-)-??-Pinene	18172-67-3	-20.6962	-20.6967	0.0005	0
T2O2728	glycine	56-40-6	-20.6234	-12.7618	-7.8616	0.5377
T4103	(L)-Sodium lactate	867-56-1	-20.5442	-14.9652	-5.579	0.6216
T4845	L-Lactic acid	79-33-4	-20.5442	-14.9652	-5.579	0.6216
T0051	Urethane	51-79-6	-20.5209	-19.9964	-0.5244	2.7521
T0358	Aminoguanidine hydrochloride	1937-19-5	-20.5046	-17.5359	-2.9687	0.0246
T4804	Pyruvic acid	127-17-3	-20.2553	-15.2545	-5.0008	0.1834
T5611	Pyrrolidine	123-75-1	-20.2515	-8.8531	-11.3985	0
T0495	Aminothiazole	96-50-4	-20.145	-18.9548	-1.1902	0
T0817	4-Aminopyridine	504-24-5	-20.0443	-19.7046	-0.3396	0
T0954	Anestan	151-67-7	-19.6268	-19.4885	-0.1382	0
T0765	4-Methylpyrazole	7554-65-6	-19.5509	-18.702	-0.8488	0
T5985	Glycolic acid	79-14-1	-18.6824	-13.0987	-5.5837	0
T5619	1,2-Propanediol	57-55-6	-18.5996	-18.2409	-0.3587	0.1067
T1592	Acetohydroxamic acid	546-88-3	-18.2065	-12.9279	-5.2785	0.0102
T0676	Hydroxyurea	127-07-1	-17.8876	-15.6357	-2.2519	0
T5202	Imidazole	288-32-4	-17.5217	-16.8581	-0.6636	0
T0415	Guanidine hydrochloride	50-01-1	-17.3269	-6.4577	-10.8692	0
T8131	Lithium carbonate	554-13-2	-16.574	-9.9502	-6.6237	0
T1564	Cisplatin	15663-27-1	-16.5013	-16.5013	0	0
T8129	Potassium acetate	127-08-2	-16.4332	-11.1059	-5.3273	0
T0341	Dimethyl sulfoxide	67-68-5	-16.3863	-16.1062	-0.2801	0
T7445	Acetamide	60-35-5	-16.15	-15.2113	-0.9387	0.0021
T5309	Dimethylamine hydrochloride	506-59-2	-15.7557	-7.4629	-8.2928	0
T0738	Urea	57-13-6	-15.5349	-14.2205	-1.3144	0
T1316	Sodium nitrite	7632-00-0	-15.1891	-3.4107	-11.7784	0
T5606	Formate	64-18-6	-13.622	-8.0159	-5.6061	0
T5300	Ammonium formate	540-69-2	-13.6192	-7.8201	-5.7991	0
T8033	Sodium Thiocyanate	540-72-7	-13.2609	-8.7732	-4.4878	0
